# Making the Best Out of IT: Design and Development of Exergames for Older Adults With Mild Neurocognitive Disorder – A Methodological Paper

**DOI:** 10.3389/fnagi.2021.734012

**Published:** 2021-12-09

**Authors:** Patrick Manser, Eling D. de Bruin

**Affiliations:** ^1^Movement Control and Learning – Institute of Human Movement Sciences and Sport, Department of Health Sciences and Technology, ETH Zürich, Zurich, Switzerland; ^2^Division of Physiotherapy, Department of Neurobiology, Care Sciences and Society, Karolinska Institutet, Stockholm, Sweden; ^3^OST – Eastern Switzerland University of Applied Sciences, St. Gallen, Switzerland

**Keywords:** cognition, development, exercise, exergames, neurosciences, technology, training

## Abstract

**Background:** Utilizing information technology (IT) systems, for example in form of computerized cognitive screening or exergame-based (also called active videogames) training, has gained growing interest for supporting healthy aging and to detect, prevent and treat neurocognitive disorders (NCD). To ameliorate the effectiveness of exergaming, the neurobiological mechanisms as well as the most effective components for exergame-based training remain to be established. At the same time, it is important to account for the end-users’ capabilities, preferences, and therapeutic needs during the design and development process to foster the usability and acceptance of the resulting program in clinical practice. This will positively influence adherence to the resulting exergame-based training program, which, in turn, favors more distinct training-related neurobiological effects.

**Objectives and Methods:** This methodological paper describes the design and development process of novel exergame-based training concepts guided by a recently proposed methodological framework: The ‘Multidisciplinary Iterative Design of Exergames (MIDE): A Framework for Supporting the Design, Development, and Evaluation of Exergames for Health’ (Li et al., 2020).

**Case Study:** A step-by-step application of the MIDE-framework as a specific guidance in an ongoing project aiming to design, develop, and evaluate an exergame-based training concept with the aim to halt and/or reduce cognitive decline and improve quality of life in older adults with mild neurocognitive disorder (mNCD) is illustrated.

**Discussion and Conclusion:** The development of novel exergame-based training concepts is greatly facilitated when it is based on a theoretical framework (e.g., the MIDE-framework). Applying this framework resulted in a structured, iterative, and evidence-based approach that led to the identification of multiple key requirements for the exergame design as well as the training components that otherwise may have been overlooked or neglected. This is expected to foster the usability and acceptance of the resulting exergame intervention in “real life” settings. Therefore, it is strongly recommended to implement a theoretical framework (e.g., the MIDE-framework) for future research projects in line with well-known checklists to improve completeness of reporting and replicability when serious games for motor-cognitive rehabilitation purposes are to be developed.

## Introduction

### Background

Utilizing information technology (IT) systems, for example in form of computerized cognitive screening or exergame-based (also called active videogames) training, has gained growing interest for supporting healthy aging and to detect, prevent and treat neurocognitive disorders (Boletsis and McCallum, 2015; Stanmore et al., 2017). *“An exergame is a videogame that promotes (either via using or requiring) players’ physical movements (exertion) that is generally more than sedentary and includes strength, balance, and flexibility activities”* (Oh and Yang, 2010). Specifically designed and/or implemented games within these training settings are also called ‘serious games’; games developed with a purpose beyond play (Michael and Chen, 2005; Rego et al., 2010). Using exergames for therapeutical interventions complements traditional exercises by using virtual reality, feedback principles and gamification to increase patient motivation and engagement (Matallaoui et al., 2017). This offers *“the unique opportunity for patients to interact in an enriched environment, providing structured, scalable training opportunities augmented by multi-sensory feedback to enhance skill learning and neuroplasticity through repeated practice”* (Aminov et al., 2018). Recent meta-analytic reviews have synthesized that exergame-based training interventions significantly improved various health-related outcomes, including cognitive performance (Howes et al., 2017; Stanmore et al., 2017) and functional physical outcomes (i.e., balance, mobility, exercise capacity) (Howes et al., 2017; Pacheco et al., 2020) in healthy older adults (HOA) as well as in populations with conditions associated with NCD. Furthermore, exergame-based interventions are greatly accepted in individuals with mNCD and increase training adherence and engagement through facilitating training motivation and satisfaction (Zhao et al., 2020).

Exergames are a form of simultaneous motor-cognitive training with incorporated cognitive task demands (Herold et al., 2018). According to the ‘guided-plasticity facilitation’ framework (Fabel and Kempermann, 2008; Kempermann et al., 2010; Herold et al., 2018), acute physical exercise is assumed to enhance brain metabolism and promote neuroplastic processes, whereas these changes in brain plasticity are guided by cognitive stimulation (Fabel and Kempermann, 2008; Kempermann et al., 2010; Joubert and Chainay, 2018). These cognitive and physical exercise demands may exert synergistic effects on brain structural and functional adaptations as well as on cognition, indicating an advantage for combined training against isolated training of either physical or cognitive functions (Lauenroth et al., 2016; Joubert and Chainay, 2018). Indeed, meta-analytic results have recently synthesized simultaneous motor-cognitive training to be the most effective type of training for improving cognitive functioning in HOA (Chen et al., 2020; Gavelin et al., 2021) and older adults with mNCD (Wu et al., 2019; Biazus-Sehn et al., 2020; Gavelin et al., 2021). This is also evidenced by slightly superior effects of exergames on cognitive functioning when compared to physically or cognitively active control interventions (Howes et al., 2017; Stanmore et al., 2017; Wang et al., 2019). However, there are often substantial between-study heterogeneities and inconsistent reporting of interventions, which makes it difficult to draw reliable conclusions about the effectiveness of simultaneous motor-cognitive (Lauenroth et al., 2016; Levin et al., 2017; Tait et al., 2017; Joubert and Chainay, 2018; Yang et al., 2019) or exergame-based (Ogawa et al., 2016; Howes et al., 2017; Stanmore et al., 2017; van Santen et al., 2018; Sokolov et al., 2020; Zhao et al., 2020) training interventions. Further investigations are needed *“to establish the neurobiological mechanisms and effective components of exergames for cognition, and apply this understanding in the development of evidence-based exergame interventions“* (Stanmore et al., 2017) in older adults with NCDs (Lauenroth et al., 2016; Stanmore et al., 2017; van Santen et al., 2018; Haeger et al., 2019; Moreno et al., 2019; Yang et al., 2019; Sokolov et al., 2020; Swinnen et al., 2020b; Zhao et al., 2020).

Besides establishing the most effective components [i.e., qualitative (e.g., type and content of training) and quantitative (e.g., frequency, intensity/complexity, session duration, intervention dose and adaptation over time) exercise and training variables] of exergames for cognition, it is crucial to also account for the users’ perspective when designing and developing novel exergames or training concepts. A recent meta-analysis of training intervention studies in older adults with NCDs has shown that *“improvements in cognitive function were greater in samples that reported greater adherence to the exercise training interventions”* (Panza et al., 2018). Therefore, *“maximizing the effectiveness of interventions to increase and maintain exercise behavior will necessitate an understanding of the dynamic nature of the behavior-change process”* (Robison and Rogers, 1994). In short, adherence to training interventions is key to obtain and preserve health benefits (Robison and Rogers, 1994).

*“Adherence can be intended as ‘maintaining an exercise regimen for a prolonged period following the initial adoption phase”’* (Lox et al., 2016; Di Lorito et al., 2020) and is usually calculated as *“the proportion between the number of sessions attended and the number of sessions offered, reported in percentage”* (Di Lorito et al., 2020). Adherence rates are generally high in exergame-based intervention studies including HOA (Valenzuela et al., 2016; Pacheco et al., 2020) and older adults with NCDs (Swinnen et al., 2020b; Zhao et al., 2020). However, factors and strategies that mediate adherence of exergame-based interventions remain to be established, like indicated by two systematic reviews. Howes et al. (2017) aimed to explore the properties of exergame-based training interventions associated with improved adherence and showed that *“detail of interventions and game design were generally poorly described in terms of promoting adherence, with research in this area still at the stage of testing intervention efficacy, rather than methods of encouraging long-term adherence”* (Howes et al., 2017). Stanmore et al. (2017) stated that the *“variance in participant adherence to the different interventions could not be accounted for in our analyses (as adherence/engagement variables were insufficiently reported across the eligible studies)“* (Stanmore et al., 2017).

From physical training studies, it is known that various factors contribute to the individual’s decision to adhere to a training program in older adults. These factors include a range of program characteristics as well as person-level factors (e.g., demographic factors, health status, physical- and cognitive abilities, psychosocial factors) (Picorelli et al., 2014), but also the attitude toward the value and importance of training, the perceived behavioral control/self-efficacy, the perceived social support, as well as the perceived benefits/barriers and motivation/satisfaction of continued activity (Rhodes et al., 1999). “*Because adherence (or lack thereof) is so crucial to obtain study outcomes, effective strategies and adequate resources should be deployed to address this issue”* (Di Lorito et al., 2020). A recent narrative review synthesized a wide range of support strategies to promote adherence to physical training in older adults with NCD and reported that training interventions *“should be individually tailored, include a learning or adaptation period, provide sufficient information and use phone calls, pedometers, exercise logs and/or reminders as well as supervision and planning to support adherence to the intervention”* (van der Wardt et al., 2017).

When considering the design of computer-based cognitive training programs, the characteristics, needs, and experiences of the target population should be taken into account. A recent systematic review of Diaz Baquero et al. (2021) synthesized, that most often, an end-user centered methodological design is adopted (Diaz Baquero et al., 2021). Ideally, this process fulfills *“the international standards proposed by ISO9241-210 (International Organization for Standardization, 2019) for the development of programs: (1) understanding and specifying the context of use (type, characteristics and tasks of users, and physical or social environment), (2) specifying the user requirements, (3) producing design solutions, and (4) evaluating the design”* (Diaz Baquero et al., 2021). However, it was shown that only half of the studies took the standard ‘specification of user requirements’ into account (Diaz Baquero et al., 2021). Diaz Baquero et al. (2021) concluded that *“it is therefore strongly recommended that future studies use an interactive and participatory design, including end users from the beginning of the pre-prototype development, carrying out evaluations in order to identify user requirements and, in turn, including them in the final development of the prototype”* (Diaz Baquero et al., 2021). Additionally, their finding indicates *“the need to apply this methodology in a more standardized way”* (Diaz Baquero et al., 2021).

Recently, a novel methodological framework was introduced that deems to be suitable to optimally support the process of developing exergames for health in older adults: the ‘Multidisciplinary Iterative Design of Exergames (MIDE): A Framework for Supporting the Design, Development, and Evaluation of Exergames for Health’ (Li et al., 2020). The MIDE-Framework aims to provide comprehensive, integrative, and specific guidance in the design, development, and evaluation of exergames for older adults on basis of an integrated and multifaceted approach (Li et al., 2020). The novelty of the MIDE-Framework is, that is does not only focus on game elements or game development considerations, but also provides a systematic process to guide other relevant stages, such as contextual research and system evaluation (Li et al., 2020).

## Objectives

The aim of this methodological paper is to describe the design and development process of a novel exergame-based training concept for older adults with mNCD guided by the MIDE-Framework.

## Methods

A step-by-step application of the MIDE-framework in an ongoing project aiming to design, develop, and evaluate an exergame-based training concept to halt and/or reduce cognitive decline and improve quality of life in older adults with mNCD is illustrated in a case study.

## Case Study

### Overview

The ongoing project is called ‘Brain-IT’ and started in August 2020. In this project, it is aimed to (a) determine the most suitable components for an exergame-based training in older adults with mNCD; (b) explore novel strategies for a real-time adaptive exergame system to individually tailor exergame demands according to the users’ physical and/or cognitive capabilities; (c) incorporate the acquired knowledge into an exergame-based training concept with the aim to halt and/or reduce cognitive decline and improve quality of life and finally; (d) to evaluate the effectiveness of the resulting training intervention in older adults with mNCD.

According to the MIDE-Framework the project was structured in three phases: Phase 1 – Contextual Research; Phase 2 – Game Design and Development; and Phase 3 – System Evaluation. In phase 1, a synthesis of evidence was combined with qualitative research by performing focus groups in multidisciplinary teams and semi-structured interviews with older adults with mNCD in order to specify a set of design requirements for the exergame-based training concept. In phase 2, possible concepts for the exergame-based training concept were elaborated based on the set of design requirements defined in phase 1. The resulting training concept is currently being tested on its feasibility, usability, and acceptance (Phase 2 - Game Design and Development, Step 4 - Pilot-testing of the Exergame-based Training Concept; see Table 1.

**TABLE 1:** Overview over the three phases of the overall project.

**Overall aim**	**Specific goal**		**Methods/Studies**	**Section**
**Phase 1 – Contextual research (July 2020 – January 2021)**				
Specify design requirements of the exergame-based training concept to be followed in the design and development phase.	Step 1:	Synthesis of Current Knowledge	Literature Review	“Step 1: Literature Review”
	Step 2:	User Modeling	Literature Review, Qualitative Study	“Step 2: User Modeling”
	Step 3:	Determination of Therapeutic Needs	Literature Review, Qualitative Study	“Step 3: Therapeutic Needs”
	Step 4:	Technology Scoping	Collaboration with Dividat AG	“Step 4: Technology Scoping”
	Step 5:	Sustainability Strategy	Collaboration with Dividat AG	“Step 5: Sustainability Strategy”
**Phase 2 – Game Design and Development (February 2021 – March 2022)**				
Development of a fully functional prototype of the exergames and the exergame-based training concept supported by multidisciplinary teamwork including the exergaming industry, game designers, clinical experts, researchers, and the end user.	Step 1:	Game Design	Literature Review, Qualitative Study	“Step 1: Game Design”
	Step 2:	Development and Validation of Adaptation Loop	Systematic Review, Validation Study	“Step 2: Development and Validation of Adaptation Loop”
	Step 3:	Development of the Exergame-based Training Concept		“Step 3: Development of Exergame-based Training Concept”
	Step 4:	Pilot-testing of the Exergame-based Training Concept	Pilot Randomized Controlled Feasibility Study	“Step 4: Playtesting of Exergame-based Training Concept”
	Step 5:	Modification of Exergame- and Intervention Components		“Step 5: Modification of Exergame-based Training Concept”
**Phase 3 – System Evaluation (Start: April 2022)**				
Evaluation of the effectiveness of the resulting exergame-based training concept.	To systematically evaluate the effectiveness and user acceptance of the resulting exergame-based training concept with respect to global cognition as primary outcome and domain-specific cognitive functioning, brain structure and function (measured by magnetic resonance imaging), cardiac vagal modulation (heart rate variability and its associations to neurobiological and cognitive changes), gait and psychosocial factors (e.g., quality of life, motivation, depression, anxiety, stress) as secondary outcomes.		Randomized Controlled Trial	“Phase 3: System Evaluation”

In this project, the exergame training system Dividat Senso (Dividat AG, Schindellegi, Switzerland; CE certification) and its home-based version Dividat Senso Flex are used. In both cases, the system contains a pressure-sensitive platform (1.13 m × 1.13 m; strain gauges measuring at 50 Hz) thereby detecting participants’ position and timing of movements. The stepping platform is divided into five areas: (1) center (home position), (2) front, (3) right, (4) back, and (5) left. Weight-shifting and stepping movements to the four directions enable the interaction and control of the virtual exergame scenarios that are displayed on a screen right in front of the participant. Visual, auditory and somatosensory (vibrating platform) feedback is provided in real-time in order to enrich the game experience.

### Phase 1: Contextual Research

The overall goal of phase 1 is to specify a *“set of design requirements that includes design considerations, accessibility recommendations, user modeling elements, and technological reflections to be followed in the design and development phase”* (Li et al., 2020). Therefore, the project started by a thorough literature review and synthesis of evidence of the current knowledge regarding the effects of cognitive, physical, and combined motor-cognitive training (including exergames) on cognition, brain structure and function, functional physical outcomes, and psychosocial factors in HOAs as well as older adults with NCD. Building on that, a user modeling and determination of therapeutic needs was performed. By combining an evidence-based approach with theoretical and practical workshops in multidisciplinary teams including older adults with mNCD, healthcare professionals, and experts of the exergaming industry, possible concepts for the exergame-based training were elaborated. Finally, the hardware and software requirements to allow the integration of these concepts into exergames suitable for clinical use were determined.

#### Step 1: Literature Review

The project started with synthesizing recent systematic reviews and meta-analyses regarding the effects of cognitive, physical, and combined motor-cognitive training (including exergames) on cognitive functioning, brain structure and function, functional physical outcomes, and psychosocial outcomes (e.g., depressive symptoms, quality of life) in HOAs as well as older adults with NCD. The goal of this step was *“to understand the current theoretical and methodological contributions to the technology advancements, research methodologies, design considerations, and intervention evaluations”* (Li et al., 2020).

##### Cognitive Training

Recent systematic reviews and meta-analyses have synthesized a large body of evidence that cognitive training interventions are effective at improving global cognitive abilities in HOA (Lampit et al., 2014; Toril et al., 2014; Mewborn et al., 2017; Joubert and Chainay, 2018; World Health Organization [WHO], 2019; Gates et al., 2020). For specific cognitive outcomes the findings have been inconsistent. More specifically, recent meta-analyses have synthesized conflicting evidence regarding cognitive training on complex attention [i.e., improvement (Lampit et al., 2014; Toril et al., 2014; Joubert and Chainay, 2018; Bonnechère et al., 2020; Gates et al., 2020) vs. no effect (Sala et al., 2018; Vaportzis et al., 2019; Mansor et al., 2020)], executive function {i.e., improvement (Bonnechère et al., 2020) vs. mixed results [improvements in cognitive inhibition, but no effect on cognitive shifting (Mansor et al., 2020)] vs. no effect (Lampit et al., 2014; Toril et al., 2014; Vaportzis et al., 2019; Gates et al., 2020)}, learning and memory [i.e., improvement (Lampit et al., 2014; Toril et al., 2014; Bonnechère et al., 2020) vs. no effect (Sala et al., 2018; Vaportzis et al., 2019; Gates et al., 2020; Mansor et al., 2020)], visuo-spatial skills [i.e., improvement (Lampit et al., 2014) vs. no effect (Melby-Lervåg et al., 2016; Sala et al., 2018; Vaportzis et al., 2019; Bonnechère et al., 2020)], and working memory [i.e., improvement (Lampit et al., 2014; Melby-Lervåg et al., 2016; Joubert and Chainay, 2018; Bonnechère et al., 2020) vs. no effect (Vaportzis et al., 2019; Gates et al., 2020)]. Although transfer-effects are still debated (Sala and Gobet, 2018; Nguyen et al., 2019), and three meta-analyses have shown smaller improvements in non-trained compared to trained outcomes (Karbach and Verhaeghen, 2014; Melby-Lervåg et al., 2016; Mewborn et al., 2017), these effects were still significant in two of these meta-analyses (Karbach and Verhaeghen, 2014; Mewborn et al., 2017).

In older adults with mNCD or dementia the evidence for the effects of cognitive training remains conflicting. Based on meta-analytic synthesis of evidence, improvements in learning and memory (Hill et al., 2017; Sherman et al., 2017; Bahar-Fuchs et al., 2019; Gates et al., 2019; Zhang et al., 2019) and working memory (Hill et al., 2017; Sherman et al., 2017; Bahar-Fuchs et al., 2019; Gates et al., 2019; Zhang et al., 2019) have been shown, whereas the evidence for cognitive training remains inconsistent for complex attention [i.e.. improvement (Hill et al., 2017; Bahar-Fuchs et al., 2019) vs. no effect (Sherman et al., 2017; Gates et al., 2019)], executive function [i.e., improvement (Sherman et al., 2017; Bahar-Fuchs et al., 2019) vs. no effect (Hill et al., 2017; Gates et al., 2019; Zhang et al., 2019)], global cognition [i.e., improvement (García-Casal et al., 2017; Hill et al., 2017; Mewborn et al., 2017; Sherman et al., 2017; Bahar-Fuchs et al., 2019; Gates et al., 2019; Zhang et al., 2019) vs. no effect (Liang et al., 2018)], verbal fluency {i.e., improvement (Hill et al., 2017; Sherman et al., 2017) vs. mixed effects [improvement in verbal category fluency but not in verbal letter fluency (Bahar-Fuchs et al., 2019)] vs. no effect (Gates et al., 2019)}, or psychosocial factors like anxiety or depression [i.e., improvement (Hill et al., 2017; García-Casal et al., 2017; Chan J.Y.C. et al., 2020) vs. no effect (Liang et al., 2018; Bahar-Fuchs et al., 2019; Gates et al., 2019)], while cognitive training seems to exert no significant effect on visuo-spatial skills (Hill et al., 2017), functional physical performance or activities of daily living (García-Casal et al., 2017; Hill et al., 2017; Bahar-Fuchs et al., 2019; Gates et al., 2019), and quality of life (Bahar-Fuchs et al., 2019; Gates et al., 2019). Reviewed neuroimaging studies have indicated a training induced *“increase in brain activation (particularly in frontoparietal regions) and either an increase or maintenance in connectivity”* (Miotto et al., 2018). This is consistent with another systematic review, that has found *“no effects [*…*] on hippocampal volumes post-training, but cortical thickening and increased gray matter volumes”* (Beishon et al., 2020), suggesting that the brain remains highly plastic in older adults with NCD (Canu et al., 2018; Miotto et al., 2018). An overview of the synthesized meta-analytic results is provided in Supplementary Table S1 in Supplementary File 1.

##### Physical Training

Recent systematic reviews and meta-analyses have shown that physical training interventions improve global cognitive abilities in HOA (Gomes-Osman et al., 2018; Joubert and Chainay, 2018; Northey et al., 2018; Etnier et al., 2019; Sanders et al., 2019; World Health Organization [WHO], 2019). Regarding specific cognitive outcomes, physical training (including aerobic, resistance, and multicomponent training) was shown to significantly improve complex attention (Levin et al., 2017; Gomes-Osman et al., 2018; Joubert and Chainay, 2018; Northey et al., 2018; World Health Organization [WHO], 2019), executive functions (Gomes-Osman et al., 2018; Joubert and Chainay, 2018; Northey et al., 2018; Sanders et al., 2019; World Health Organization [WHO], 2019; Chen et al., 2020), learning and memory (Northey et al., 2018; Sanders et al., 2019), visuo-spatial skills (World Health Organization [WHO], 2019), and working memory (Northey et al., 2018; World Health Organization [WHO], 2019), although these effects didn’t always reach statistical significance and depend on exercise and training variables (Kelly et al., 2014; Northey et al., 2018; Sanders et al., 2019). Additionally, physical training interventions were shown to reduce fall rates (Sherrington et al., 2017) and exert a positive effect on cardiac autonomic control (Raffin et al., 2019) and hippocampal volumes (Firth et al., 2018) in HOA.

Systematic reviews and meta-analyses for the effects of physical training on cognition in older adults with mNCD or dementia are less consistent and suggest improvements in executive functioning (Sanders et al., 2019; Biazus-Sehn et al., 2020; Chen et al., 2020; Zhou et al., 2020) and visuo-spatial skills (Zhou et al., 2020), whereas no significant changes in complex attention (Kelly et al., 2014; Biazus-Sehn et al., 2020; Law et al., 2020), and mixed findings for global cognition [i.e., improvement (Groot et al., 2015; Ströhle et al., 2015; Gomes-Osman et al., 2018; Northey et al., 2018; Panza et al., 2018; Etnier et al., 2019; Jia et al., 2019; Sanders et al., 2019; Wang et al., 2019; Biazus-Sehn et al., 2020; Law et al., 2020; Zhou et al., 2020) vs. no effect (Kelly et al., 2014; Forbes et al., 2015; Liang et al., 2018)], language [i.e., improvement (Zhou et al., 2020) vs. no effect (Kelly et al., 2014; Biazus-Sehn et al., 2020; Law et al., 2020)], learning and memory {i.e., improvement (Zhou et al., 2020) vs. mixed effects [i.e., improvement in delayed recall and no effect on immediate recall (Biazus-Sehn et al., 2020)] vs. no effect (Kelly et al., 2014; Gomes-Osman et al., 2018; Sanders et al., 2019; Law et al., 2020)}, and working memory [i.e., improvement (Law et al., 2020) vs. no effect (Kelly et al., 2014; Gomes-Osman et al., 2018; Biazus-Sehn et al., 2020)] were synthesized. Additionally, meta-analytic results have synthesized significant improvements in activities of daily living (Forbes et al., 2015; Groot et al., 2015; Lam et al., 2018), balance (Lam et al., 2018), behavioral problems (Law et al., 2020), endurance (Lam et al., 2018), gait (i.e., step length and walking speed) (Lam et al., 2018), and mobility (Lam et al., 2018). Furthermore, positive effects on depressive symptoms (Forbes et al., 2015) and inconsistent findings on fall rate [improvement (Sherrington et al., 2017) vs. no effect (Lam et al., 2018)] were found. Nonetheless, the preventative effect of physical training seems to be limited, as analyzed by the meta-analysis of de Souto Barreto et al. (2018) that has found no significant effect on cognitive decline and risk of onset of mild or major NCD.

Moreover, several systematic reviews have indicated positive effects of physical training on brain structure and function. The systematic reviews of Firth et al. (2018), Joubert and Chainay (2018), Haeger et al. (2019), Herold et al. (2019b), Marinus et al. (2019), and Stillman et al. (2020) have indicated positive effects of physical training on structural (i.e., overall gray and white matter volume, hippocampal volume) and functional (i.e., functional connectivity, cerebral blood flow, task-related oxygenation, concentration of neurochemicals) changes in the brain of HOA (Firth et al., 2018; Joubert and Chainay, 2018; Haeger et al., 2019; Herold et al., 2019b; Marinus et al., 2019; Stillman et al., 2020). There are already meta-analytic results that corroborate some of these effects by showing that aerobic training slows down the decline in hippocampal volume (Firth et al., 2018) and strength training or combined training increase peripheral BDNF concentration (Marinus et al., 2019) that might be related to changes in cognitive abilities (Joubert and Chainay, 2018). For older adults with mNCD or dementia, only a small number of studies examining the interrelation of structural and functional brain changes with changes in cognitive performance is available (Canu et al., 2018; Haeger et al., 2019; Herold et al., 2019b; Stillman et al., 2020). Aerobic training seems to exert a protective effect on structures vulnerable to neurodegenerative processes including *“frontal, temporal and parietal regions, such as the hippocampal/parahippocampal region, precuneus, anterior cingulate and prefrontal cortex”* (Haeger et al., 2019; Stillman et al., 2020). Resistance training was additionally shown to ameliorate resting state functional connectivity (i.e., *“among the posterior cingulate cortex, the left inferior temporal lobe, and the anterior cingulate cortex and between the hippocampus and the right middle frontal lobe”*) (Herold et al., 2019b). An overview of the synthesized meta-analytic results is provided in Supplementary Table S2 in Supplementary File 1.

##### Motor-Cognitive Training

When considering specific training types, aerobic and multicomponent physical training were shown to be beneficial training types (Groot et al., 2015; Panza et al., 2018; Sanders et al., 2019; Law et al., 2020), while cognitively engaging training appears to have the strongest effect on cognition (Lauenroth et al., 2016; Howes et al., 2017; Levin et al., 2017; Stanmore et al., 2017; Joubert and Chainay, 2018; van Santen et al., 2018; Stojan and Voelcker-Rehage, 2019; Wang et al., 2019; Wu et al., 2019; Biazus-Sehn et al., 2020; Chen et al., 2020; Gallou-Guyot et al., 2020; Mansor et al., 2020; Zhu et al., 2020; Gavelin et al., 2021). These findings are consistent with the ‘guided-plasticity facilitation’ framework (Fabel and Kempermann, 2008; Kempermann et al., 2010; Herold et al., 2018): Acute physical exercise is assumed to enhance brain metabolism and promote neuroplastic processes, whereas these changes in brain plasticity are guided by cognitive stimulation (Fabel and Kempermann, 2008; Kempermann et al., 2010; Joubert and Chainay, 2018). Importantly, the systematic reviews of Joubert and Chainay (2018) and Lauenroth et al. (2016) have suggested that cognitive and physical training demands may exert synergistic effects on brain structural and functional adaptations as well as on cognition, indicating an advantage for combined training (Lauenroth et al., 2016; Joubert and Chainay, 2018). Therefore, one might assume, that combined motor-cognitive training is more effective compared to isolated physical or cognitive training.

Multiple meta-analyses in HOA have synthesized evidence for significant improvements in executive functions (Howes et al., 2017; Chen et al., 2020; Mansor et al., 2020) and working memory (Mansor et al., 2020) in response to sequential or simultaneous motor-cognitive training while the evidence for global cognition [i.e., improvement (Stanmore et al., 2017; Northey et al., 2018; Chen et al., 2020; Zhu et al., 2020; Gavelin et al., 2021) vs. no effect (Wu et al., 2019)] and learning and memory {i.e., mixed findings [improvement in updating memory but no effect on delayed memory (Mansor et al., 2020)]} remains conflicting, and no significant effects were synthesized for complex attention (Vaportzis et al., 2019; Mansor et al., 2020), and verbal fluency (Stanmore et al., 2017). Additionally, improvements in balance (Howes et al., 2017; Pacheco et al., 2020) and functional exercise capacity (Howes et al., 2017) have been synthesized while the evidence for mobility remains conflicting [i.e., improvement (Pacheco et al., 2020; Gavelin et al., 2021) vs. no effect (Howes et al., 2017)] and no significant effects have been synthesized for activities of daily living (Corregidor-Sánchez et al., 2020). When considering meta-analytic results for exergaming specifically, significantly larger improvements in complex attention (Stanmore et al., 2017), executive functions (Howes et al., 2017; Stanmore et al., 2017), global cognition (Stanmore et al., 2017), visuospatial processing (Stanmore et al., 2017), and also functional physical outcomes (i.e., balance, mobility) (Howes et al., 2017), and fear of falling (Howes et al., 2017), but not activities of daily living (Corregidor-Sánchez et al., 2020) or functional exercise capacity (Howes et al., 2017) have been synthesized compared to physically or cognitively active control interventions.

For older adults with mNCD or dementia, significant improvements in complex attention (Chan J.S.Y. et al., 2020), global cognition (Stanmore et al., 2017; Wang et al., 2019; Wu et al., 2019; Biazus-Sehn et al., 2020; Chan J.S.Y. et al., 2020; Zhu et al., 2020; Gavelin et al., 2021), learning and memory (Biazus-Sehn et al., 2020; Chan J.S.Y. et al., 2020), and visuo-spatial skills (Chan J.S.Y. et al., 2020) have been meta-analytically synthesized, whereas there is conflicting evidence for executive functioning [i.e., improvement (Biazus-Sehn et al., 2020) vs. no effect (Chan J.S.Y. et al., 2020)] and language (Zhu et al., 2020), and no effects have been synthesized for working memory (Chan J.S.Y. et al., 2020). Additionally, improvements in physical outcomes (e.g., mobility, balance) (Gavelin et al., 2021) and psychosocial factors (i.e., neuropsychiatric symptoms, depression, quality of life) (Gavelin et al., 2021) have been synthesized. For exergames specifically, significantly larger increases in global cognitive function have been synthesized when compared to physically and cognitively active control interventions (Stanmore et al., 2017). Moreover, exergame-based training interventions are greatly accepted in individuals with mNCD and increase training adherence and engagement through facilitating training motivation and satisfaction (Zhao et al., 2020).

Therefore, especially exergaming seems to be a promising type of simultaneous motor-cognitive training for improving cognition in cognitively impaired individuals, although the optimal training components (e.g., type of exergame, training intensity and duration) remain to be established (Stanmore et al., 2017; van Santen et al., 2018; Stojan and Voelcker-Rehage, 2019; Swinnen et al., 2020b; Zhao et al., 2020; Gavelin et al., 2021). The positive effects of simultaneous motor-cognitive training on cognition may be explained by neurophysiological changes of the brain, including changes in hemodynamics, electrophysiology, or neurotrophic factors (Lauenroth et al., 2016; Tait et al., 2017; Joubert and Chainay, 2018; Haeger et al., 2019; Stojan and Voelcker-Rehage, 2019; Yang et al., 2019). The Systematic Review of Muiños and Ballesteros (2021) concluded that motor-cognitive training (more specifically: dancing) *“can be effective for inducing neuroplasticity and that the duration of the intervention and the intensity of the dancing exercise might be important to induce brain changes and cognitive improvements”* (Muiños and Ballesteros, 2021). For exergames specifically, Stojan and Voelcker-Rehage (2019) concluded in their systematic review, that “*neurophysiological changes with regard to exergaming (within exergamers or by group x time effects) were present in all corresponding studies (either on hemodynamics, electrophysiology, or neurotrophic factors) indicating brain plastic adaptations in response to exergaming”* (Stojan and Voelcker-Rehage, 2019). Nonetheless, the evidence of structural and functional changes in the brain in response to motor-cognitive training in mNCDs is limited to single studies with inconsistent outcomes (Canu et al., 2018; Haeger et al., 2019; Yang et al., 2019). Further investigations are needed *“to establish the neurobiological mechanisms and effective components of exergames for cognition, and apply this understanding in the development of evidence-based exergame interventions“* (Stanmore et al., 2017) in older adults with NCDs (Lauenroth et al., 2016; Stanmore et al., 2017; van Santen et al., 2018; Haeger et al., 2019; Moreno et al., 2019; Yang et al., 2019; Sokolov et al., 2020; Swinnen et al., 2020b; Zhao et al., 2020). An overview of the synthesized meta-analytic results is provided in Supplementary Table S3 in Supplementary File 1.

#### Step 2: User Modeling

The second step of the project is aimed at determining the *“preferences and needs of the targeted user group from a multi-disciplinary perspective in order to optimize the exergaming experience. In addition to general aspects such as demographics, capability, characteristics, hobbies, and motivators for playing, exergame-specific user models should also include other attributes like the facilitators and barriers to physical activity engagement”* (Li et al., 2020). With this regard, the clinical picture, epidemiology, risk factors, prevention, and therapy options were summarized based on a literature search of the current evidence. The capabilities, treatment experience- and preferences as well as motivators for training of older adults with mNCD were determined based on a synthesis of evidence in combination with the results of a qualitative study. Our qualitative study included: (1) focus groups with experts/healthcare professionals; and (2) individual semi-structured interviews with older adults with mNCD. With this regard, 5 – 10 experts/healthcare professionals with a variety in age, gender, educational level and experience in therapy of older adults with mNCD and 5 – 10 older adults with mNCD with variations in age, education, training habits and technology use were purposively recruited. The focus groups and individual semi-structured interviews were both organized as semi-structured interviews along an interview guide and were conducted between November 2020 and January 2021 (Manser et al., 2022a^1^).

##### Clinical Picture

The clinical picture of mNCD represents an intermediate stage of cognitive impairment between the normal aging process and dementia (Petersen et al., 1997, 2014; American Psychiatric Association, 2013; Lindbergh et al., 2016; Sanford, 2017; Janelidze and Botchorishvili, 2018; World Health Organization [WHO], 2018). It is diagnosed on basis of: *“(A.) Evidence of modest cognitive decline from a previous level of performance in one or more cognitive domains (complex attention, executive function, learning and memory, language, perceptual motor, or social cognition) based on: (1) Concern of the individual, a knowledgeable informant, or the clinician that there has been a mild decline in cognitive function; and (2) A modest impairment in cognitive performance, preferably documented by standardized neuropsychological testing or, in its absence, another quantified clinical assessment. (B.) The cognitive deficits do not interfere with capacity for independence in everyday activities (i.e., complex instrumental activities of daily living such as paying bills or managing medications are preserved, but greater effort, compensatory strategies, or accommodation may be required). (C.) The cognitive deficits do not occur exclusively in the context of delirium. (D.) The cognitive deficits are not better explained by another mental disorder (e.g., major depressive disorder, schizophrenia)”* (American Psychiatric Association, 2013). Older adults with mNCD can also be referred to as individuals with mild cognitive impairment (MCI). *“The main difference between MCI and mild NCD is that the research work that led to the construct of MCI took place in the context of geriatric populations (even though age was not part of the definition of MCI), whereas mNCD encompasses acquired cognitive disorders of all age groups”* (Stokin et al., 2015). Older adults with mNCD can be classified into four subtypes, according to the presence or absence of memory impairment (i.e., amnestic or non-amnestic MCI) and whether multiple cognitive domains are affected (single domain or multiple domains MCI) (Petersen, 2011; Roberts and Knopman, 2013; Janelidze and Botchorishvili, 2018). Deteriorations in episodic memory and executive function represent the most prevalent cognitive impairments (Chehrehnegar et al., 2020). The objective cognitive decline is associated with structural changes in the brain, including declines in gray matter volume and alterations in the connectivity of the temporal, parietal, and frontal lobes, the amygdala, fusiform gyrus, as well as the cingulate, parietal and occipital lobes and the insula (Schuff and Zhu, 2007; Janelidze and Botchorishvili, 2018; Chehrehnegar et al., 2020). Especially the structural changes in the hippocampus predict the conversion of MCI to dementia (Kantarci et al., 2005; Apostolova et al., 2006).

##### Epidemiology

The global prevalence of mNCD increases with age, is more than twice as high than for dementia, and ranges between 3 and 54% depending on the clinical classification (Petersen et al., 2009, 2014, 2018; Hu et al., 2017; Janelidze and Botchorishvili, 2018; Parnetti et al., 2019). The global incidence of MCI is estimated to increase from 2% at age 75 – 79 increasing up to 7% (Janelidze and Botchorishvili, 2018; Gillis et al., 2019). In the general population, approximately 4.9% of individuals diagnosed with MCI convert to dementia every year, whereas the adjusted annual conversion rate in clinical MCI populations is 9.6% (Mitchell and Shiri-Feshki, 2009). Fortunately, between 14% (clinical populations) and 31% (community-based cohort) revert to normal cognitive functioning for their age (Malek-Ahmadi, 2016; Kasper et al., 2020). Nonetheless, a recent meta-analysis reported a pooled progression rate of 34%, more than twice as high as the pooled reversion rate of 15% (Hu et al., 2017). This dichotomy between conversion to dementia and reversion to normal cognition suggests the presence of modifiable risk factors contributing to this cognitive decline (Sanford, 2017; Kasper et al., 2020).

##### Risk Factors, Prevention and Treatment Options

Age is considered to be the strongest risk factor for developing mNCD (Hu et al., 2017; Sanford, 2017; Janelidze and Botchorishvili, 2018; Levine et al., 2018; Parnetti et al., 2019). Other risk factors include the male sex (Petersen et al., 2010; Roberts et al., 2012; Hu et al., 2017), the presence of the apolipoprotein E allele (Caselli et al., 2009), a family history of cognitive impairment (Ng et al., 2016), the presence of vascular risk factors (i.e., metabolic syndrome, hypertension, hyperlipidemia, coronary heart disease, diabetes mellitus, or stroke) (Roberts et al., 2014; Vassilaki et al., 2015; Pal et al., 2018), or a physically or cognitively sedentary lifestyle (Verghese et al., 2006; Geda et al., 2010). Hence, changes in lifestyle that increase physical activity and/or reduce vascular risk factors are powerful protectors for brain atrophy and cognitive decline (Erickson et al., 2010; Sofi et al., 2011; Beydoun et al., 2014; Blondell et al., 2014; Carvalho et al., 2014; Beckett et al., 2015; Guure et al., 2017; Lee, 2018; Cunningham et al., 2020). When considering therapy options for incident MCI, physical and cognitive training were even shown to outperform pharmacological therapies (Liang et al., 2018). Indeed, *“there is currently no effective pharmacological intervention for MCI”* (Kasper et al., 2020). The evidence for pharmacological treatment options (e.g., cholinesterase inhibitors, antihypertensive-, anti-inflammatory or lipid-lowering medication, or hormone therapies) and nutritional supplements is largely insufficient and does not support its use for improving cognitive performance, slowing down cognitive decline or reducing the risk for developing dementia (Cooper et al., 2013; Fitzpatrick-Lewis et al., 2015; Ströhle et al., 2015; Farina et al., 2017; Butler et al., 2018; Fink et al., 2018). Consequently, it was suggested to focus on multi-domain treatment strategies including physical training and cognitive stimulation (Sanford, 2017; Kasper et al., 2020). In fact, *“a burgeoning body of evidence suggests that targeting modifiable risk factors in midlife may hold promise for mitigating or even preventing Alzheimer’s disease and related dementias in later life”* (de Oliveira et al., 2015; Lehert et al., 2015; Chuang et al., 2016; Iadecola et al., 2016; Smith, 2019). As already stated in section “Motor-Cognitive Training,” especially exergaming seems to be an effective mode of simultaneous motor-cognitive training for improving cognitive functioning in older adults with mNCD.

##### Capabilities

According to the definition of mNCD, capacity for independence in everyday activities is preserved, despite modest (i.e., for mild NCD, performance typically lies in the 1–2 standard deviation range; between the 3rd and 16th percentiles) deteriorations in cognitive functioning (American Psychiatric Association, 2013). When considering the results of our qualitative study, the most often described impairments referred to cognitive functioning including impairments in executive function, complex attention, learning and memory, visuo-spatial skills, language, and social cognition from the experts’ viewpoint. These cognitive changes were also described to affect psychosocial factors, mainly by causing psychological distress and feelings of insecurity, leading patients trying to hide their impairments. In line with the experts’ viewpoint, cognitive deteriorations were frequently described to mainly affect learning and memory, complex attention, and executive function, while no serious restrictions in physical capabilities, mobility, and ADLs were mentioned by the patients themselves. However, from patient’s perspective, the consequences of their cognitive decline on psychosocial factors were most prominent, mainly by causing psychological distress, feelings of insecurity, and depression (Manser et al., 2022a) (see text footnote 1).

##### Treatment Preferences

The findings of our qualitative study suggested that - according to the experience of the experts/healthcare professionals - solely cognitive forms of training (e.g., computerized cognitive training) or physical training (e.g., resistance training) were often experienced as boring in the long run by older adults with mNCD. More integrative forms of training including gamified tasks close to everyday life, multimodal animation, and acoustic feedback were reported to be preferred by patients. From a patient’s perspective, computerized cognitive training was reported to be perceived as challenging, fun, and enjoyable. Although being perceived as useful, patients reported to be insecure about the effectiveness of computerized cognitive training (Manser et al., 2022a) (see text footnote 1).

The previous experience in the use of exergames (i.e., Dividat Senso) with patients with mNCD was described as good by the experts in our qualitative study. The simple and clear design structures of the games were reported to be highly appreciated by patients and to promote good comprehensibility of the tasks (Manser et al., 2022a) (see text footnote 1). This is also consistent with the literature, showing that exergame-based training interventions are greatly accepted in individuals with mNCD and increase training adherence and engagement through facilitating training motivation and satisfaction (Zhao et al., 2020). Accordingly, adherence to exergame-based training interventions is typically high in older adults with NCD (Swinnen et al., 2020b; Zhao et al., 2020). Nonetheless, various minor usability issues were reported in our qualitative study that need to be considered when developing a training concept specifically for older adults with mNCD. These usability problems include some minor issues in the interaction with the exergame training system Dividat Senso (e.g., unintendingly walk off the middle-plate without noticing the feedback on the screen), but were mainly related to capabilities of older adults with mNCD. Patients were often described to be cognitively overloaded when trying out new exergames or when in unexpected situations or experiencing technical errors. Additionally, some games were reported to start at an already (too) challenging level for older adults with mNCD and progress too fast while there is a limited range of games and/or adaptability of task demands at the lower end of difficulty levels. This was mentioned to mainly be apparent for the cognitive task demands (e.g., game speed, task complexity) while the physical exercise intensity is often low and could be increased. Overwhelming task demands were described to cause frustration and/or refusal of playing games, although the feedback mechanisms to indicate errors work rather subtle. On the other hand, exergames that are perceived as being too easy lead to boredom. Therefore, the findings of the qualitative study illustrated that applying an optimal challenge is central to promote the use of exergames in patients with mNCD over the long-term (Manser et al., 2022a) (see text footnote 1).

##### Motivators for Treatment

The ‘Self-determination Theory’ (Deci and Ryan, 2002) has demonstrated considerable efficacy in explaining exercise motivation and behavior (Hagger and Chatzisarantis, 2008). It accounts for the quality of different levels of motivational regulation in physical activity settings and is considered useful to gain a better understanding and promoting training motivation, enjoyment, and adherence (Hagger and Chatzisarantis, 2007; Murcia et al., 2008; Wilson et al., 2008; Teixeira et al., 2012). More autonomous forms of motivation refer to engagement in a task based on intrinsic motivators (e.g., enjoyment, personal importance). This is considered advantageous and linked with positive behavioral changes (e.g., in exercise) (Ryan and Deci, 2000). The ‘Self-determination Theory’ (Deci and Ryan, 2002) is in line with multiple empirical observations that predicted favorable exercise and training behavior with more autonomous forms of motivational regulation in healthy adults (Duncan et al., 2010; Teixeira et al., 2012; Wilson et al., 2012; Friederichs et al., 2015), HOA (Teixeira et al., 2012; Devereux-Fitzgerald et al., 2016; Gummelt, 2017; Behzadnia et al., 2020), and also in clinical populations like stroke patients (Swanson and Whittinghill, 2015), patients with cardiovascular disease (Russell and Bray, 2009), or patients with NCD (Barrado-Martín et al., 2021). For example, in a large cohort of regular exercisers, more autonomous forms of motivation (i.e., identified and integrated regulation) predicted training frequency, intensity, and duration (Duncan et al., 2010). Depending on the population, different factors determine how more autonomous motivation can be promoted. A small case-control study with a balance exergaming platform evaluated that *“older adults were more intrinsically motivated by the joy of playing and extrinsically motivated by the perceived health effects (physical and cognitive), with less regard for the in-game rewards”* (Subramanian et al., 2019). For patients with NCDs specifically, a new theoretical model, the ‘PHYT in dementia’ (Di Lorito et al., 2019), was recently introduced. It includes both individual-level and environment-level constructs with the aim to *“inform effective interventions to promote physical activity”* (Di Lorito et al., 2019) in patient with NCDs. It proposes that self-efficacy including embarrassment (e.g., supervision of activity had a negative impact on engagement in the intervention), personal concerns (e.g., fear of falling) and routine (e.g., flexible integration of physical activity intervention into daily life regarding place and time of performance), as well as appropriate challenge are considered additional key elements for promoting physical activity behavioral changes (Di Lorito et al., 2019). To account for these factors, especially for the preference that *“the routine can be performed at home and at different times during the day”* (Di Lorito et al., 2019), a detailed awareness of participants motivators is required, since self-determined motivation may be a central aspect for the adherence in home-based training programs (Russell and Bray, 2009).

This is consistent with the findings of our qualitative study, showing that the most frequently described motivators can be classified as intrinsically regulated motivators that are directly related to the exergames. It was described that excitement, enjoyment or fun is perceived as a central motivator for performing exergames that is maintained by the inclusive character of exergames that is supported by specific game characteristics. More specifically, mainly game tasks or -designs close to everyday life or with a personal relation/memory including music/sound effects, animal/plants, landscapes, or colors were reported to promote intrinsic motivation. Additionally, patients were described to be intrinsically motivated by gamification and the feeling of being optimally challenged. However, when task demands get too high or too low patients’ have been observed to promptly lose their willingness to perform the exergames (Manser et al., 2022a) (see text footnote 1).

#### Step 3: Therapeutic Needs

In step 3, it was aimed to: (1) *“specify the users’ fitness goals, training settings, and outcome measures”* (Li et al., 2020); and (2) *“determine the core components of the training plan (e.g., type of exercise, target outcomes, based on FITT-VP: Frequency, Intensity, Type, Time, Volume, and Progression model)”* (Li et al., 2020). To specify the patients’ training goals and -settings and to support the determination of the most suitable exergame intervention components, we relied on the integration of the outcomes of (a) a comprehensive literature synthesis regarding moderating effects of training interventions on training efficacy, and (b) the qualitative study including semi-structured interviews with older adults with mNCD and focus groups with healthcare professionals (as described above).

##### Training Goals and Outcomes

According to the findings of our qualitative study, mainly cognitive functioning should be targeted in the training intervention in experts’ viewpoint, while also addressing ADLs and mobility, physical capabilities, and accounting for psychosocial factors. When asking experts about the training goals of patients, improving ADLs and mobility were stated most frequently besides cognition and physical functioning. Additionally, psychosocial factors were reported that include socializing or just having fun. This is consistent with patients’ viewpoint that most frequently reported quality of life and independence as primary training goals (Manser et al., 2022a) (see text footnote 1).

When comparing these perspectives with the literature, similar results have been synthesized. An online survey in 2018 evaluated the *“outcome and treatment preferences of patients and caregivers who had completed a multicomponent behavioral intervention for mild cognitive impairment (MCI)”* (Smith et al., 2018). The most important outcome priority for MCI patients was quality of life, followed by self-efficacy, depression, basic Activities of Daily Living (ADL), memory-based ADL, anxiety and memory performance (Smith et al., 2018). A better self-efficacy is expected to improve perceived quality of life (Langer et al., 2019).

##### Core Components of the Training Plan

To get a better understanding of previous investigations and the dose-response relationships of different qualitative (i.e., type and content of training) and quantitative (i.e., frequency, intensity/complexity, session duration, intervention dose and adaptation over time) exercise and training variables, recent meta-analytic results were synthesized (Supplementary Table S4 in Supplementary File 1) and summarized (Table 2) and complemented with additional evidence if required to make an informed decision. These findings were then used to guide the formulation of requirements for an optimal intervention design in line with the findings of the qualitative study, to ensure that the resulting intervention design is also considered feasible based on experts’ and patients’ viewpoint.

**TABLE 2:** Moderating effects of exercise and training parameters on the effectiveness of cognitive, physical, and cognitive-motor training in healthy older adults and older adults with mild neurocognitive disorder.

**Training parameter**		**Cognitive Training**		**Physical Training**		**Motor-Cognitive Training**		**Preferred Choice for Brain-IT**
		**No effect**	**(near) sign. moderating effect**	**No effect**	**(near) sign. moderating effect**	**No effect**	**(near) sign. moderating effect**	
Frequency	mNCD	Bahar-Fuchs et al., 2019	• Higher (>3x/week) (Bahar-Fuchs et al., 2019)	Groot et al., 2015	• Higher (≥ 4x/week) (Sanders et al., 2019)	NR	NR	High frequency (≥5x/week)
	HOA	NR	• Lower [≤2x/week (Mewborn et al., 2017), ≤3x/week (Lampit et al., 2014)]	NR	• Higher [≥2x/week (Sanders et al., 2019), ≥3x/week (Chen et al., 2020), ≥5x/week (Northey et al., 2018)]	Mansor et al., 2020	• Higher [≥3x/week (Chen et al., 2020), ≥5x/week (Northey et al., 2018)]	
					• Lower (≤3x/week) (Jia et al., 2019)		
Intensity/Complexity	mNCD	NR	NR	Sanders et al., 2019; Chen et al., 2020	• Moderate intensity (Biazus-Sehn et al., 2020)	Chen et al., 2020	• Moderate physical exercise intensity (Biazus-Sehn et al., 2020)	Physical load: moderate intensity motor complexity: high challenge cognitive load: unknown
					• moderate to high intensity (Law et al., 2020)		
	HOA	Toril et al., 2014	NR	Sanders et al., 2019	• Moderate to vigorous (Northey et al., 2018)	NR	• high motoric challenge (Sherrington et al., 2017)	
					• high motoric challenge (Sherrington et al., 2017)		
Type (of training)	mNCD	García-Casal et al., 2017; Bahar-Fuchs et al., 2019; Chan J.Y.C. et al., 2020)	• Computer-based (García-Casal et al., 2017)	Law et al., 2020	• Aerobic training (Groot et al., 2015; Panza et al., 2018)	Wu et al., 2019	• Simultaneous training (Gavelin et al., 2021)	Individually applied simultaneous motor-cognitive training
			• Individual training (Sherman et al., 2017)		• Multicomponent (Groot et al., 2015; Sanders et al., 2019)	• Combined training (Biazus-Sehn et al., 2020)	
	HOA	NR	• Video-game based training (Lampit et al., 2014; Toril et al., 2014)	Sanders et al., 2019	• Multicomponent (Northey et al., 2018)	NR	• Simultaneous training (Chen et al., 2020; Gavelin et al., 2021)	
						• Exergaming (Mansor et al., 2020)	
Time (exercise duration)	mNCD	NR	NR	NR	• Shorter (≤30 min) (Sanders et al., 2019)	NR	NR	≤30 min
	HOA	Lampit et al., 2014	• Shorter (≤30 min) (Mewborn et al., 2017)	Raffin et al., 2019; Sanders et al., 2019; Chen et al., 2020	• Shorter (≤30 min) (Jia et al., 2019)	Chen et al., 2020; Mansor et al., 2020	• Shorter (Mansor et al., 2020)	
					• Longer (≥45 min) (Northey et al., 2018)		
Duration (of the intervention)	mNCD	Bahar-Fuchs et al., 2019	• Longer (≥3 months) (Bahar-Fuchs et al., 2019)	Sanders et al., 2019; Wu et al., 2019	NR	Wu et al., 2019	NR	≥12 weeks
	HOA	Bonnechère et al., 2020	• Shorter (≤6 weeks) (Toril et al., 2014)	Sanders et al., 2019	• Shorter (≤12 weeks) (Northey et al., 2018; Chen et al., 2020)	Biazus-Sehn et al., 2020	• Longer (≥12 weeks) (Stanmore et al., 2017)	
					• Longer (>16 weeks) (Jia et al., 2019)	• Shorter (≤12 weeks) (Biazus-Sehn et al., 2020; Chen et al., 2020; Gavelin et al., 2021)	
Volume (i.e., total intervention/exercise time)	mNCD	Sherman et al., 2017; Zhang et al., 2019	NR	Law et al., 2020	• Higher [≥24 h (Law et al., 2020)]	NR	• Moderate (60 – 120 min/week) (Wu et al., 2019)	Moderate (60 – 120 min/week)
				• Moderate (60 – 120 min/week) (Wu et al., 2019)			
				• Lower (Biazus-Sehn et al., 2020) (≤2h/week) (Jia et al., 2019)			
	HOA	Lampit et al., 2014	• Higher (≥20 h, ≥20 sessions) (Mewborn et al., 2017)	Raffin et al., 2019	• Higher [≥3 h/week (Sherrington et al., 2017)]	Mansor et al., 2020	• Higher volume (≥120 min/week) (Howes et al., 2017)	
Progression and Periodization	mNCD	NR	NR	NR	NR	NR	NR	Unclear
	HOA	NR	NR	NR	NR	NR	NR	
Variability/Variation	mNCD	NR	NR	NR	NR	NR	NR	Unclear
	HOA	NR	• Fewer games (≤6 games) tend to be beneficial (Toril et al., 2014)	NR	NR	NR	NR	
Specificity	mNCD	Bahar-Fuchs et al., 2019; Zhang et al., 2019	Multi-domain training (Sherman et al., 2017; Bahar-Fuchs et al., 2019) including memory (Sherman et al., 2017) -memory-specific training	NR	NR	NR	NR	Focus on working memory and memory training as part of a multi-domain training
	HOA	NR	Multi-domain training (Mewborn et al., 2017; Sherman et al., 2017) including working memory (Mewborn et al., 2017) and memory (Mewborn et al., 2017; Sherman et al., 2017) -memory-specific training	NR	NR	NR	NR	

*HOA, healthy older adults; NR, not reported; mNCD, mild neurocognitive disorder.*

*Qualitative Training Components*. Based on the synthesized (Supplementary Table S4 in Supplementary File 1) and summarized (Table 2) evidence on moderating effects of different training interventions, combined (preferably simultaneous) motor-cognitive training can be considered the most effective type of training for improving cognition in HOA (Chen et al., 2020; Gavelin et al., 2021) and older adults with mNCD (Biazus-Sehn et al., 2020; Gavelin et al., 2021). One approach to apply simultaneous motor-cognitive training is exergaming. The currently available evidence suggests slightly superior effects of exergame training on cognitive abilities when compared to physically or cognitively active control interventions (Howes et al., 2017; Stanmore et al., 2017). Moreover, exergame-based training interventions are greatly accepted in individuals with mNCD and increase training adherence and engagement through facilitating training motivation and satisfaction (Zhao et al., 2020). Therefore, using exergames is the most promising approach for the training intervention.

The specific mode of motor-cognitive exergame training may be motor-cognitive training with incorporated cognitive tasks (Herold et al., 2018). The content of the exergames should mainly focus on working memory and memory training as part of a multi-domain training program (Mewborn et al., 2017; Sherman et al., 2017; Bahar-Fuchs et al., 2019). Furthermore, the exergames should integrate specific tasks demanding cognitive flexibility that engage multiple cognitive domains (e.g., related to spatial memory) at the same time (Buitenweg et al., 2012; Herold et al., 2018). Preferably, the specific components of the exergame interventions are tailored to the individual, based on objective assessments of individual capabilities such as cognitive abilities, physical fitness, motor abilities, as well as demographic characteristics (e.g., age, gender, health status, and the socioemotional status including motivation, mood, or stress) (Herold et al., 2018). Furthermore, the preferred postural modality in which exercise is performed should be in a vertical body loading position (Swinnen et al., 2021). Exercise performed in standing position that requires a changing base of support to play the games better meets the specifics for training postural control (Tahmosybayat et al., 2018) and puts a higher demand on spatial processing demands (Dodwell et al., 2019) next to enhancing both processing speed and attentional selectivity (Rosenbaum et al., 2017). Such effects of improved balance and executive functions are not observed for exercise performed pedaling a bicycle in a seated position (Karssemeijer et al., 2019a, b) possibly due to a lack of a dynamic influence on visual working memory performance (Dodwell et al., 2019). In this context an ecologically more valid motor-cognitive training type that allows for controllable activities and to incorporate complexity, novelty, and diversity in the training design, can be enabled by virtual reality-based video gaming (Moreau and Conway, 2014).

*Quantitative Training Components*. The analysis of moderating variables of training parameters influencing the effectiveness of the interventions (Tables 2, Supplementary Table S4 in Supplementary File 1) revealed several preferences. Based on meta-analytical results from motor-cognitive training in older adults with mNCD, a moderate physical training intensity (Biazus-Sehn et al., 2020) and a moderate training volume (60 – 120 min/week) (Wu et al., 2019) have been shown to be the most effective to improve cognitive functioning. When complementing findings for motor-cognitive training in HOAs, higher trainings frequencies [≥3x/week (Chen et al., 2020), ≥5x/week (Northey et al., 2018)], higher challenging motor tasks (Sherrington et al., 2017), shorter session durations (Mansor et al., 2020), and either longer (≥12 weeks) (Stanmore et al., 2017) or shorter (≤12 weeks) (Biazus-Sehn et al., 2020; Chen et al., 2020; Gavelin et al., 2021) intervention durations have been shown to improve effectiveness of motor-cognitive training interventions. However, these conclusions are opposed by other meta-analyses (Biazus-Sehn et al., 2020; Chen et al., 2020; Mansor et al., 2020). In older adults with mNCD, higher training frequencies have been shown to improve effectiveness of physical- (i.e., ≥4x/week) (Sanders et al., 2019) and cognitive training (i.e., > 3x/week) (Bahar-Fuchs et al., 2019), while shorter session durations (i.e., ≤ 30 min) (Sanders et al., 2019) of physical exercise and longer intervention durations of cognitive training interventions (i.e., ≥ 3 months) (Bahar-Fuchs et al., 2019) have been shown to exert more pronounced training effects. When considering the cognitive demands (e.g., task complexity) of the training intervention, no difference between simple and complex cognitive games have been found for cognitive training interventions in HOA (Toril et al., 2014) and the optimal cognitive load for motor-cognitive training remains unknown. There is also no evidence regarding the optimal progression, variation, or specificity of motor-cognitive training interventions. When considering findings for solely cognitive training, multi-domain training (Mewborn et al., 2017; Sherman et al., 2017; Bahar-Fuchs et al., 2019) including memory (Mewborn et al., 2017; Sherman et al., 2017) and working memory specific training (Mewborn et al., 2017) has been shown to be the most effective for improving cognition in HOA and older adults with mNCD, while the use of fewer games (≤6 games) (Toril et al., 2014) tends to be beneficial for HOA.

Taken together, the meta-analytically synthesized evidence suggests that an exergame-based motor-cognitive training intervention with a high training frequency (i.e., ≥5x/week), shorter session durations (i.e., ≤ 30 min), longer intervention durations (i.e., ≥ 12 weeks) and a moderate training volume (60 – 120 min/week) predicts the largest effects on cognition. The physical part of the training should focus on aerobic activities at moderate intensities performed in a vertical body position with body loading, whereas the cognitive challenges should include multicomponent demands including working memory and memory-specific training. The optimal level of cognitive demand remains to be established. Likewise, the adaptation of the intervention over time (i.e., variability, progression, periodization) remains to be determined, but preferably, both are adapted to the individuals’ abilities.

Herold et al. (2019a) proposed an adapted exercise prescription that could be used for monitoring the cognitive task demands as well as the adaptation of the intervention over time. This adapted exercise prescription suggests that the exercise parameters are operationalized and adapted to the individual by tailoring external training loads (e.g., by manipulating exercise intensity) using specific markers of the internal training load to provide comparable inter-individual exercise doses (Herold et al., 2019a). The internal training load can be described as acute individual response [i.e., biomechanical, physiological, and/or psychological response(s)] to training components (e.g., external training load) and other influencing factors (e.g., climatic conditions, equipment, ground condition) (Impellizzeri et al., 2019). This adapted exercise prescription approach is believed allowing further insights into dose-response relationships and to result in more distinct training effects (Herold et al., 2019a; Stojan and Voelcker-Rehage, 2019). Fortunately, exergames are well suited for such individualized training concepts. In fact, individual real-time adaptivity of task demands according to monitored parameters such as performance, measures of brain activity, or internal training load is considered a key advantage of serious video games (such as exergames) (deBettencourt et al., 2015; Mishra et al., 2016; Sokolov et al., 2020), *“games that do not have entertainment, enjoyment or fun as their primary purpose”* (Lau et al., 2016). Therefore, developing an exergame-system in line with this adapted exercise prescription could be a key advantage for monitoring the cognitive task demands as well as the adaptation of the intervention over time. Additionally, variability of exergames can easily be applied for example by offering multiple exergames for the training of a specific neurocognitive function. Based on these findings, different evidence-based concepts and ideas for the design of the remaining exergame parameters (i.e., complexity, progression and periodization, and variability/variation) were synthesized (see Table 4 for an overview and Supplementary File 2 for a description of the suggested concepts):

Diamond and Ling (2016) hypothesized that games that combine physical activity with motor skill task learning through provision of complexity, novelty, and variety within the training context will be most effective for executive functions improvement. Regarding the monitoring of neurocognitive demands (i.e., game complexity), and in line with the adapted exercise prescription proposed by Herold et al. (2019a), using a biocybernetic adaptation loop (BIOLOOP) based on monitoring internal training load would most certainly be the optimal approach. In short, a *“biocybernetic loop is a modulation technique from the physiological computing field, which utilizes body signals in real-time to alter the system in order to assist users”* (Pope et al., 1995, 2014; Fairclough, 2015; Muñoz et al., 2018). *“This model of closed-loop control detects deviations from an optimal state of brain activity and uses these variations to cue changes at the human-computer interface in order to “pull” the psychological state of the user in a desired direction”* (Ewing et al., 2016). Optimally, it would work on basis of specific markers of internal training load to adapt the external training demands (Herold et al., 2019a). However, the optimal marker(s) for internal training load remain to be determined (Herold et al., 2019a). Alternatively, this adaptation loop could also be based on performance metrics of the exergame (e.g., speed, accuracy, reaction time), like described in the concept of the performance adaptation loop (PERF-LOOP). For the physical exercise intensity, the concept of monitoring target intensity (TARGETINT) is often used. In this concept, intensity is displayed in real-time by monitoring parameters of internal/external training load (e.g., heart rate). Participants have to change their behavior (e.g., increase stepping frequency) in order to reach the target intensity (Panza et al., 2018; Sanders et al., 2019). Optimally, these concepts would be applied concurrently, to ensure the optimal (i.e., moderate) predefined level of physical exercise intensity while adapting the neurocognitive demands (i.e., game complexity) to the individuals’ capabilities. This concept will be called BIOTARGETLOOP and will be introduced in more detail in section “Step 2: Development and Validation of Adaptation Loop.”

Regarding training progression, the concept of performance plateau (PLAT), in combination with dips and leaps may be used (Gray and Lindstedt, 2017). These are behavioral markers that relate to motor skill acquisition and can be analyzed with a focus on micro dynamics of individual performance curves (Gray and Lindstedt, 2017). In this concept, chosen games will be played and performance plateaus, dips and leaps are identified. The occurrence of the performance plateau (after several training sessions) will for example mark the introduction of a new (slightly more difficult) exergame. Future long-term brain training studies using long-term video game training interventions seems ideal for capturing detailed longitudinal data (Gray and Lindstedt, 2017), from which big data can be harvested and analyzed from gaming records.

Regarding the variability of exergames, the concept of MYCHOICE seems to be promising. It describes a self-determined choice of games within groups of games for neurocognitive domains. More specifically, in this concept, exergames will be grouped into the trained neurocognitive domains (e.g., learning and memory, executive function, complex attention, visuo-spatial skills) and each participant gets to choose which game within these groups he wants to play.

##### Integration of Chosen Training Parameters Into Requirements for a Training Concept

Based on the synthesized evidence an exergame-based motor-cognitive training intervention with a high training frequency (i.e., ≥5x/week), short session durations (i.e., ≤30 min), and a moderate training volume (60 – 120 min/week) applied over a duration of at least 12 weeks predicts the largest effects on cognition. The physical part of the training should focus on aerobic activities at moderate intensities, whereas the cognitive challenges should include multicomponent demands including skill-learning elements, working memory, and memory-specific training. The optimal level of cognitive demands as well as the adaptation of the intervention over time (i.e., variability, progression, periodization) may be monitored and adapted by the exergame device integrating the concepts of BIOTARGETLOOP, PLAT, and MYCHOICE.

The findings of our qualitative study suggested the use of exergames as a form of coupled motor-cognitive training that should be prescribed domain-specific depending on a patients’ cognitive abilities. The recommended training frequency ranged between two to five or more training sessions per week, largely dependent on training location and motivation. Training at home was reported to be preferred, since it represents a known environment which makes patients feel more secure and to enable a higher training frequency. However, multiple factors need to be considered to make a home-based training intervention feasible, like the improvement of game instructions, accessibility of a handrail or similar for mobility support, avoidance of technical problems, and the integration of a guided familiarization period or support of a carer to make the transfer to home-based exergaming easier. The recommended session durations should range between a minimum of 15 – 20 min up to a maximum of 30 min with the aim to reach a moderate training volume of approximately 150 min/week. Shorter sessions and a higher training frequency were reported to be preferable to reach this training volume mainly due to attentional exhaustion. The physical exercise intensity should be maintained at a light to moderate level, while the focus should be on game complexity that should be challenging but feasible. Individualization of the exergame-based training concept should mainly account for two aspects: (1) task type (i.e., choice of exergames to individually focus on neurocognitive functioning), and (2) task demands (i.e., adapt the game demands according to the individual capabilities to maintain a challenging but feasible cognitive training load). The task demands can be varied on multiple levels, for example: (1) stability support (use of handrail with both hands, one hand, or no support), (2) stepping direction, (3) game choice and tasks included, (4) game duration, or (5) game speed. To maintain the training program in the long-term (preferably > 12 weeks), motivation is a key factor and should be facilitated by the playful character of the exergames as well as a variation in the choice of games (Manser et al., 2022a) (see text footnote 1).

Based on the MIDE framework-based considerations so far, requirements for the optimal training components based on the findings of the qualitative study as well as the synthesized evidence were summarized (Table 5). As can be seen in Table 5, most of the optimal evidence-based training parameters are in line with the recommendations of experts and the preferences of patients as indicated by the results of the qualitative study. Based on the integration of these findings, the following components for a theoretically optimal training intervention concept were determined: The training should consist of an individually adapted multi-domain exergame-based simultaneous motor-cognitive training with incorporated cognitive tasks adopted with a deficit-oriented focus. A high training frequency (i.e., ≥ 5x/week), short session durations (i.e., ≤ 30 min), and a moderate training volume (60–120 min/week) should be applied over a duration of at least 12 weeks. The exergame demands should be individually adapted to maintain a moderate physical exercise intensity and a challenging but feasible neurocognitive demand.

To be able to apply a theoretically optimal training intervention concept, the following exergaming technology requirements are to be considered (see “Step 4: Technology Scoping”). In this phase of the project, we determined the hardware and software requirements for developing and deploying the exergames (Li et al., 2020).

#### Step 4: Technology Scoping

A previous study showed good results in people with major neurocognitive disorders using a Dividat Senso platform (Swinnen et al., 2021). We will use this device and can thus use some of the existing exergames by adapting these to the determined requirements for our future studies. As described in section “Treatment Preferences” and “Motivators for Treatment,” the use of exergames (i.e., Dividat Senso) was positive, especially because of the simple and clear design structures of the games that were highly appreciated by patients and that were comprehensible for the training tasks. An additional motivation for the use of exergames are the feelings of excitement, enjoyment or fun that is maintained by the inclusive character of exergames as previously reported (Swinnen et al., 2020a). Therefore, only minor modifications of the exergame device and game scenarios are required. These required modifications were synthesized in our qualitative study and mainly covered adjustments in game complexity at the start of the game (i.e., widening the opportunities to adjust task difficulty downwards) and several minor game-specific adaptations. Finally, the technological requirements to meet the requirements of training parameters for the project are summarized in Table 3. As can be seen, the usability of the home-based version (Dividat Senso Flex) needs to be tested, and additional studies as well as the expertise of the development team of Dividat AG will be required to integrate novel game designs or -elements (i.e., development, validation and integration of novel/adjusted adaptation loop, identification of performance plateau).

**TABLE 3:** Hardware and software requirements of the Dividat Senso for Brain-IT.

**Training parameter**	**Requirements for intervention concept**	**Technological requirements**	
		**Requirements met?**	**Necessary advancements:**
Frequency	High frequency (≥ 5x/week)	Partially	• Usability of the Dividat Senso Flex
Intensity/Complexity	Real-time closed-loop adaptation of exergame demands to internal training load (BIOTARGETLOOP)	No	• Development and Validation of Adaptation Loop
							• Integration of Adaptation Loop into Software
Type (of training)	Exergame-based simultaneous incorporated motor-cognitive training	Yes	
Time (exercise duration)	<30 min	Yes	
Duration (of intervention)	12 weeks	Yes	
Volume	Moderate (60 – 120 min/week)	Yes	
Progression and Periodization	Adaptation based on performance plateau according to predefined taxonomy	Partially	• Identification of Performance Plateau by Software
Variability/Variation	Self-determined choice	Yes	
Specificity	Individualized focus in a multi-domain training including working memory, memory + flexibility tasks	Partially	• Development of new Games (i.e., Episodic Memory, Working Memory)
			

**TABLE 4:** Possible ideas/concepts for training monitoring.

**Training parameter**	**Type of exercise**			**Preferred choice for Brain-IT**
	**Cognitive exercises**	**Physical exercises**	**Motor-cognitive exercises**	
Complexity	BIOLOOP (=Biocybernetic adaptation loop) PERF-LOOP (=Performance adaptation loop)	TARGETINT (=Monitoring of target intensity)	BIOLOOP (=Biocybernetic adaptation loop) PERF-LOOP (=Performance adaptation loop)	BIOTARGETLOOP
Progression and Periodization	PLAT (=Performance Plateau)	ADAPT (=Adaptation of intensity according to training progress) HRV-GUIDE (=HRV guided exercise prescription)	PLAT (=Performance Plateau)	PLAT
Variability/Variation	MYCHOICE (=Self-determined choice of games within groups of games for cognitive domains)		MYCHOICE (=Self-determined choice of games within groups of games for cognitive domains)	MYCHOICE

**TABLE 5:** Overview of preferred training parameters and final decision for Brain-IT.

**Exercise and training parameters**	**Preferences based on:**			**Requirements for a theoretically optimal training intervention concept**
	**Meta-analytic results**	**Additional evidence**	**Qualitative study**	
Frequency	High frequency (≥5x/week)		High frequency (≥5x/week), but only if home-based training is possible	High frequency (≥5x/week)
Intensity/Complexity	Physical load: moderate intensity motor complexity: high challenge cognitive load: unknown	Real-time closed-loop adaptation of exergame demands to internal training load (BIOTARGETLOOP)	Physical load: moderate intensity cognitive load: challenging but feasible	Real-time closed-loop adaptation of exergame demands to internal training load (BIOTARGETLOOP)
Type (of training)	Individually applied simultaneous motor-cognitive training		Exergaming	Exergame-based simultaneous incorporated motor-cognitive training
Time (exercise duration)	≤30 min		<30 min	≤30 min
Duration (of intervention)	≥12 weeks		Long-term	≥12 weeks
Volume	Moderate (60 – 120 min/week)		Moderate (60 – 120 min/week) to high	Moderate (60 – 120 min/week)
Progression and Periodization	Unclear	Adaptation based on performance plateau according to predefined taxonomy	Unclear	Adaptation based on performance plateau according to predefined taxonomy
Variability/Variation	Unclear	Self-determined choice	Use a certain routine with slight variations over time	Self-determined choice
Specificity	Focus on working memory and memory training as part of a multi-domain training	Multi-domain training including working memory, memory + flexibility tasks	Focus on cognitive deficits	Individualized (deficit-oriented) focus in a multi-domain training including working memory, memory training

#### Step 5: Sustainability Strategy

The goal of this step was to *“consider strategies to be distributed/maintained outside of the research period so that they are available more widely and for longer-term by end-users and healthcare institutions“* (Li et al., 2020).

The exergame training system we intend to use (Dividat Senso) is CE-marked as a medical device and available at more than 150 places (i.e., mainly senior residences, rehabilitation clinics and physiotherapies) in Switzerland. Additionally, a home-based telerehabilitation version (Dividat Senso Flex) is currently developed and expected to be accessible soon. Therefore, availability of the training system is ensured and is expected to be further improved (by accessibility of the Dividat Senso Flex) in the near future.

### Phase 2: Game Design and Development

The overall goal of phase 2 was to develop a fully functional prototype supported by multidisciplinary teamwork including the exergaming industry, game designers, clinical experts, researchers, and, of course, the end user (Li et al., 2020). First, the required adaptations [game design (see section “Step 1: Game Design”), development and validation of the adaptation loop (see section “Step 2: Development and Validation of Adaptation Loop”)] were addressed before proposing the novel exergame-based training concept (see section “Step 3: Development of Exergame-based Training Concept”). In a next phase, this exergame-based training concept is currently being tested on its feasibility, usability, and acceptance (see section “Step 4: Playtesting of Exergame-based Training Concept”). Based on the finding of the evaluation of feasibility, usability and acceptance, the training concept will then be modified (see section “Step 5: Modification of Exergame-based Training Concept”) and will finally enter Phase 3 (see section “Phase 3: System Evaluation”) for the final evaluation of effectiveness.

#### Step 1: Game Design

The MIDE framework requires several considerations regarding the game design. In this section we will reflect on these considerations and propose our solutions. Our goals in the first step of game design were *“to better understand the goal of the exergames and related training programs”* (Li et al., 2020), and *“to establish a mutual exergame design expectation”* (Li et al., 2020).

For the existing games, several game-specific adaptations were reported to be required. They mainly included adaptations in monitoring task demands as well as the game designs (Manser et al., 2022a) (see text footnote 1). These changes were implemented upon request by the development team of Dividat AG. In addition to these game-specific adaptations, multiple novel game designs or -elements were suggested and discussed by the focus groups in our qualitative study to optimally address patients’ needs. In general, it was recognized that there is a need for new games specifically targeting the neurocognitive functions of (motor) learning and memory as well as executive functions (i.e., working memory, cognitive inhibition) in general (Manser et al., 2022a) (see text footnote 1).

When designing novel exergames for older adults with mNCD, specific criteria were reported to be central in our qualitative study. In general, the games should use simple graphics and ensure good contrast. A good level of comfort with and good usability of the exergames need to be ensured by using easily comprehensible and clearly designed tasks with a certain closeness to everyday life. Multimodal animations including multisensory feedback should additionally be integrated focusing on positive reinforcement mechanisms to motivate patients during exergaming. Additionally, it is important that the main task is in the center of the screen and that only elements that are related to the game task are included. Moreover, too confronting performance feedback and unexpectedly appearing items or technical problems should be avoided (Manser et al., 2022a) (see text footnote 1).

Based on these findings and criteria for the game designs, multiple games were designed and submitted to Dividat AG for future training interventions. The suggested new games included a total of nine game suggestions in the neurocognitive domain of (motor) learning and memory, four game suggestions in the neurocognitive domain of executive functioning, and one game suggestion in the neurocognitive domain of visuo-spatial skills. Additionally, a new game mode was designed and submitted to Dividat AG that is based on HRV biofeedback and cardiac coherence training with the aim to be used as a behavioral intervention in order to improve the dynamic balance of the autonomic nervous system (ANS) and to regulate emotional state (Lehrer and Gevirtz, 2014). Of all these suggestions, four games in the neurocognitive domain of learning and memory as well as the new game mode for cardiac coherence training were implemented by Dividat AG to be used in our project. The specific game design and tasks are illustrated and explained in Supplementary File 3 in detail.

#### Step 2: Development and Validation of Adaptation Loop

As discussed in section “Quantitative Training Components,” instant adaptability is considered a key advantage of exergames, while the concept of BIOTARGETLOOP based on marker(s) of internal training load would be the optimal to ensure the optimal (i.e., moderate) predefined level of physical exercise intensity while adapting the neurocognitive demands (i.e., game complexity) to the individuals’ capabilities. This concept will now be introduced in more detail.

**FIGURE 1:**
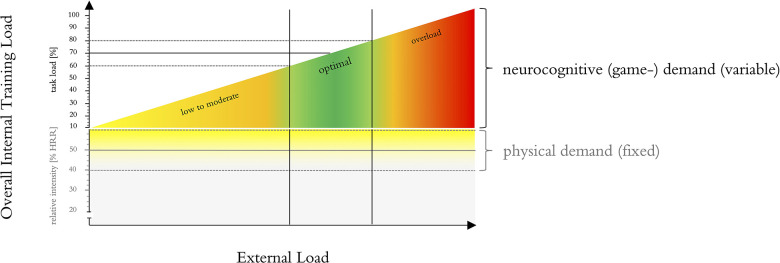
Methodological framework for the contribution of physical- and neurocognitive- (i.e., game-) demands during exergaming.

It is known that during motor-cognitive training (e.g., exergaming), the external task demands are mainly dependent on neurocognitive task demands and the physical exercise intensity (Netz, 2019). Comprehensive guidelines and checklists are available that provide classifications of exercise intensities and -doses for numerous parameters (e.g., percentage of individual maximal heart rate) (Halson, 2014; Hoffman, 2014; Slade et al., 2016; American College of Sports Medicine et al., 2017; Herold et al., 2019a). According to the American College of Sports Medicine, relative aerobic exercise intensities ranging between 40 and 59% heart rate reserve (HRR), 64 and 76% of maximal heart rate (HR_*max*_), or 45 and 67% of maximal oxygen uptake (VO_2__, max_) are considered moderate (Garber et al., 2011). Therefore, objective monitoring of the relative physical exercise intensity is readily applicable, although these methods are not without limitations. All these methods are based on prescribing exercise intensity relative to maximal anchors, which have been reported to result in an indistinct and heterogeneous homeostatic perturbation (Jamnick et al., 2020). Nonetheless, *“studies involving only moderate exercise intensity (e.g., B60% VO2max) might reasonably choose %VO2max, %HRmax, %VO2R, or %HRR over threshold-based relative exercise intensity prescription”* (Mann et al., 2013). For the neurocognitive demand – that serves as the driving mechanisms for task-specific neuroplasticity (Netz, 2019) – the optimal internal training load remains to be established. Using specific markers to quantify the neurocognitive demand would be advantageous, since an adequate dose acts as an essential factor for triggering neurobiological processes (Herold et al., 2019a). To be able to differentiate between the physical- and neurocognitive demands during exergaming, a theoretical model was proposed (Figure 1). In this model, the total individual internal training load is subdivided into a fixed component (i.e., physical exercise intensity) and a variable component (i.e., game demand). The fixed component comprises the relative exercise intensity that is independent of the game demands. It will be individually determined, set to a moderate level [i.e., 40–59% heart rate reserve (HRR)], and held constant over the course of the exergaming intervention. On top of this fixed physical exercise intensity, a variable amount of external training load will be presented that is regulated on basis of the game demands (e.g., game type, task complexity, predictability of required tasks). Since the physical exercise intensity is kept constant, changes in the overall internal training load can mainly be attributed to these game demands and, accordingly, the internal training load can be adjusted on basis of these game characteristics. This allows an individualized adaptation of the external training load according to the internal training load and will serve as a basis for the evaluation of the progression algorithm.

Optimally, such an algorithm would work on basis of specific markers of internal training load to adapt the external training demands (Herold et al., 2019a). Currently, the exergame training system Dividat Senso offers the concept of a PERF-LOOP (performance adaptation loop; as discussed in section “Quantitative Training Components”). The progression algorithm is based on performance indicators such as reaction times or point rate. However, the underlying progression algorithm was not yet formally validated or experimentally investigated. Additionally, the optimal marker(s) for internal training load remains to be discovered (Herold et al., 2019a). Therefore, we have conducted and are currently writing the manuscript of an experimental study with the aim to explore novel strategies for a real-time adaptive exergame system to individually tailor exergame demands according to the users’ physical and/or cognitive capabilities. More precisely, based on our findings in a recently published systematic review, the reactivity of vagally mediated heart rate variability (HRV) is evaluated as a promising monitoring parameter for internal training load that is easily measurable (Manser et al., 2021). Based on the findings of this study (Manser et al., 2022c^2^), the monitoring strategy for the final training concept was set and possible future advances for monitoring and adapting the external training load characteristics to ensure optimal internal training load were explored. However, these possible future advances remain explorative due to the constraints in time and resources within this project and may be further investigated at a later timepoint.

#### Step 3: Development of Exergame-Based Training Concept

Based on the MIDE framework-based considerations so far, we developed an exergame-based training concept that will be described in the following sections together with a provision of the development rationale. To increase the probability that the resulting training concept will be deemed feasible in future clinical practice, we used our considerations to guide the decision process of the theoretically optimal intervention design. The final training concept was developed on basis of the requirements for the optimal training components summarized in Table 5 that were defined based on the findings of the qualitative study as well as the synthesized evidence. Based on the integration of these findings, the following components of the training concept were determined, that were planned and will be reported using the Consensus on Exercise Reporting Template (CERT) (Slade et al., 2016) [for more detail, consider Supplementary File 3 which contains our complete exergame-based concept with sufficient details about the exergame components as well as the exercise and training characteristics (i.e. including all predefined levels of task demands as well as the detailed progression rules) to allow full replication].

##### Overview

The final training concept consists of an individually adapted multi-domain exergame-based simultaneous motor-cognitive training with incorporated cognitive tasks that will be adopted with a deficit-oriented focus on the neurocognitive domains of (1) learning and memory, (2) executive functioning, (3) complex attention, and (4) visuo-spatial skills. According to the training concept, each participant is instructed to train at least 5x/week for 21 min per session resulting in a weekly training volume of ≥105 min. All training sessions are planned to take place at participant’s homes using the exergame training system Dividat Senso Flex.

**FIGURE 2:**
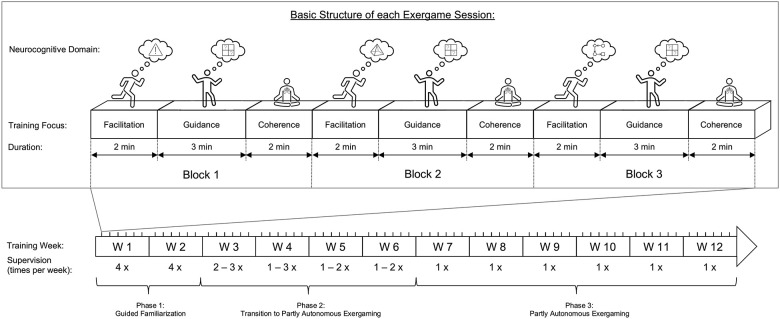
Overview of the exergame-based intervention concept and the basic structure of each exergame session (here as an example for a patient with amnestic-single domain mNCD with a training focus on learning and memory in week 1).

The training concept is structured in three phases. It starts with a familiarization period of two weeks. During this phase, most of the training sessions (i.e., 4 out of 5 sessions) are supervised. After this initial guided familiarization period, supervision of training sessions is gradually reduced to 1x/week during a four-week transition phase. This transition phase aims to lead participants to being able to train independently while being remotely monitored. In this transition phase, the amount of supervision of training sessions is individually determined within a predefined range (see Figure 2) in accordance with the capabilities and preferences of the participants. From the 7th week until completion of the training intervention, semi-autonomous training with one supervised training session per week is prescribed for each participant.

##### Structure of Each Exergame Session

Throughout the training intervention period, all sessions will be prescribed following the same basic structure: Each session consists of three blocks with three phases per block (see Figure 2).

*Phase 1.* Facilitation aims to apply a moderate physical exercise intensity in the context of challenging but feasible cognitive and motoric demands mainly intending to *“trigger neurophysiological mechanisms, which promote neuroplasticity”* (Fissler et al., 2013; Herold et al., 2018) while additionally using *“cognitive stimulation [*…*] to “guide” these neuroplastic processes”* (Fissler et al., 2013; Bamidis et al., 2014; Herold et al., 2018). This phase includes games focusing on neurocognitive domains that are least impaired. The external task demand is individually adapted to ensure an appropriate internal training load. More specifically, the internal training load is subdivided into a fixed component (i.e., physical exercise intensity) and a variable component [i.e., neurocognitive (game-) demand]. An additional stepping task is used to set the level of physical exercise intensity. It includes walking on the spot at a predefined stepping frequency that is needed to reach a moderate level of physical exercise intensity [i.e., ranging between 40 and 59% heart rate reserve (HRR) (Garber et al., 2011)]. The stepping frequency will be individually determined for each participant (see section “Phase 1 – Facilitation”). A battery figure add-on is visible in the center of the screen that provides real-time visual feedback whether the predefined stepping frequency is reached. More specifically, if the predefined minimal required stepping frequency is reached or exceeded, the battery stays at equilibrium or fills. If the battery level is above 80% (indicated by a line), the battery stays green. If the participants’ stepping frequency falls below the predefined minimal required stepping frequency, the battery level decreases, and the battery turns orange (40 – 80%) or red (below 40%) indicating that the stepping frequency should be increased. On top of this fixed physical exercise intensity, a variable amount of neurocognitive (game-) demands (e.g., game type, task complexity, predictability of required tasks) is applied. Since the physical exercise intensity is kept constant, changes in the overall internal training load can mainly be attributed to these neurocognitive and motoric (game-) demands and, accordingly, the internal training load can be adjusted on basis of these game characteristics according to predefined progression rules (see section “Progression Rules for Monitoring Internal Training Load and Adapting External Training Loads”).

*Phase 2.* Guidance aims to make use of the triggered neurophysiological mechanisms from phase 1 to specifically guide neuroplastic processes of the mainly impaired neurocognitive domain. Therefore, games focusing on the mainly impaired neurocognitive domain for the individual participant (e.g., amnestic single domain → learning and memory) are used. These games solely focus on cognitive and motoric demands, but not on physical exercise intensity. The cognitive-motoric demands of the exergame are individually adapted to ensure an appropriate total internal training load according to predefined progression rules (see section “Progression Rules for Monitoring Internal Training Load and Adapting External Training Loads”).

*Phase 3.* Coherence aims to implement a structured approach as a surrogate for the breaks between games. Patients with mNCD often exhibit depressive symptoms and anxiety, which are in turn important indicators for progression to dementia (Ismail et al., 2017; Ma, 2020). To account for these psychological factors, resonance breathing guided by heart rate variability biofeedback (HRVB) will be used. HRVB training is a behavioral intervention aiming to increase cardiac autonomic control, to enhance homeostatic regulation, and to regulate emotional state (Lehrer and Gevirtz, 2014; Shaffer et al., 2014). It consists of a regular breathing practice at a specific frequency that is individually determined that produces high amplitude of HRV. Usually, this resonance breathing frequency is around 6 breaths/min (Schwerdtfeger et al., 2020). An increased HRV is predicted to increase vagal afferent transmission to the forebrain, activate the prefrontal cortex, and improve executive function (Shaffer et al., 2014). In fact, multiple systematic reviews and meta-analyses have indicated that HRVB training or paced breathing (at resonance frequency) is effective in decreasing depressive symptoms and anxiety in healthy adults and also clinical populations. Additionally, improved sleep quality, quality of life, HRV and brain activity in regions relevant for cognitive adaptations have been reported (Goessl et al., 2017; Zaccaro et al., 2018; Lehrer et al., 2020). The evidence for older adults (i.e., ≥ 60 years) or patients with cognitive impairments is sparse, but decreases in depression, anxiety, and increases in attentional performance (no sign. difference in executive functioning) have already been reported, suggesting that older adults may benefit from HRVBT much like the younger populations (Jester et al., 2019). Additionally, *“after initial training some people still achieve better results by following a heart monitor, while others do just as well doing paced breathing at their resonance frequency, once this frequency has been determined by biofeedback, following the second hand on a clock or counting seconds silently“* (Lehrer et al., 2020). Therefore, for the sake of simplicity, we will make use of this transfer to resonance breathing. Before starting the training intervention, the resonance frequency is determined according to the protocol of Lehrer et al. (2013) (i.e., visit 1 of their protocol). During the training intervention, coherence breathing includes paced breathing for 2 min following the rhythm of the individually predetermined resonance frequency visualized on the screen of the exergame device (i.e., a sun is displayed within a landscape. When the sun gets bigger, the patients breath in. When the sun gets smaller, the patients breath out).

##### Progression Rules for Monitoring Internal Training Load and Adapting External Training Loads

*Phase 1 – Facilitation.* As described above, the internal training load will be subdivided into the physical exercise intensity of the stepping task and the neurocognitive and motoric (game-) demands of the games in phase 1. The stepping frequency of the stepping tasks will be predetermined for each participant with the aim to reach a moderate level of physical exercise intensity [i.e., ranging between 40 and 59% HRR (Garber et al., 2011)]. To avoid overload, the participants will be introduced stepwise; first, all the stepping frequency will be determined while the level of neurocognitive demand is held at level 1. Afterward, the total level of internal training load will be monitored and adapted.

Phase 1a – Determination of minimal stepping frequency:

All participants will start with a stepping frequency of 100 steps/min and at Level 1 of neurocognitive demands in the first training session. The target physical exercise intensity is determined based on the target heart rate (HR) that is calculated using the Karvonen method with a target intensity of 40% HRR: HR_target_ = (HR_max_ – HR_rest_) ⋅ 0.40 + HR_rest_ (Karvonen et al., 1957; Karvonen and Vuorimaa, 1988). For this calculation the age-predicted maximal heart rate: HR_*max*_ = 208 – 0.7 ⋅ age and HR_*rest*_ measured at the pre-measurements will be used. The stepping frequency will then be increased by 5 steps/min at each training session, until the minimal level of physical exercise intensity is reached, but to a maximal level of 140 steps/min. The evaluated stepping frequency will then be considered as a fixed component of the overall external training load. In all subsequent training sessions, this fixed physical exercise intensity will be kept constant and the focus shifts to monitoring and adapting the total internal training load.

Phase 1b – Monitoring and adaptation of total internal training load:

Since the physical exercise intensity in phase 2 is kept constant, changes in the overall internal training load can mainly be attributed to the variable level of neurocognitive demand. The level of neurocognitive demand will be standardized according to predefined game levels. Phase 2 will be continued with game level 1, until a plateau in performance is reached. Unfortunately, reading out a plateau of performance by the software of the exergame training system is not (yet) implemented. Therefore, a plateau in performance will be read out visually guided by the following predefined criteria: (1) a performance increase of less than or equal to 5% compared to the previous exergame session while (2) there was an increase in performance from session to session over at least the previous three training sessions. Each time a plateau in performance is reached, the game level will be increased by one level or a new (slightly more difficult) exergame will be introduced.

*Phase 2 – Guidance.* In phase 2, the mainly impaired neurocognitive domain will be trained. Therefore, the focus of monitoring and adapting the task demands will solely focus on neurocognitive demands (i.e., motor- and cognitive demands that are linked because both change as a function of game complexity). The level of neurocognitive demand will be standardized according to predefined game levels. All participants will start with level 1. Each time a plateau in performance is reached, the game level will be increased by one level or a new (slightly more difficult) exergame will be introduced.

##### The Concept of MYCHOICE to Ensure Sufficient Variability

The concept of MYCHOICE describes a self-determined choice of exergames within groups of games for cognitive domains so that the preferences of each participant can be taken into account while the time spent at training each neurocognitive domain is still standardized within participants with the same training focus (i.e., predetermined according to the deficit-oriented focus on the neurocognitive domains). The advantage of this concept is that it promotes self-efficacy, which might have a positive influence on training motivation (Di Lorito et al., 2019). According to the ‘Optimizing Performance through Intrinsic Motivation and Attention for Learning (OPTIMAL)’ theory of motor learning (Wulf and Lewthwaite, 2016), this is expected to enhance performance expectancies which – accompanied with these autonomy-supportive conditions – *“contribute to efficient goal-action coupling by preparing the motor system for task execution”* (Wulf and Lewthwaite, 2016). This is further proposed *“to facilitate the development of functional connectivity across brain regions, and structural neural connections more locally, that support effective and efficient motor performance and learning”* (Wulf and Lewthwaite, 2016; Lemos et al., 2017). With this regard, the exergames were grouped into mainly trained neurocognitive domains of learning and memory, executive function, complex attention, visuo-spatial skills (see Supplementary Table S1 in Supplementary File 3) and each participant gets to choose which game within these groups he/she prefers to play.

#### Step 4: Playtesting of Exergame-Based Training Concept

Goal: *“Through multiple playtesting and informal feedback sessions, specific game preferences and game elements will be modified based on the feedback from older adults and healthcare professionals during their one-on-one interactions with the prototype”* (Li et al., 2020).

The resulting training concept is currently being tested on its feasibility, usability, and acceptance. With this regard, a two-arm, parallel-group, single-blinded (i.e., outcome evaluator of pre- and post-measurements blinded to group allocation) pilot randomized controlled trial (RCT) with an allocation ration of 2:1 (i.e., intervention:control) including 17 – 25 older adults with mNCD is conducted. In this study, the active control group proceeds with usual care as provided by the (memory) clinics where the patients are recruited. The intervention group performs a 12-week training intervention according to the newly developed exergame-based training concept in addition to usual care. The primary outcomes include feasibility (i.e., recruitment, adherence, compliance, attrition), usability (i.e., system usability), and acceptance (i.e., enjoyment, training motivation and perceived usefulness) of the resulting exergame-based training concept for older adults with mNCD. As a secondary objective, preliminary effects of the intervention on cognition, brain resting-state functional connectivity, gait, cardiac autonomic regulation, and psychosocial factors (i.e., quality of life, and levels of depression, anxiety, and stress) are explored. This will allow to synthesize data for a sample size calculation on basis of a formal power calculation for a future RCT. The study was registered at clinicaltrials.gov (NCT04996654) and will be reported according to the “The Consolidated Standards of Reporting Trials (CONSORT) 2010 statement: extension to randomized pilot and feasibility trials” (Eldridge et al., 2016; Manser et al., 2022b^3^).

#### Step 5: Modification of Exergame-Based Training Concept

The MIDE framework also requires a system evaluation in phase 3. Based on the results of our pilot RCT (Manser et al., 2022b) (see text footnote 3), the intervention concept will be modified for its final evaluation on effectiveness with expected contributions from end users, clinicians, researchers, and data analysts.

### Phase 3: System Evaluation

Goal: To systematically evaluate the exergame system *“to ensure the exergames meet their intended goals”* (Li et al., 2020) regarding therapeutic outcomes, user experience, and technology performance (Li et al., 2020).

In the final phase, we will aim to evaluate the effectiveness of the newly developed exergame-based training intervention in older adults with mNCD with respect to cognition, brain structure and function and quality of life. We will strive to recruit n (depending on an *a priori* sample size calculation) participants that will be randomly assigned to either the intervention group (i.e., exergame intervention) or the control group (i.e., usual care). The primary outcome will include global cognition assessed with the Quick Mild Cognitive Impairment Screen (Qmci) (O’Caoimh, 2015). As secondary outcomes, domain-specific assessments for the evaluation of the key neurocognitive domains [as defined by Sachdev et al. (2014) in line with DSM-V (American Psychiatric Association, 2013)] of learning and memory, complex attention, executive function, and visuo-spatial skills will be incorporated as recommended (Janelidze and Botchorishvili, 2018). Moreover, brain structure and function will be evaluated by magnetic resonance imaging with the aim to investigate more closely the underlying neural changes responsible for adaptations in cognitive performance. Gait, HRV (and its associations to neurobiological and cognitive changes), and psychosocial factors (i.e., quality of life and levels of depression, anxiety, and stress) will also be assessed. This study will be registered in https://clinicaltrials.gov and the study protocol will be published beforehand.

## Discussion and Conclusion

In this manuscript, the design and development process of novel exergame-based training concepts was illustrated using a step-by-step application of the MIDE-framework. The aim was to elucidate the design, development, and evaluation process of an exergame-based training concept to halt and/or reduce cognitive decline and improve quality of life in older adults with mNCD (Li et al., 2020).

The development of novel exergame-based training concepts for older adults with mNCD is greatly facilitated when it is based on a theoretical framework (e.g., the MIDE-framework). Applying this framework resulted in a structured, iterative, and evidence-based approach that led to the identification of multiple key requirements for the exergame design as well as the training components that otherwise may have been overlooked or neglected. This is expected to foster the usability and acceptance of the resulting exergame intervention in “real life” settings. Therefore, it is strongly recommended to implement a theoretical framework (e.g., the MIDE-framework) for future research projects in line with well-known checklists to improve completeness of reporting and replicability [i.e., CERT-checklist (Slade et al., 2016) in line with the CONSORT 2010 statement (Begg et al., 1996; Moher et al., 2010)] when serious games for motor-cognitive rehabilitation purposes are to be developed.

## Data Availability Statement

The original contributions presented in the study are included in the article/Supplementary Material, further inquiries can be directed to the corresponding author/s.

## Author Contributions

PM and EB were responsible for the conception, literature research, and writing of the manuscript. Both authors revised, read, and approved the submitted version.

## Conflict of Interest

The authors declare that the research was conducted in the absence of any commercial or financial relationships that could be construed as a potential conflict of interest.

## Publisher’s Note

All claims expressed in this article are solely those of the authors and do not necessarily represent those of their affiliated organizations, or those of the publisher, the editors and the reviewers. Any product that may be evaluated in this article, or claim that may be made by its manufacturer, is not guaranteed or endorsed by the publisher.

## Footnotes

1 Manser, P., Adcock, M., and de Bruin, E. D. (2022a). Design Considerations for an Exergame-based Training Intervention for Older Adults with mild Neurocognitive Disorder.

2 Manser, P., Adcock, M., and de Bruin, E. D. (2022c). Quantification of Individual Exergame Demands – Validity, and Reliability of vagally-mediated Heart Rate. (Variability)Reactivity to Measure Internal Training Load in Older Adults.

3 Manser, P., Adcock, M., and de Bruin, E. D. (2022b). Feasibility, Usability and Acceptance of a Newly Developed Exergame-Based Intervention Concept for Older Adults with Mild Neurocognitive Disorder – A Pilot Randomized Controlled Trial.
