# Supplementary material for: Making the Best Out of IT: Design and Development of Exergames for Older Adults With Mild Neurocognitive Disorder – A Methodological Paper
Source: Front Aging Neurosci. 2021 Dec 9;13:734012. doi: 10.3389/fnagi.2021.734012 (PMC8698204; doi:10.3389/fnagi.2021.734012)
Supplement: Supplementary file 1 [file Data_Sheet_1.pdf]

Supplementary Material

-

File 1 –Tables

Table S1: Summary of meta-analytic results for cognitive training interventions
color-coding: black = significant effect, grey = no significant effect
* = significant at p ≤ 0.05, ** = significant at p ≤ 0.01

Abbreviations: ADL = Activities of daily living, CI = confidence interval, g = Hedge's g, GRADE = Grading of Recommendations Assessment, Development and Evaluation, HOA = healthy older adults; k = number of studies; n = number of participants; NR = not reported, MD = mean difference (absolut), mNCD = mild neurocognitive disorder, SMD = standardized mean difference

Reference	Study Characteristics	Outcomes			
		Type	Statistics	Heterogeneity	GRADE
Bonnechère et al. 2020 [1]	POPULATION:<br>☒ healthy older adults<br>☐ older adults with mNCD<br>☐ older adults with Dementia	☒ Cognition			
		processing speed**	g^{+} = 0.40, p < 0.001, 95 % CI [0.20 to 0.60], k = 8, n = 403	☐ sign. ☒ n.s.	
		working memory**	g^{+} = 0.21, p = 0.001, 95 % CI [0.08 to 0.34], k = 9, n = 917	☒ sign. ☐ n.s.	
		processing speed**	g^{+} = 0.21, p = 0.006, 95 % CI [0.06 to 0.35], k = 9, n = 582	☐ sign. ☒ n.s.	
		verbal memory*	g^{+} = 0.12, p = 0.031, 95 % CI [0.01 to 0.24], k = 7, n = 907	☐ sign. ☒ n.s.	
		attention	g^{+} = 0.06, p = 0.59, 95% CI [-0.16 to 0.29], k = 4, n = 299	☒ sign. ☐ n.s.	
		visuospatial abilities	g^{+} = 0.03, p = 0.18, 95 % CI [-0.16 to 0.22], k = 4, n = 216	☐ sign. ☒ n.s.	
	INTERVENTION:<br>commercially available computerized videogames to perform cognitive training	☐ Brain / Neurochemicals / HRV			
		NR			
	COMPARISON:<br>mixed (i.e. active or passive brain training)	☐ Physical Outcomes, Mobility, and Activities of Daily Living			
		NR			
		☐ Psychosocial Outcomes			
		NR			
Chan et al. 2020 [2]	POPULATION:<br>☐ healthy older adults<br>☒ older adults with mNCD<br>☒ older adults with Dementia	☐ Cognition			
	INTERVENTION:<br>cognitive training intervention (including (i) computerized cognitive training, (ii) cognitive training and rehabilitation and (iii) cognitive stimulation therapy, such as reminiscence therapy and reality orientation.	☐ Brain / Neurochemicals / HRV			
		NR			
		☐ Physical Outcomes, Mobility, and Activities of Daily Living			
		NR			
	COMPARISON:<br>mixed (i.e. (i) the usual care (i.e. treatment as usual or waitlisted group); (ii) social and recreational activity; and (iii) alternative psychosocial intervention.	☒ Psychosocial Outcomes			
		depression**	SMD^{-} = - 0.54, p < 0.0001, 95 % CI [- 0.77 to - 0.31], k = 36, n = 2,414	☒ sign. ☐ n.s.	
Mansor et al. 2020 [3]	POPULATION:<br>☒ healthy older adults<br>☐ older adults with mNCD<br>☐ older adults with Dementia	☒ Cognition			
		updating memory*	g^{+} = 0.37, p = 0.02, 95 % CI [0.07 to 0.66], k = 23, n = 879	☒ sign. ☐ n.s.	
		inhibition*	g^{+} = 0.28, p = 0.02, 95 % CI [0.02 to 0.53], k = 20, n = 709	☒ sign. ☐ n.s.	
		reasoning	g^{+} = 0.17, p = 0.45, 95 % CI [- 0.28 to 0.62], k = 13, n = 523	☒ sign. ☐ n.s.	
		processing speed	g^{+} = 0.15, p = 0.20, 95 % CI [- 0.08 to 0.38], k = 21, n = 800	☒ sign. ☐ n.s.	
		shifting	g^{+} = 0.14, p = 0.46,95 % CI [- 0.21 to 0.50], k = 18, n = 693	☒ sign. ☐ n.s.	
		attention	g^{+} = 0.08, p = 0.77, 95 % CI [- 0.47 to 0.64], k = 11, n = 394	☒ sign. ☐ n.s.	
		delayed memory	g^{+} = - 0.03, p = 0.90, 95% CI [- 0.48 to 0.42], k = 10, n = 387	☒ sign. ☐ n.s.	
	INTERVENTION:<br>videogames (exergames, brain training, action, strategy, and casual games)	☐ Brain / Neurochemicals / HRV			
		NR			
	COMPARISON:<br>mixed (passive (i.e. no-contact; k = 24) or active (e.g. watched a movie or a documentary, read a book, completed quizzes, or exercised; k = 9))	☐ Physical Outcomes, Mobility, and Activities of Daily Living			
		NR			
		☐ Psychosocial Outcomes			
		NR			

Gates et al. 2020 [4]	POPULATION:<br>☒ healthy older adults<br>☐ older adults with mNCD<br>☐ older adults with Dementia<br><br>INTERVENTION:<br>computerized cognitive training for at least 12 weeks<br><br>COMPARISON:<br>(1) active control intervention (unguided computer- and/or screen-based tasks that are not a planned intervention. These tasks can involve watching educational videos or playing computer games, with no particular training component.)<br>(2) inactive control (no intervention)	☒ Cognition<br><br>speed of processing**(1) — SMD^- = - 0.63, p = 0.01, 95 % CI [- 1.14 to - 0.12], k = 2, n = 138 — ☐ sign. ☒ n.s. — very low<br>global cognition*(1) — SMD^- = - 0.31, p = 0.02, 95 % CI [- 0.57 to - 0.05], k = 2, n = 232 — ☐ sign. ☒ n.s. — low<br>working memory(1) — SMD^- = - 0.17, p = 0.08, 95 % CI [- 0.36 to 0.02], k = 3, n = 392 — ☐ sign. ☒ n.s. — low<br>executive function(1) — SMD^- = - 0.04, p = 0.89, 95 % CI [- 0.61 to 0.53], k = 3, n = 230 — ☒ sign. ☐ n.s. — very low<br>episodic memory(1) — SMD^- = 0.06, p = 0.57, 95 % CI [- 0.14 to 0.26], k = 4, n = 439 — ☐ sign. ☒ n.s. — low<br>episodic memory*(2) — SMD^- = - 0.90, p = 0.03, 95 % CI [- 1.73 to - 0.07], k = 1, n = 150 — N/A — low<br>speed of processing(2) — SMD^- = - 0.28, p = 0.31, 95 % CI [- 0.82 to 0.26], k = 2, n = 204 — ☐ sign. ☒ n.s. — very low<br>verbal fluency(2) — SMD^- = - 0.11, p = 0.88, 95 % CI [- 1.58 to 1.36], k = 1, n = 150 — N/A — low<br>executive function(2) — SMD^- = - 0.08, p = 0.51, 95 % CI [- 0.31 to 0.15], k = 2, n = 292 — ☐ sign. ☒ n.s. — low<br>working memory(2) — SMD^- = - 0.08, p = 0.66, 95 % CI [- 0.43 to 0.27], k = 1, n = 60 — N/A — low<br><br>☐ Brain / Neurochemicals / HRV<br><br>NR<br><br>☐ Physical Outcomes, Mobility, and Activities of Daily Living<br><br>NR<br><br>☐ Psychosocial Outcomes<br><br>NR
Bahar-Fuchs et al. 2019 [5]	POPULATION:<br>☐ healthy older adults<br>☒ older adults with mNCD<br>☐ older adults with Dementia<br><br>INTERVENTION:<br>cognitive training<br><br>COMPARISON:<br>mixed (i.e. "18 passive and 13 active control conditions, along with 15 alternative treatment conditions, including occupational therapy, mindfulness, reminiscence therapy, and others."[5])	☒ Cognition<br><br>delayed memory** — SMD^+ = 0.81, p = 0.002, 95 % CI [0.29 to 1.32], k = 11, n = 543 — ☒ sign. ☐ n.s. — very low<br>executive function** — SMD^+ = 0.75, p = 0, 95 % CI [0.28 to 1.22], k = 12, n = 511 — ☒ sign. ☐ n.s.<br>immediate memory** — SMD^+ = 0.74, p < 0.0001, 95 % CI [0.37 to 1.12], k = 18, n = 762 — ☒ sign. ☐ n.s.<br>language (naming)* — SMD^+ = 0.62, p = 0.02, 95 % CI [0.11 to 1.12], k = 5, n = 311 — ☒ sign. ☐ n.s.<br>attention & work. mem.* — SMD^+ = 0.56, p = 0.02, 95 % CI [0.56 to 1.05], k = 12, n = 551 — ☒ sign. ☐ n.s.<br>verb. category fluency** — SMD^+ = 0.52, p = 0, 95 % CI [0.23 to 0.81], k = 9, n = 475 — ☒ sign. ☐ n.s.<br>global cognition** — SMD^+ = 0.42, p < 0.0001, 95 % CI [0.23 to 0.62], k = 27, n = 1,389 — ☒ sign. ☐ n.s. — moderate<br>meta cognition — SMD^+ = 0.50, p = 0.13, 95 % CI [-0.15 to 1.14], k = 2, n = 41 — ☐ sign. ☒ n.s.<br>processing speed — SMD^+ = 0.22, p = 0.19, 95 % CI [-0.11 to 0.54], k = 6, n = 201 — ☒ sign. ☐ n.s.<br>verb. letter fluency — SMD^+ = 0.22, p = 0.13, 95 % CI [-0.07 to 0.50], k = 12, n = 544 — ☒ sign. ☐ n.s.<br><br>☐ Brain / Neurochemicals / HRV<br><br>NR<br><br>☒ Physical Outcomes, Mobility, and Activities of Daily Living<br><br>capacity for ADLs — SMD^+ = 0.12, p = 0.30, 95 % CI [- 0.11 to 0.35], k = 10, n = 687 — ☒ sign. ☐ n.s. — low<br><br>☒ Psychosocial Outcomes<br><br>participants' mood — SMD^+ = 0.72, p = 0.08, 95 % CI [- 0.1 to 1.54], k = 8, n = 577 — ☒ sign. ☐ n.s. — very low<br>neuropsych. symptoms — SMD^+ = 0.44, p = 0.27, 95 % CI [- 0.34 to 1.22], k = 6, n = 493 — ☒ sign. ☐ n.s.☒<br>quality of life — SMD^+ = - 0.04, p = 0.81, 95 % CI [- 0.38 to 0.29], k = 5, n = 630 — sign. ☐ n.s.
Gates et al. 2019 [6]	POPULATION:<br>☐ healthy older adults<br>☒ older adults with mNCD<br>☐ older adults with Dementia<br><br>INTERVENTION:<br>computerized cognitive training for at least 12 weeks<br><br>COMPARISON:<br>(1) active control intervention (unguided computer- and/or screen-based tasks that are not a planned intervention. These tasks can involve watching educational videos or playing computer games, with no particular training component.)<br>(2) inactive control (no intervention)	☒ Cognition<br><br>working memory* (1) — SMD^- = - 0.88, p = 0.04, 95 % CI [- 1.73 to - 0.03], k = 3, n = 72 — ☐ sign. ☒ n.s. — very low<br>episodic memory* (1) — SMD^- = - 0.79, p = 0.04, 95 % CI [- 1.54 to - 0.04], k = 5, n = 223 — ☒ sign. ☐ n.s. — very low<br>global cognition* (1) — SMD^- = - 0.53, p = 0.05, 95 % CI [- 1.06 to - 0.01], k = 5, n = 407 — ☒ sign. ☐ n.s. — very low<br>executive function (1) — SMD^- = - 0.31, p = 0.31, 95 % CI [- 0.90 to 0.28], k = 3, n = 150 — ☐ sign. ☒ n.s. — very low<br>verbal fluency (1) — SMD^- = - 0.16, p = 0.60, 95 % CI [- 0.76 to 0.44], k = 3, n = 150 — ☐ sign. ☒ n.s. — low<br>speed of processing (1) — SMD^- = 0.20, p = 0.28, 95 % CI [- 0.16 to 0.56], k = 2, n = 119 — ☐ sign. ☒ n.s. — low<br>episodic memory* (2) — MD^- = - 2.70, p = 0.02, 95 % CI [- 5.00 to - 0.40], k = 1, n = 37 — N/A — very low<br>executive function (2) — MD^- = - 2.70, p = 0.13, 95 % CI [- 6.21 to 0.81], k = 1, n = 37 — N/A — very low<br>global cognition (2) — MD^- = - 0.36, p = 0.29, 95 % CI [- 0.30 to 1.02], k = 1, n = 37 — N/A — very low<br>verbal fluency (2) — MD^- = 1.90, p = 0.56, 95 % CI [- 4.50 to 8.30], k = 1, n = 37 — N/A — very low<br><br>☐ Brain / Neurochemicals / HRV<br><br>NR<br><br>☒ Physical Outcomes, Mobility, and Activities of Daily Living<br><br>funct. performance (1) — SMD^- = 0.09, p = 0.76, 95 % CI [-0.51 to 0.70], k = 2, n = 131 — NR — NR<br>funct. performance (2) — MD^- = 0.00, p = 1, 95 % CI [-0.48 to 0.48], k = 1, n = 37 — N/A — very low<br><br>☒ Psychosocial Outcomes<br><br>depression (1) — SMD^- = -0.77, p = 0.24, 95 % CI [- 2.07 to 0.52], k = 3, n = 101 — ☒ sign. ☐ n.s. — low<br>quality of life (1) — MD^- = 0.40, p = 0.73, 95 % CI [- 1.85 to 2.65], k = 1, n = 19 — N/A — NR<br>depression* (2) — MD^- = -1.30, p = 0.05, 95 % CI [- 2.61 to 0.01], k = 1, n = 37 — N/A — NR

Vaportzis et al. 2019 [7]	POPULATION:<br>☒ healthy older adults<br>☐ older adults with mNCD<br>☐ older adults with Dementia<br><br>INTERVENTION:<br>video games, computer training, reading and arithmetic problem solving, and brain-computer interface<br><br>COMPARISON:<br>(1) active control intervention (e.g. stretching or balance classes)<br>(2) passive control (no intervention)	☒ Cognition<br>executive function (1) — SMD^+ = 0.63, p = 0.20, 95 % CI [- 0.33 to 1.59], k = 2, n = 123 — ☐ sign. ☒ n.s.<br>Stroop interference (1) — SMD^+ = - 0.12, p = 0.43, 95 % CI [- 0.41 to 0.17], k = 2, n = 268 — ☐ sign. ☒ n.s.<br>block design (2) — SMD^+ = 0.13, p = 0.57, 95 % CI [- 0.33 to 0.59], k = 2, n = 74 — ☐ sign. ☒ n.s.<br>digit substitution (2) — SMD^+ = 0.13, p = 0.58, 95 % CI [- 0.33 to 0.59], k = 2, n = 74 — ☐ sign. ☒ n.s.<br>digit span (2) — SMD^+ = 0.10, p = 0.67, 95 % CI [- 0.36 to 0.55], k = 2, n = 74 — ☐ sign. ☒ n.s.<br><br>☐ Brain / Neurochemicals / HRV<br>NR<br><br>☐ Physical Outcomes, Mobility, and Activities of Daily Living<br>NR<br><br>☐ Psychosocial Outcomes<br>NR
Zhang et al. 2019 [8]	POPULATION:<br>☐ healthy older adults<br>☒ older adults with mNCD<br>☐ older adults with Dementia<br><br>INTERVENTION:<br>computerized cognitive training<br><br>COMPARISON:<br>mixed (i.e. active (interventions that controlled for non-specific therapeutic effects) or passive (waiting list conditions, treatment as usual or a non-matched minimal intervention))	☒ Cognition<br>working memory** — g^+ = 0.39, p = 0.004, 95 % CI [0.12 to 0.66], k = 5, n = 165 — ☐ sign. ☒ n.s.<br>memory** — g^+ = 0.30, p = 0.002, 95 % CI [0.11 to 0.50], k = 13, n = 477 — ☒ sign. ☐ n.s.<br>global cognition* — g^+ = 0.23, p = 0.03, 95 % CI [0.03 to 0.44], k = 11, n = 503 — ☐ sign. ☒ n.s.<br>executive function — g^+ = 0.20, p = 0.08, 95 % CI [- 0.03 to 0.43], k = 11, n = 353 — ☒ sign. ☐ n.s.<br><br>☐ Brain / Neurochemicals / HRV<br>NR<br><br>☐ Physical Outcomes, Mobility, and Activities of Daily Living<br>NR<br><br>☐ Psychosocial Outcomes<br>NR
Liang et al. 2018 [9]	POPULATION:<br>☐ healthy older adults<br>☒ older adults with mNCD<br>☐ older adults with Dementia<br><br>INTERVENTION:<br>computerized cognitive training<br><br>COMPARISON:<br>mixed (cognitive interventions, control group alone or in any combination)	☒ Cognition<br>global cognition — SMD^+ = - 0.02, p = 0.588, 95 % CI [- 0.31 to 0.27], k = 4 — ☐ sign. ☒ n.s.<br><br>☐ Brain / Neurochemicals / HRV<br>NR<br><br>☐ Physical Outcomes, Mobility, and Activities of Daily Living<br>NR<br><br>☒ Psychosocial Outcomes<br>neuropsych. symptoms — SMD^+ = -0.61, p = NR, 95 % CI [-1.29 to 0.06], k = 1 — NR
Sala et al. 2018 [10]	POPULATION:<br>☒ healthy older adults<br>☐ older adults with mNCD<br>☐ older adults with Dementia<br><br>INTERVENTION:<br>video game playing<br><br>COMPARISON:<br>mixed (active or passive control conditions)	☒ Cognition<br>intelligence/reasoning** — g^- = -0.63, p = 0.004, 95 % CI [-1.06 to – 0.20], k = 15 — NR<br>memory — g^- = -0.01, p = 0.92, 95 % CI [-0.24 to 0.22], k = 22 — NR<br>visual attention — g^- = 0.00, p = 0.97, 95 % CI [-0.21 to 0.22], k = 26 — NR<br>spatial ability — g^- = 0.06, p = 0.65, 95 % CI [-0.20 to 0.33], k = 12 — NR<br>cognitive control — g^- = 0.07, p = 0.54, 95 % CI [-0.15 to 0.29, k = 17 — NR<br><br>☐ Brain / Neurochemicals / HRV<br>NR<br><br>☐ Physical Outcomes, Mobility, and Activities of Daily Living<br>NR<br><br>☐ Psychosocial Outcomes<br>NR

García-Casal et al. 2017 [11]	POPULATION:<br>☐ healthy older adults<br>☐ older adults with mNCD<br>☒ older adults with Dementia<br><br>INTERVENTION:<br>computer-based cognitive interventions (i.e. cognitive recreation, cognitive rehabilitation, cognitive stimulation or cognitive training)<br><br>COMPARISON:<br>mixed (active and passive control, control populations)	☒ Cognition<br><br>global cognition** — $SMD^+ = 0.69$, $p < 0.0001$, 95 % CI [0.37 to 1.02], k = 7, n = 81 — ☐ sign. ☒ n.s.<br><br>☐ Brain / Neurochemicals / HRV<br><br>NR<br><br>☐ Physical Outcomes, Mobility, and Activities of Daily Living<br><br>NR<br><br>☒ Psychosocial Outcomes<br><br>depression** — $SMD^+ = 0.47$, $p = 0.003$, 95 % CI [0.16 to 0.78], k = 6, n = 84 — ☐ sign. ☒ n.s.<br>anxiety* — $SMD^+ = 0.55$, $p = 0.03$, 95 % CI [0.07 to 1.04], k = 2, n = 34 — ☐ sign. ☒ n.s.<br>ADL — $SMD^+ = 0.26$, $p = 0.11$, 95 % CI [0.06 to 0.59] — NR
Hill et al. 2017 [12]	POPULATION:<br>☐ healthy older adults<br>☒ older adults with mNCD<br>☒ older adults with Dementia<br><br>INTERVENTION:<br>computerized cognitive training<br><br>COMPARISON:<br>(1) active (e.g., sham CCT, psychoeducation), or pencil-and-paper cognitive training)<br>(2) passive (no-contact, wait-list)	☒ Cognition<br><br>MNCD:<br>pooled estimate** (1) — $g^+ = 0.40$, $p < 0.001$, 95 % CI [0.17 to 0.63], k = 11 — ☐ sign. ☒ n.s.<br>global cognition** (1) — $g^+ = 0.41$, $p < 0.001$, 95 % CI [0.03 to 0.75], k = 8 — ☐ sign. ☒ n.s.<br>pooled estimate** (2) — $g^+ = 0.32$, $p < 0.001$, 95 % CI [0.09 to 0.55], k = 6 — ☐ sign. ☒ n.s.<br>global cognition** (2) — $g^+ = 0.37$, $p < 0.001$, 95 % CI [0.02 to 0.72], k = 4 — ☐ sign. ☒ n.s.<br>working memory** — $g^+ = 0.74$, $p < 0.001$, 95 % CI [0.32 to 1.15], k = 9 — ☒ sign. ☐ n.s.<br>nonverbal learning** — $g^+ = 0.50$, $p < 0.001$, 95 % CI [0.25 to 0.76], k = 8 — ☐ sign. ☒ n.s.<br>attention** — $g^+ = 0.44$, $p < 0.001$, 95 % CI [0.20 to 0.68], k = 6 — NR<br>verbal memory** — $g^+ = 0.42$, $p < 0.001$, 95 % CI [0.21 to 0.63], k = 12 — ☒ sign. ☐ n.s.<br>verbal learning** — $g^+ = 0.39$, $p < 0.002$, 95 % CI [0.14 to 0.63], k = 11 — ☒ sign. ☐ n.s.<br>language — $g^+ = 0.41$, $p > 0.05$, 95 % CI [-0.10 to 0.92], k = 6 — NR<br>nonverbal memory — $g^+ = 0.20$, $p > 0.05$, 95 % CI [-0.03 to 0.43], k = 7 — NR<br>executive function — $g^+ = 0.20$, $p > 0.05$, 95 % CI [-0.05 to 0.44], k = 13 — NR<br>visuospatial skills — $g^+ = 0.18$, $p > 0.05$, 95 % CI [-0.23 to 0.60], k = 5 — NR<br>processing speed — $g^+ = 0.09$, $p > 0.05$, 95 % CI [-0.17 to 0.35], k = 7 — NR<br>Dementia:<br>visuospatial skills* — $g^+ = 0.54$, $p = 0.02$, 95 % CI [0.07 to 1.01], k = 3 — NR<br>pooled estimate * — $g^+ = 0.26$, $p = 0.05$, 95 % CI [0.01 to 0.52], k = 11 — ☐ sign. ☒ n.s.<br>verbal learning — $g^+ = 0.42$, $p = 0.14$, 95 % CI [-0.14 to 0.97], k = 4 — NR<br>global cognition — $g^+ = 0.31$, $p = 0.15$, 95 % CI [-0.11 to 0.72], k = 7 — NR<br>non-verbal learning — $g^+ = 0.23$, $p = 0.50$, 95 % CI [-0.43 to 0.90], k = 2 — NR<br>working memory — $g^+ = 0.22$, $p = 0.45$, 95 % CI [-0.34 to 0.78], k = 4 — NR<br>verbal memory — $g^+ = 0.17$, $p = 0.42$, 95 % CI [-0.25 to 0.59], k = 9 — NR<br>processing speed — $g^+ = 0.11$, $p = 0.69$, 95 % CI [-0.43 to 0.66], k = 2 — NR<br>language — $g^+ = 0.08$, $p = 0.67$, 95 % CI [-0.29 to 0.44], k = 4 — NR<br>executive function — $g^+ = 0.02$, $p = 0.92$, 95 % CI [-0.46 to 0.51], k = 5 — NR<br>non-verbal memory — $g^+ = -0.06$, $p = 0.80$, 95 % CI [-0.06 to 0.38], k = 1 — N/A<br>attention — $g^+ = -0.19$, $p = 0.52$, 95 % CI [-0.77 to 0.39], k = 2 — NR<br><br>☐ Brain / Neurochemicals / HRV<br><br>NR<br><br>☐ Physical Outcomes, Mobility, and Activities of Daily Living<br><br>mNCD:<br>instrumental ADL — $g^+ = 0.21$, $p > 0.05$, 95 % CI [- 0.18 to 0.61], k = 6 — NR<br>Dementia:<br>ADL — $g^+ = 0.06$, $p = 0.84$, 95 % CI [- 0.51 to 0.64], k = 2 — NR<br>instrumental ADL — $g^+ = - 0.24$, $p = 0.07$, 95 % CI [- 0.50 to 0.02], k = 6 — NR<br><br>☒ Psychosocial Outcomes<br><br>mNCD:<br>psychosocial function* — $g^+ = 0.52$, $p = 0.045$, 95 % CI [0.01 to 1.03], k = 8 — ☒ sign. ☐ n.s.<br>Dementia:<br>psychosocial function — $g^+ = 0.60$, $p = 0.07$, 95 % CI [- 0.05 to 1.25], k = 6 — NR
Mewborn et al. 2017 [13]	POPULATION:<br>☒ healthy older adults<br>☒ older adults with mNCD<br>☐ older adults with Dementia<br><br>INTERVENTION:<br>single- and multi-domain cognitive training interventions<br><br>COMPARISON:<br>mixed (passive or active (e.g. education and support groups, completing questionnaires, and	☒ Cognition<br><br>pooled effect for HOA** — $g^+ = 0.314$, $p < 0.001$, 95 % CI [0.24 to 0.39], k = 134 — ☒ sign. ☐ n.s.<br>pooled effect for — $g^+ = 0.336$, $p < 0.001$, 95 % CI [0.21 to 0.47], k = 38 — ☒ sign. ☐ n.s.<br>mNCD** — $g^+ = 0.438$, $p < 0.001$, 95 % CI [0.36 to 0.52], k = 141 — ☒ sign. ☐ n.s.<br>near transfer (mixed)** — $g^+ = 0.145$, $p < 0.001$, 95 % CI [0.09 to 0.20], k = 138 — ☒ sign. ☐ n.s.<br>far transfer (mixed)**<br><br>☐ Brain / Neurochemicals / HRV<br><br>NR<br><br>☐ Physical Outcomes, Mobility, and Activities of Daily Living<br><br>NR

	cognitive stimulation (e.g., computer games, puzzles, reading)) without statistically difference between the pooled estimate between these two control groups)	☐ Psychosocial Outcomes<br>NR
Shermann et al. 2017 [14]	POPULATION:<br>☐ healthy older adults<br>☒ older adults with mNCD<br>☐ older adults with Dementia<br><br>INTERVENTION:<br>broad selection of cognitive training interventions (i.e. cognitive stimulation = 0/26 (0 %), restorative training = 8/26 (30.77%), compensatory training = 3/26 (11.54%), and multicomponent approaches = 15/26 (57.69%))<br><br>COMPARISON:<br>mixed (i.e. passive (received no training), waitlisted, or provided standard of care (57.69 %), or active (engaged in an active, non-trained program (42.31 %).	☒ Cognition<br>memory (verbal)** — g^+ = 0.758, p < 0.001, 95 % CI [0.382 to 1.133], k = 15 — ☒ sign. ☐ n.s.<br>memory (pooled)** — g^+ = 0.659, p = 0.000, 95 % CI [0.383 to 0.936], k = 20 — ☒ sign. ☐ n.s.<br>working memory** — g^+ = 0.614, p = 0.000, 95 % CI [0.285 to 0.943], k = 12 — ☒ sign. ☐ n.s.<br>executive function* — g^+ = 0.575, p = 0.019, 95 % CI [0.093 to 1.056], k = 13 — ☒ sign. ☐ n.s.<br>memory (non-verbal)** — g^+ = 0.570, p = 0.006, 95 % CI [0.160 to 0.980], k = 5 — ☐ sign. ☒ n.s.<br>language** — g^+ = 0.511, p < 0.001, 95 % CI [0.231 to 0.790], k = 7 — ☒ sign. ☐ n.s.<br>overall** — g^+ = 0.454, p = 0.003, 95 % CI [0.156 to 0.753], k = 26 — ☒ sign. ☐ n.s.<br>global cognition** — g^+ = 0.216, p = 0.003, 95 % CI [0.076 to 0.356], k = 16 — ☐ sign. ☒ n.s.<br>processing speed — g^- = - 0.434, p = 0.235, 95 % CI [-1.150 to 0.282], k = 6 — ☒ sign. ☐ n.s.<br><br>☐ Brain / Neurochemicals / HRV<br>NR<br><br>☐ Physical Outcomes, Mobility, and Activities of Daily Living<br>NR<br><br>☐ Psychosocial Outcomes<br>NR
Melby-Lervåg et al. 2016 [15]	POPULATION:<br>☒ healthy older adults<br>☐ older adults with mNCD<br>☐ older adults with Dementia<br><br>INTERVENTION:<br>computerized working memory training<br><br>COMPARISON:<br>(1) treated (non-working memory training intervention of a similar type and of equivalent intensity and duration)<br>(2) untreated (no intervention)	☒ Cognition<br>near-transfer ** (1) — g^+ = 0.76, p < 0.01, k = 4<br>working memory (1) — g^+ = 0.28, p > 0.05, k = 6<br>nonverbal abilities (1) — g^+ = -0.13, p > 0.05, k = 6<br>near-transfer* (2) — g^+ = 1.37, p < 0.05, k = 7<br>working memory** (2) — g^+ = 0.49, p < 0.01, k = 10<br>nonverbal abilities* (2) — g^+ = 0.22, p < 0.05, k = 10<br>visuo-spatial skills (2) — g^+ = 0.47, p > 0.01, k = 4<br><br>☐ Brain / Neurochemicals / HRV<br>NR<br><br>☐ Physical Outcomes, Mobility, and Activities of Daily Living<br>NR<br><br>☐ Psychosocial Outcomes<br>NR
Toril et al. 2014 [16]	POPULATION:<br>☒ healthy older adults<br>☐ older adults with mNCD<br>☐ older adults with Dementia<br><br>INTERVENTION:<br>video game training (i.e. commercial video games or combinations of classic cognitive tasks taken from commercial packages (e.g. Nintendo Brain Training, Brain Age, Big Brain Academy, etc.)<br><br>COMPARISON:<br>mixed (active control intervention and passive control (no intervention)) without significant difference in effect estimates between control conditions	☒ Cognition<br>reaction time** — d^+ = 0.63, p < 0.01, 95 % CI [0.42 to 0.84] — NR<br>memory* — d^+ = 0.39, p < 0.05, 95 % CI [0.01 to 0.64] — NR<br>global cognition* — d^+ = 0.38, p < 0.05, 95 % CI [0.13 to 0.64] — NR<br>pooled effect** — d^+ = 0.37, p < 0.01, 95 % CI [0.26 to 0.48] — ☒ sign. ☐ n.s.<br>attention** — d^+ = 0.37, p < 0.01, 95 % CI [0.17 to 0.57] — NR<br>executive function — d^+ = 0.16, p > 0.05, 95 % CI [-0.10 to 0.42] — NR<br><br>☐ Brain / Neurochemicals / HRV<br>NR<br><br>☐ Physical Outcomes, Mobility, and Activities of Daily Living<br>NR<br><br>☐ Psychosocial Outcomes<br>NR

Lampit et al. 2014 [17]	POPULATION:<br>☒ healthy older adults<br>☐ older adults with mNCD<br>☐ older adults with Dementia<br><br>INTERVENTION:<br>computerized cognitive training<br><br>COMPARISON:<br>mixed (active control intervention and passive control (no intervention)) without significant difference in effect estimates between control conditions	☒ Cognition<br><br>processing speed** $g^+ = 0.31$, $p = 0.002$, 95 % CI [0.11 to 0.50], $k = 33$ ☒ sign. ☐ n.s<br>nonverbal memory** $g^+ = 0.24$, $p = 0.002$, 95 % CI [0.09 to 0.38], $k = 13$ ☐ sign. ☒ n.s<br>pooled effect** $g^+ = 0.22$, $p < 0.001$, 95 % CI [0.15 to 0.29], $k = 52$ ☒ sign. ☐ n.s<br>working memory** $g^+ = 0.22$, $p < 0.001$, 95 % CI [0.09 to 0.35], $k = 28$ ☒ sign. ☐ n.s<br>verbal memory* $g^+ = 0.16$, $p = 0.02$, 95 % CI [0.03 to 0.29], $k = 3$ ☒ sign. ☐ n.s<br>visuospatial skills** $g^+ = 0.02$, $p = 0.01$, 95 % CI [0.15 to 0.29], $k = 8$ ☐ sign. ☒ n.s<br>executive function $g^+ = 0.09$, $p = 0.096$, 95 % CI [-0.02 to 0.19], $k = 29$ ☒ sign. ☐ n.s<br>attention $g^+ = 0.024$, $p = 0.06$, 95 % CI [-0.01 to 0.50], $k = 11$ ☒ sign. ☐ n.s<br><br>☐ Brain / Neurochemicals / HRV<br><br>NR<br><br>☐ Physical Outcomes, Mobility, and Activities of Daily Living<br><br>NR<br><br>☐ Psychosocial Outcomes<br><br>NR
Karbach et al. 2014 [18]	POPULATION:<br>☒ healthy older adults<br>☐ older adults with mNCD<br>☐ older adults with Dementia<br><br>INTERVENTION:<br>cognitive training interventions<br><br>COMPARISON:<br>mixed (passive or active without statistically difference between the pooled estimate between these two control groups)	☒ Cognition<br><br>target measure $_{(pooled)}$** $\Delta SMD_{exp}^+ = 0.91$, $p < 0.001$, 95 % CI [0.83 to 1.00], $k = 28$ ☒ sign. ☐ n.s.<br>near transfer $_{(pooled)}$** $\Delta SMD_{exp}^+ = 0.68$, $p < 0.001$, 95 % CI [0.56 to 0.79], $k = 9$ ☒ sign. ☐ n.s.<br>far transfer $_{(pooled)}$** $\Delta SMD_{exp}^+ = 0.37$, $p < 0.001$, 95 % CI [0.24 to 0.49], $k = 4$ ☐ sign. ☒ n.s.<br><br>☐ Brain / Neurochemicals / HRV<br><br>NR<br><br>☐ Physical Outcomes, Mobility, and Activities of Daily Living<br><br>NR<br><br>☐ Psychosocial Outcomes<br><br>NR

Table S2: Summary of meta-analytic results for physical training interventions
color-coding: black = significant effect, grey = no significant effect
* = significant at p ≤ 0.05, ** = significant at p ≤ 0.01

Abbreviations: ADAS-cog = Alzheimer Disease Assessment Scale-Cognitive Subscale, ADL = Activities of daily living, BDNF = brain derived neurotrophic factor, CI = confidence interval, DSF = digit span forward, DSB = digit span backward, DSF-B = Δ digit span forward – digit span backward, g = Hedge's g, GRADE = Grading of Recommendations Assessment, Development and Evaluation, HF-HRV = high frequency heart rate variability, HOA = healthy older adults; mNCD = mild neurocognitive disorder, MD = mean difference (absolut), MMSE = Mini-Mental State Examination, MoCA = Montreal Cognitive Assessment, k = number of studies; n = number of participants; NR = not reported, RAVL = Rey Auditory Verbal Learning Task, RMSSD = root mean square of successive RR interval differences, ST-Ptot = short-term total frequency power, ST-SDNN = short-term Standard Deviation of RR-Intervals SMD = standardized mean difference, ST-A = Stroop test A, ST-B = Stroop test B, TMT-A = Trail Making Test Part 1, TMT-B = Trail Making Test Part B, 24 h-SDNN = mean Standard Deviation of RR-Intervals over a time period of 24 h, 24 h-Ptot = mean total frequency power over a time period of 24 h

Reference	Study Characteristics	Outcomes			
		Type	Statistics	Heterogeneity	GRADE
Biazus-Sehn et al. 2020 [19]	POPULATION:<br>☐ healthy older adults<br>☒ older adults with mNCD<br>☐ older adults with Dementia<br><br>INTERVENTION:<br>☒ aerobic training<br>☒ resistance training<br>☐ balance training<br>☒ multicomponent physical training<br>☐ mixed physical training<br><br>COMPARISON:<br>mixed (i.e. no treatment; balance, tone or stretching programs; or social and/or mental activities)	☒ Cognition			
		pooled estimates			
		global cognition**	SMD^+ = 0.35, p < 0.001, 95 % CI [0.17 to 0.43], k = 18, n = 1,472	☐ sign. ☒ n.s.	
		executive function*	SMD^+ = 0.21, p = 0.026, 95 % CI [0.03 to 0.40], k = 19, n = 1,072	☐ sign. ☒ n.s.	
		delayed recall*	SMD^+ = 0.18, p = 0.047, 95 % CI [0.00 to 0.36], k = 17, n = 1,569	☐ sign. ☒ n.s.	
		verbal fluency	SMD^+ = 0.27, p = 0.069, 95 % CI [-0.02 to 0.56], k = 11, n = 1,115	NR	
		attention	SMD^+ = 0.17, p = 0.073, 95 % CI [-0.02 to 0.36], k = 4, n = 467	NR	
		immediate recall	SMD^+ = 0.09, p = 0.31, 95 % CI [-0.09 to 0.26], k = 9, n = 679	NR	
		cognitive speed	SMD^+ = 0.02, p = 0.79, 95 % CI [-0.15 to 0.20], k = 11, n = 642	NR	
		working memory	SMD^+ = -0.01, p = 0.939, 95 % CI [-0.03 to -0.30], k = 15, n = 849	NR	
		aerobic training only			
		global cognition	SMD^+ = 0.19, p = 0.364, 95 % CI [-0.22 to 0.59]	NR	
		executive function	SMD^+ = 0.13, p = 0.407, 95 % CI [-0.18 to 0.44]	NR	
		delayed recall	SMD^+ = 0.07, p = 0.596, 95 % CI [-0.19 to 0.34]	NR	
		resistance training only			
		global cognition	SMD^+ = 0.19, p = 0.488, 95 % CI [-0.35 to 0.74]	NR	
		executive function	SMD^+ = 0.14, p = 0.499, 95 % CI [-0.26 to 0.53]	NR	
		delayed recall	SMD^+ = -0.04, p = 0.856, 95 % CI [-0.44 to 0.37]	NR	
		multicomp. training only			
		global cognition	SMD^+ = 0.38, p = 0.066, 95 % CI [-0.03 to 0.79]	NR	
		executive function	SMD^+ = 0.04, p = 0.928, 95 % CI [-0.81 to 0.90]	NR	
		delayed recall	SMD^+ = 0.01, p = 0.973, 95 % CI [-0.38 to 0.39]	NR	
		☐ Brain / Neurochemicals / HRV			
		NR			
		☐ Physical Outcomes, Mobility, and Activities of Daily Living			
		NR			
		☐ Psychosocial Outcomes			
		NR			
Chen et al. 2020 [20]	POPULATION:<br>☒ healthy older adults<br>☒ older adults with mNCD<br>☐ older adults with Dementia<br><br>INTERVENTION:<br>☐ aerobic training<br>☐ resistance training<br>☐ balance training<br>☐ multicomponent physical training<br>☒ mixed physical training<br><br>COMPARISON:<br>mixed (no contact, no treatment, waiting list, sham exercise, and alternative active treatments for the comparison condition	☒ Cognition			
		pooled older adults:			
		shifting*	g^+ = 0.27, p < 0.05, 95 % CI [0.19 to 0.36], k = 34	NR	
		overall effect (resistance)*	g^+ = 0.22, p < 0.05, 95 % CI [0.10 to 0.33], k = 20	NR	
		overall (executive funct.)*	g^+ = 0.21, p < 0.05, 95 % CI [0.17 to 0.26], k = 107	☒ sign. ☐ n.s.	
		working memory*	g^+ = 0.19, p < 0.05, 95 % CI [0.12 to 0.26], k = 42	NR	
		inhibition*	g^+ = 0.14, p < 0.05, 95 % CI [0.04 to 0.24], k = 27	NR	
		overall effect (aerobic)*	g^+ = 0.14, p < 0.05, 95 % CI [0.06 to 0.33], k = 45	NR	
		overall effect (combined)*	g^+ = 0.10, p < 0.05, 95 % CI [0.00 to 0.19], k = 18	NR	
		HOA:			
		pooled effect (HOA)*	g^+ = 0.26, p < 0.05, 95 % CI [0.20 to 0.32], k = 85	NR	
		mNCD:			
		pooled effect (mNCD)*	g^+ = 0.08, p < 0.05, 95 % CI [0.00 to 0.17], k = 22	NR	
		☐ Brain / Neurochemicals / HRV			
		☐ Physical Outcomes, Mobility, and Activities of Daily Living			

		☐ Psychosocial Outcomes
Zhou et al. 2020 [21]	POPULATION:<br>☐ healthy older adults<br>☒ older adults with mNCD<br>☐ older adults with Dementia<br><br>INTERVENTION:<br>☐ aerobic training<br>☐ resistance training<br>☐ balance training<br>☐ multicomponent physical training<br>☒ mixed physical training<br><br>COMPARISON:<br>mixed (i.e. sham (such as stretching activities), placebo or no treatment or health education)	☒ Cognition<br>executive function** (DSF, DSB, DSF-B ,ST-A, ST-B) SMD$^+$ = 0.66, p = 0.008, 95 % CI [0.17 to 1.15], k = 5, n = 243 ☒ sign. ☐ n.s.<br>verbal fluency** SMD$^+$ = 0.55, p = 0.001, 95 % CI [0.22 to 0.89], k = 2, n = 72 ☐ sign. ☒ n.s.<br>gen. cognition** (ADAS-cog) SMD$^-$ = - 0.45, p = 0.02, 95 % CI [- 0.82 to - 0.08], k = 2, n = 171 ☐ sign. ☒ n.s.<br>visuo-spatial skills* SMD$^+$ = 0.38, p = 0.03, 95 % CI [0.03 to 0.72], k = 2, n = 132 ☐ sign. ☒ n.s.<br>memory** SMD$^+$ = 0.37, p = 0.001, 95 % CI [0.15 to 0.60], k = 4, n = 295 ☐ sign. ☒ n.s.<br>gen. cog. **(MMSE, MoCA) SMD$^+$ = 0.32, p = 0.005, 95 % CI [0.10 to 0.54], k = 5, n = 355 ☐ sign. ☒ n.s.<br>executive funct. (TMT B-A) SMD$^-$ = 0.25, p = 0.45, 95 % CI [- 0.88 to 0.39], k = 3, n = 203 ☒ sign. ☐ n.s.<br>☐ Brain / Neurochemicals / HRV<br>NR<br>☐ Physical Outcomes, Mobility, and Activities of Daily Living<br>NR<br>☐ Psychosocial Outcomes<br>NR
Law et al. 2020 [22]	POPULATION:<br>☐ healthy older adults<br>☒ older adults with mNCD<br>☐ older adults with Dementia<br><br>INTERVENTION:<br>☒ aerobic training<br>☐ resistance training<br>☐ balance training<br>☐ multicomponent physical training<br>☒ mixed physical training<br><br>COMPARISON:<br>mixed (i.e. no intervention or training plus other intervention vs. other intervention only)	☒ Cognition<br>pooled estimates:<br>global cognition** SMD$^+$ = 0.42, p < 0.001, 95 % CI [0.25 to 0.59], k = 26, n = 2,079 ☒ sign. ☐ n.s. low<br>working memory* SMD$^+$ = 0.26, p = 0.02, 95 % CI [0.03 to 0.49], k = 8, n = 646 ☒ sign. ☐ n.s. low<br>recognition SMD$^+$ = 0.31, p = 0.28, 95 % CI [-0.25 to 0.88], k = 6, n = 291 ☐ sign. ☒ n.s. low<br>delayed memory SMD$^+$ = 0.15, p = 0.12, 95 % CI [-0.04 to 0.33], k = 11, n = 1,294 ☒ sign. ☐ n.s. low<br>language SMD$^+$ = 0.15, p = 0.11, 95 % CI [-0.03 to 0.34], k = 15, n = 1,593 ☒ sign. ☐ n.s. very low<br>attention SMD$^+$ = 0.04, p = 0.52, 95 % CI [-0.07 to 0.15], k = 15, n = 1,294 ☐ sign. ☒ n.s. moderate<br>cogn. flexibility SMD$^+$ = 0.03, p = 0.74, 95 % CI [-0.14 to 0.20], k = 8, n = 544 ☐ sign. ☒ n.s. moderate<br>aerobic training only:<br>global cognition** SMD$^+$ = 0.45, 95 % CI [0.15 to 0.76], k = 11, n = 704 ☐ sign. ☒ n.s. moderate<br>attention SMD$^+$ = 0.12, 95 % CI [-0.06 to 0.30], k = 6, n = 481 ☐ sign. ☒ n.s.<br>cogn. flexibility SMD$^+$ = 0.01, 95 % CI [-0.16 to 0.19], k = 6, n = 522 ☐ sign. ☒ n.s.<br>multicomp. training only:<br>global cognition** SMD$^+$ = 0.43, 95 % CI [0.13 to 0.73], k = 9, n = 831 ☒ sign. ☐ n.s.<br>☐ Brain / Neurochemicals / HRV<br>NR<br>☐ Physical Outcomes, Mobility, and Activities of Daily Living<br>NR<br>☒ Psychosocial Outcomes<br>behavioral problems** SMD$^+$ = 0.35, p = 0.01, 95 % CI [0.07 to 0.64], k = 9, n = 1,172 ☐ sign. ☒ n.s. moderate
Jia et al. 2019 [23]	POPULATION:<br>☐ healthy older adults<br>☒ older adults with mNCD<br>☐ older adults with Dementia<br><br>INTERVENTION:<br>☒ aerobic training<br>☐ resistance training<br>☐ balance training<br>☐ multicomponent physical training<br>☐ mixed physical training<br><br>COMPARISON:<br>non-diet, non-training control group	☒ Cognition<br>global cognition (MMSE)** SMD$^+$ = 1.12, p < 0.001, 95 % CI [0.66 to 1.59], k = 13, n = 673 ☒ sign. ☐ n.s.<br>☐ Brain / Neurochemicals / HRV<br>NR<br>☐ Physical Outcomes, Mobility, and Activities of Daily Living<br>NR<br>☐ Psychosocial Outcomes<br>NR

Marinus et al. 2019 [24]	POPULATION:<br>☒ healthy older adults<br>☐ older adults with mNCD<br>☐ older adults with Dementia<br><br>INTERVENTION:<br>☒ aerobic training<br>☒ resistance training<br>☐ balance training<br>☒ multicomponent physical training<br>☐ mixed physical training<br><br>COMPARISON:<br>mixed (i.e. comparison to another training intervention, control intervention (no training) or pre-intervention values))	☐ Cognition<br>NR<br>☒ Brain / Neurochemicals / HRV<br>$[BDNF]_{blood\ (pooled)}$** $g^+ = 0.53$, $p < 0.001$, 95 % CI [0.31 to 0.75], k = 11, n = 464 ☒ sign. ☐ n.s.<br>$\Delta[BDNF]_{blood\ (multicomp.)}$** $g^- = -0.43$, $p < 0.001$, 95 % CI [-0.71 to -0.15], k = 4, n = 103 ☐ sign. ☒ n.s.<br>$\Delta[BDNF]_{blood\ (resistance)}$** $g^- = -0.22$, $p = 0.003$, 95 % CI [-0.37 to -0.07], k = 10, n = 361 ☒ sign. ☐ n.s.<br>$\Delta[BDNF]_{blood\ (pooled)}$** $g^- = -0.20$, $p < 0.001$, 95 % CI [-0.30 to -0.09], k = 20, n = 730 ☒ sign. ☐ n.s.<br>$\Delta[BDNF]_{blood\ (aerobic)}$ $g^- = -0.07$, $p = 0.41$, 95 % CI [-0.24 to 0.10], k = 5, n = 266 ☒ sign. ☐ n.s.<br>☐ Physical Outcomes, Mobility, and Activities of Daily Living<br>NR<br>☐ Psychosocial Outcomes<br>NR
Raffin et al. 2019 [25]	POPULATION:<br>☒ healthy older adults<br>☐ older adults with mNCD<br>☐ older adults with Dementia<br><br>INTERVENTION:<br>☒ aerobic training<br>☐ resistance training<br>☐ balance training<br>☐ multicomponent physical training<br>☐ mixed physical training<br><br>COMPARISON:<br>NR	☐ Cognition<br>NR<br>☒ Brain / Neurochemicals / HRV / HRV<br>overall study analysis:<br>24 h-SDNN** $g^+ = 0.442$, $p = 0.004$, 95 % CI [0.144 to 0.740], k = 3, n = 52 ☐ sign. ☒ n.s.<br>ST-SDNN** $g^+ = 0.366$, $p < 0.001$, 95 % CI [0.366 to 0.185], k = 5, n = 66 ☐ sign. ☒ n.s.<br>24 h-P_{tot} $g^+ = 1.334$, $p = 0.060$, 95 % CI [- 0.057 to 2.724], k = 2, n = 25 ☒ sign. ☐ n.s.<br>HF-HRV $g^+ = 0.089$, $p = 0.260$, 95 % CI [-0.066 to 0.243], k = 12, n = 200 ☐ sign. ☒ n.s.<br>RMSSD $g^+ = 0.078$, $p = 0.581$, 95 % CI [-0.201 to 0.358], k = 6, n = 114 ☐ sign. ☒ n.s.<br>ST-P_{tot} $g^+ = -0.055$, $p = 0.774$, 95 % CI [-0.434 to 0.323], k = 4, n = 73 ☐ sign. ☒ n.s.<br>controlled study analysis:<br>24 h-SDNN** $g^+ = 0.721$, $p = 0.008$, 95 % CI [0.184 to 1.257], k = 3, n = 106 ☐ sign. ☒ n.s.<br>24 h-P_{tot}** $g^+ = 0.731$, $p = 0.008$, 95 % CI [0.195 to 1.267], k = 2, n = 55 ☐ sign. ☒ n.s.<br>ST-SDNN $g^+ = 0.605$, $p = 0.075$, 95 % CI [-0.062 to 1.271], k = 2, n = 63 ☐ sign. ☒ n.s.<br>HF-HRV $g^+ = 0.236$, $p = 0.086$, 95 % CI [-0.033 to 0.506], k = 6, n = 205 ☐ sign. ☒ n.s.<br>RMSSD $g^+ = 0.299$, $p = 0.105$, 95 % CI [-0.062 to 0.659], k = 4, n = 116 ☐ sign. ☒ n.s.<br>☐ Physical Outcomes, Mobility, and Activities of Daily Living<br>NR<br>☐ Psychosocial Outcomes<br>NR
Sanders et al. 2019 [26]	POPULATION:<br>☒ healthy older adults<br>☒ older adults with mNCD<br>☐ older adults with Dementia<br><br>INTERVENTION:<br>☒ aerobic training<br>☒ resistance training<br>☐ balance training<br>☒ multicomponent physical training<br>☒ mixed physical training<br><br>COMPARISON:<br>contrasting activities (i.e. including non-physical activities or stretching and toning)	☒ Cognition<br>HOA:<br>execut. funct.$_{(pooled)}$** $g^+ = 0.34$, $p < 0.01$, 95 % CI [0.20 to 0.47], k = 28, n = 1,430 ☐ sign. ☒ n.s.<br>memory$_{(pooled)}$** $g^+ = 0.31$, $p < 0.01$, 95 % CI [0.10 to 0.53], k = 11, n = 589 ☐ sign. ☒ n.s.<br>overall estimate$_{(pooled)}$** $g^+ = 0.26$, $p < 0.01$, 95 % CI [0.15 to 0.37], k = 23, n = 1,225 NR<br>overall est.$_{(resistance)}$** $g^+ = 0.27$, $p < 0.01$, 95 % CI [0.09 to 0.46] NR<br>overall est.$_{(aerobic)}$** $g^+ = 0.22$, $p < 0.01$, 95 % CI [0.03 to 0.41] NR<br>overall est.$_{(mult.comp)}$ $g^+ = 0.30$, $p > 0.05$, 95 % CI [-0.01 to 0.60] NR<br>global cognition$_{(pooled)}$ $g^+ = 0.10$, $p > 0.05$, 95 % CI [-0.04 to 0.24], k = 5, n = 314 ☐ sign. ☒ n.s.<br>mNCD:<br>global cognition$_{(pooled)}$** $g^+ = 0.47$, $p < 0.01$, 95 % CI [0.19 to 0.74], k = 11, n = 699 ☐ sign. ☒ n.s.<br>overall est.$_{(multicomp)}$** $g^+ = 0.36$, $p < 0.01$, 95 % CI [0.04 to 0.68] NR<br>execut. function$_{(pooled)}$* $g^+ = 0.24$, $p < 0.05$, 95 % CI [0.02 to 0.47], k = 6, n = 358 ☐ sign. ☒ n.s.<br>overall est.$_{(pooled)}$* $g^+ = 0.22$, $p < 0.05$, 95 % CI [0.05 to 0.39], k = 13, n =782 NR<br>overall est.$_{(aerobic)}$ $g^+ = 0.22$, $p > 0.05$, 95 % CI [-0.20 to 0.64] NR<br>overall est.$_{(aerobic)}$ $g^+ = 0.22$, $p > 0.05$, 95 % CI [-0.20 to 0.64] NR<br>memory$_{(pooled)}$ $g^+ = 0.11$, $p > 0.05$, 95 % CI [-0.07 to 0.28], k = 6, n = 311 ☐ sign. ☒ n.s.<br>☐ Brain / Neurochemicals / HRV<br>NR<br>☐ Physical Outcomes, Mobility, and Activities of Daily Living<br>NR<br>☐ Psychosocial Outcomes<br>NR

Wang et al. 2019 [27]	POPULATION:<br>☐ healthy older adults<br>☒ older adults with mNCD<br>☐ older adults with Dementia<br><br>INTERVENTION:<br>☒ aerobic training<br>☒ resistance training<br>☐ balance training<br>☐ multicomponent physical training<br>☐ mixed physical training<br><br>COMPARISON:<br>mixed (i.e. stretching, education, usual lifestyle, social activities)	☒ Cognition<br><br>global cogn. $_{(aerobic)}$** SMD^+ = 0.80, p < 0.001, 95 % CI [0.55 to 1.05], k = 5, n = 308 ☐ sign. ☒ n.s.<br>global cogn. $_{(resistance)}$** SMD^+ = 0.80, p = 0.002, 95 % CI [0.29 to 1.32], k = 4, n = 146 ☐ sign. ☒ n.s.<br><br>☐ Brain / Neurochemicals / HRV<br><br>NR<br><br>☐ Physical Outcomes, Mobility, and Activities of Daily Living<br><br>NR<br><br>☐ Psychosocial Outcomes<br><br>NR
Vaportzis et al. 2019 [7]	POPULATION:<br>☒ healthy older adults<br>☐ older adults with mNCD<br>☐ older adults with Dementia<br><br>INTERVENTION:<br>☐ aerobic training<br>☐ resistance training<br>☐ balance training<br>☐ multicomponent physical training<br>☒ mixed physical training<br><br>COMPARISON:<br>(1) active control intervention (e.g. stretching or balance classes)<br>(2) wait-list control<br>(3) passive control (no intervention)	☒ Cognition<br><br>digit span forward(1) g^+ = 2.48, p = 0.33, 95 % CI [- 2.50 to 7.46], k = 2, n = 109 ☒ sign. ☐ n.s.<br>stroop colour(1) g^+ = 0.23, p = 0.10, 95 % CI [- 0.04 to 0.51], k = 2, n = 245 ☐ sign. ☒ n.s.<br>TMT A/B(1) g^+ = 0.19, p = 0.32, 95 % CI [- 0.18 to 0.56], k = 2, n = 245 ☐ sign. ☒ n.s.<br>stroop interference(1) g^+ = 0.04, p = 0.77, 95 % CI [- 0.24 to 0.32], k = 3, n = 195 ☐ sign. ☒ n.s.<br>digit span backward(1) g^+ = 0.04, p = 0.80, 95 % CI [- 0.25 to 0.32], k = 3, n = 219 ☐ sign. ☒ n.s.<br>RAVL-delayed recall(2) g^+ = 0.24, p = 0.80, 95 % CI [- 0.08 to 0.55], k = 2, n = 159 ☐ sign. ☒ n.s.<br>RAVL-immediate recall(2) g^+ = 0.17, p = 0.28, 95 % CI [- 0.14 to 0.48], k = 2, n = 159 ☐ sign. ☒ n.s.<br>digit span backwards(2) g^+ = 0.05, p = 0.75, 95 % CI [- 0.26 to 0.36], k = 2, n = 159 ☐ sign. ☒ n.s.<br>TMT A(2) g^+ = 0.04, p = 0.62, 95 % CI [- 0.12 to 0.21], k = 3, n = 559 ☐ sign. ☒ n.s.<br>verbal fluency(2) g^+ = 0.02, p = 0.93, 95 % CI [- 0.32 to 0.35], k = 2, n = 135 ☐ sign. ☒ n.s.<br>TMT B(2) g^+ = 0.01, p = 0.94, 95 % CI [- 0.33 to 0.35], k = 2, n = 135 ☐ sign. ☒ n.s.<br>letter-number seq.(2) g^+ = -0.03, p = 0.85, 95 % CI [- 0.33 to 0.27], k = 2, n = 183 ☐ sign. ☒ n.s.<br>choice reaction time(2) g^+ = -0.08, p = 0.62, 95 % CI [- 0.38 to 0.23], k = 2, n = 183 ☐ sign. ☒ n.s.<br>verbal fluency*(3) g^+ = 0.31, p = 0.04, 95 % CI [0.02 to 0.61], k = 2, n = 221 ☐ sign. ☒ n.s.<br>TMT A*(3) g^+ = 0.43, p = 0.05, 95 % CI [- 0.00 to 0.86], k = 3, n = 331 ☒ sign. ☐ n.s.<br>digit symbol substit.*(3) g^+ = 0.30, p = 0.05, 95 % CI [- 0.00 to 0.59], k = 2, n = 221 ☐ sign. ☒ n.s.<br>delayed logical memory(3) g^+ = 0.19, p = 0.41, 95 % CI [- 0.26 to 0.64], k = 2, n = 226 ☐ sign. ☒ n.s.<br>logical memory(3) g^+ = 0.17, p = 0.45, 95 % CI [- 0.27 to 0.60], k = 2, n = 226 ☐ sign. ☒ n.s.<br>digit span forward(3) g^+ = 0.12, p = 0.26, 95 % CI [- 0.09 to 0.33], k = 3, n = 407 ☐ sign. ☒ n.s.<br><br>☐ Brain / Neurochemicals / HRV<br><br>NR<br><br>☐ Physical Outcomes, Mobility, and Activities of Daily Living<br><br>NR<br><br>☐ Psychosocial Outcomes<br><br>NR
Firth et al. 2018 [28]	POPULATION:<br>☒ healthy older adults<br>☐ older adults with mNCD<br>☐ older adults with Dementia<br><br>INTERVENTION:<br>☒ aerobic training<br>☐ resistance training<br>☐ balance training<br>☐ multicomponent physical training<br>☐ mixed physical training<br><br>COMPARISON:<br>mixed (i.e. inactive control, time-and-attention matched comparator of minimal activity (e.g. stretching, balance training), of a similar duration)	☐ Cognition<br><br>NR<br><br>☒ Brain / Neurochemicals / HRV<br><br>hippocampal volume $_{left}$** g^+ = 0.355, p = 0.001, 95 % CI [0.14 to 0.57], k = 5, n = 332 ☐ sign. ☒ n.s.<br>hippocampal volume $_{right}$* g^+ = 0.237, p = 0.032, 95 % CI [0.02 to 0.45], k = 5, n = 332 ☐ sign. ☒ n.s.<br>hippocampal volume $_{total}$ g^+ = 0.146, p = 0.07, 95 % CI [-0.011 to 0.303], k = 6, n = 390 ☐ sign. ☒ n.s.<br><br>☐ Physical Outcomes, Mobility, and Activities of Daily Living<br><br>NR<br><br>☐ Psychosocial Outcomes<br><br>NR

Lam et al.<br>2018 [29]	POPULATION:<br>☐ healthy older adults<br>☒ older adults with mNCD<br>☐ older adults with Dementia<br><br>INTERVENTION:<br>☒ aerobic training<br>☒ resistance training<br>☒ balance training<br>☒ multicomponent physical training<br>☒ mixed physical training<br><br>COMPARISON:<br>mixed (i.e. training versus no intervention/placebo or training plus other intervention versus other intervention only)	☐ Cognition<br>NR<br>☐ Brain / Neurochemicals / HRV<br>NR<br>☒ Physical Outcomes, Mobility, and Activities of Daily Living<br>endurance (6-min walk test)* MD^+ = 50, $p < 0.05$, 95 % CI [18 to 81], k = 7, n = 402 ☒ sign. ☐ n.s. moderate<br>ADL (Barthel Index)* MD^+ = 10, $p < 0.05$, 95 % CI [3 to 16], k = 4, n = 237 ☒ sign. ☐ n.s. low<br>step length [cm]* MD^+ = 5, $p < 0.05$, 95 % CI [2 to 8], k = 5, n = 296 ☐ sign. ☒ n.s. high<br>balance (funct. reach test)* MD^+ = 3.9, $p < 0.05$, 95 % CI [0.3 to 7.0], k = 6, n = 242 ☐ sign. ☒ n.s. high<br>balance (Berg-balance-scale)* MD^+ = 3.6, $p < 0.05$, 95 % CI [0.3 to 7.0], k = 6, n = 722 ☒ sign. ☐ n.s high<br>strength (30-second sit-to-stand)* MD^+ = 2.1, $p < 0.05$, 95 % CI [0.3 to 3.9], k = 4, n = 278 ☒ sign. ☐ n.s. moderate<br>walking speed [m/s]* MD^+ = 0.13, $p < 0.05$, 95 % CI [0.03 to 0.24], k = 7, n = 568 ☒ sign. ☐ n.s. moderate<br>mobility (Timed Up and Go)* MD^- = -1, $p < 0.05$, 95 % CI [-2 to 0], k = 11, n = 606 ☐ sign. ☒ n.s. moderate<br>fall rate OR = 0.98, $p > 0.05$, 95 % CI [0.49 to 1.95], k = 3, n = 191 ☐ sign. ☒ n.s. low<br>☐ Psychosocial Outcomes<br>NR
Liang et al.<br>2018 [9]	POPULATION:<br>☐ healthy older adults<br>☒ older adults with mNCD<br>☐ older adults with Dementia<br><br>INTERVENTION:<br>☐ aerobic training<br>☐ resistance training<br>☐ balance training<br>☐ multicomponent physical training<br>☒ mixed physical training<br><br>COMPARISON:<br>mixed (i.e. cognitive interventions, control group alone or in any combination)	☒ Cognition<br>global cognition (MMSE) SMD^+ = 0.35, $p > 0.05$, 95 % CI [-0.12 to 0.82], k = 4 ☒ sign. ☐ n.s.<br>☐ Brain / Neurochemicals / HRV<br>NR<br>☐ Physical Outcomes, Mobility, and Activities of Daily Living<br>NR<br>☐ Psychosocial Outcomes<br>NR
Northey et al.<br>2018 [30]	POPULATION:<br>☒ healthy older adults<br>☒ older adults with mNCD<br>☐ older adults with Dementia<br><br>INTERVENTION:<br>☒ aerobic training<br>☒ resistance training<br>☐ balance training<br>☒ multicomponent physical training<br>☐ mixed physical training<br><br>COMPARISON:<br>mixed (i.e. (1) sham training or alternative active treatment, (2) attention control, (3) no contact)	☒ Cognition<br>overall (pooled)(1) SMD^+ = 0.13, $p > 0.05$, 95 % CI [-0.06 to 0.32], n = 120 NR<br>overall (pooled)(2)** SMD^+ = 0.48, $p < 0.01$, 95 % CI [0.14 to 0.82], n = 17 NR<br>overall (pooled)(3)** SMD^+ = 0.34, $p < 0.01$, 95 % CI [0.17 to 0.51], n = 189 NR<br>memory (pooled)** SMD^+ = 0.36, $p < 0.01$, 95 % CI [0.22 to 0.50], n = 81 NR<br>executive funct. (pooled)** SMD^+ = 0.34, $p < 0.01$, 95 % CI [0.20 to 0.47], n = 94 NR<br>overall (mult.comp.)** SMD^+ = 0.33, $p < 0.01$, 95 % CI [0.14 to 0.53], n = 80 NR moderate<br>overall (pooled)** SMD^+ = 0.29, $p < 0.01$, 95 % CI [0.17 to 0.41], k = 36, n = 333 ☒ sign. ☐ n.s.<br>overall (resistance)** SMD^+ = 0.29, $p < 0.01$, 95 % CI [0.13 to 0.44], n = 80 NR<br>work. memory (pooled)** SMD^+ = 0.29, $p < 0.01$, 95 % CI [0.12 to 0.45], n = 81 NR<br>attention (pooled)** SMD^+ = 0.27, $p < 0.01$, 95 % CI [0.14 to 0.41], n = 87 NR<br>overall (aerobic)** SMD^+ = 0.24, $p < 0.01$, 95 % CI [0.10 to 0.37], n = 153 NR<br>global cognition (pooled) SMD^+ = 0.16, $p > 0.05$, 95 % CI [- 0.14 to 0.47], n = 6 NR<br>HOA<br>overall (pooled)** SMD^+ = 0.36, $p < 0.01$, 95 % CI [0.04 to 0.68], n = 41 NR<br>mNCD<br>overall (pooled)** SMD^+ = 0.28, $p < 0.01$, 95 % CI [0.11 to 0.44], n = 197 NR<br>☐ Brain / Neurochemicals / HRV<br>NR<br>☐ Physical Outcomes, Mobility, and Activities of Daily Living<br>NR<br>☐ Psychosocial Outcomes<br>NR

Panza et al. 2018 [31]	POPULATION:<br>☐ healthy older adults<br>☒ older adults with mNCD<br>☒ older adults with Dementia<br><br>INTERVENTION:<br>☒ aerobic training<br>☒ resistance training<br>☐ balance training<br>☒ multicomponent physical training<br>☐ mixed physical training<br><br>COMPARISON:<br>nondiet, non-training control group	☒ Cognition<br><br>pooled estimates<br>global cogn. (pooled)* $d^+ = 0.47$, $p < 0.05$, 95 % CI [0.26 to 0.68], k = 23, n = 1,150<br>global cogn. (aerobic)* $d^+ = 0.65$, $p < 0.05$, 95 % CI [0.35 to 0.95], k = 15<br>global cogn. (combined) $d^+ = 0.19$, $p > 0.05$, 95 % CI [-0.06 to 0.43], k = 8<br>mNCD<br>global cogn. (pooled)* $d^+ = 0.27$, $p < 0.05$, 95 % CI [0.05 to 0.50], k = 12<br>ADAS-cog (pooled)* $d^+ = 0.65$, $p < 0.05$, 95 % CI [0.31 to 0.99], k = 11<br><br>☐ Brain / Neurochemicals / HRV<br><br>NR<br><br>☐ Physical Outcomes, Mobility, and Activities of Daily Living<br><br>NR<br><br>☐ Psychosocial Outcomes<br><br>NR
de Souto et al. 2018 [32]	POPULATION:<br>☒ healthy older adults<br>☒ older adults with mNCD<br>☐ older adults with Dementia<br><br>INTERVENTION:<br>☐ aerobic training<br>☐ resistance training<br>☐ balance training<br>☐ multicomponent physical training<br>☒ mixed physical training<br><br>COMPARISON:<br>mixed (i.e. usual care or an active-control group, including control sham training groups, such as stretching and light-intensity training)	☒ Cognition<br><br>risk of mNCD onset $RR^- = 1.12$, $p = 0.49$, 95 % CI [0.81 to 1.55], k = 1, n = 686 N/A<br>risk for cogn. decline $RR^- = 0.90$, $p = 0.79$, 95 % CI [0.42 to 1.95], k = 2, n = 124 ☐ sign. ☒ n.s.<br>pooled estimate $RR^- = 0.74$, $p = 0.27$, 95 % CI [0.43 to 1.26], k = 2, n = 1,073 ☐ sign. ☒ n.s.<br>risk of dementia onset $RR^- = 0.56$, $p = 0.20$, 95 % CI [0.23 to 1.36], k = 3, n = 949 ☐ sign. ☒ n.s.<br><br>☐ Brain / Neurochemicals / HRV<br><br>NR<br><br>☐ Physical Outcomes, Mobility, and Activities of Daily Living<br><br>NR<br><br>☐ Psychosocial Outcomes<br><br>NR
Sherrington et al. 2017 [33]	POPULATION:<br>☒ healthy older adults<br>☒ older adults with mNCD<br>☐ older adults with Dementia<br><br>INTERVENTION:<br>☐ aerobic training<br>☐ resistance training<br>☐ balance training<br>☐ multicomponent physical training<br>☒ mixed physical training<br><br>COMPARISON:<br>mixed (i.e. inactive control group or sham training)	☐ Cognition<br><br>NR<br><br>☐ Brain / Neurochemicals / HRV<br><br>NR<br><br>☒ Physical Outcomes, Mobility, and Activities of Daily Living<br><br>HOA: fall rate** $RR^- = 0.79$, $p < 0.001$, 95 % CI [0.73 to 0.85], k = 69 ☐ sign. ☒ n.s.<br>mNCD: fall rate** $RR^- = 0.55$, $p = 0.004$, 95 % CI [0.37 to 0.83], k = 3 ☐ sign. ☒ n.s.<br><br>☐ Psychosocial Outcomes<br><br>NR
Kelly et al. 2014 [34]	POPULATION:<br>☒ healthy older adults<br>☐ older adults with mNCD<br>☐ older adults with Dementia<br><br>INTERVENTION:<br>☒ aerobic training<br>☒ resistance training<br>☐ balance training<br>☐ multicomponent physical training<br>☐ mixed physical training<br><br>COMPARISON:<br>(1) stretching/toning<br>(2) non- training active control (i.e. health education, watching movies, or socializing)<br>(3) no intervention	☒ Cognition<br><br>aerobic training<br>attention (1) $SMD^+ = 0.91$, $p = 0.15$, 95 % CI [- 0.34 to 2.16], k = 5, n = 409 ☒ sign. ☐ n.s.<br>delayed recall (1) $SMD^+ = 0.39$, $p = 0.16$, 95 % CI [- 0.15 to 0.82], k = 3, n = 278 ☒ sign. ☐ n.s.<br>work. memory (1) $SMD^+ = 0.28$, $p = 0.30$, 95 % CI [- 0.25 to 0.82], k = 2, n = 215 ☒ sign. ☐ n.s.<br>processing speed (1) $SMD^+ = 0.27$, $p = 0.28$, 95 % CI [- 0.22 to 0.75], k = 3, n = 307 ☒ sign. ☐ n.s.<br>immediate recall (1) $SMD^+ = 0.23$, $p = 0.62$, 95 % CI [- 0.67 to 1.14], k = 3, n = 307 ☒ sign. ☐ n.s.<br>recognition (2) $SMD^+ = 0.20$, $p = 0.51$, 95 % CI [- 0.39 to 0.78], k = 2, n = 93 ☐ sign. ☒ n.s.<br>overall cognition (2) $SMD^+ = 0.18$, $p = 0.26$, 95 % CI [- 0.13 to 0.49], k = 4, n = 432 ☐ sign. ☒ n.s.<br>verbal fluency (2) $SMD^+ = 0.09$, $p = 0.58$, 95 % CI [- 0.22 to 0.39], k = 3, n = 380 ☐ sign. ☒ n.s.<br>delayed recall (2) $SMD^+ = 0.04$, $p = 0.67$, 95 % CI [- 0.15 to 0.23], k = 4, n = 413 ☐ sign. ☒ n.s.<br>immediate recall (2) $SMD^+ = 0.00$, $p = 1.00$, 95 % CI [- 0.40 to 0.40], k = 3, n = 263 ☐ sign. ☒ n.s.<br>working memory (2) $SMD^+ = -0.03$, $p = 0.75$, 95 % CI [- 0.24 to 0.17], k = 5, n = 374 ☐ sign. ☒ n.s.<br>processing speed (2) $SMD^+ = -0.04$, $p = 0.76$, 95 % CI [- 0.29 to 0.21], k = 3, n = 413 ☐ sign. ☒ n.s.<br>attention (2) $SMD^+ = -0.06$, $p = 0.56$, 95 % CI [- 0.25 to 0.14], k = 6, n = 414 ☐ sign. ☒ n.s.<br>reasoning (2) $SMD^+ = -0.18$, $p = 0.28$, 95 % CI [- 0.49 to 0.14], k = 2, n = 210 ☐ sign. ☒ n.s.<br>reasoning (3) $SMD^+ = 0.47$, $p = 0.48$, 95 % CI [- 0.83 to 1.77], k = 2, n = 90 ☒ sign. ☐ n.s.<br>immediate recall (3) $SMD^+ = 0.29$, $p = 0.30$, 95 % CI [- 0.25 to 0.83], k = 2, n = 209 ☐ sign. ☒ n.s.<br>delayed recall (3) $SMD^+ = 0.27$, $p = 0.13$, 95 % CI [- 0.08 to 0.62], k = 3, n = 300 ☐ sign. ☒ n.s.<br>verbal fluency (3) $SMD^+ = 0.20$, $p = 0.14$, 95 % CI [- 0.07 to 0.48], k = 2, n = 209 ☐ sign. ☒ n.s.<br>working memory (3) $SMD^+ = 0.06$, $p = 0.70$, 95 % CI [- 0.24 to 0.35], k = 3, n = 181 ☐ sign. ☒ n.s.

Kelly et al.<br>2014 [34]<br>(continued)	POPULATION:<br>☒ healthy older adults<br>☐ older adults with mNCD<br>☐ older adults with Dementia<br><br>INTERVENTION:<br>☒ aerobic training<br>☒ resistance training<br>☐ balance training<br>☐ multicomponent physical training<br>☐ mixed physical training<br><br>COMPARISON:<br>(1) stretching/toning<br>(2) non- training active control (i.e. health education, watching movies, or socializing)<br>(3) no intervention	resistance training<br>reasoning (1)** SMD$^+$ = 3.16, p = 0.003, 95 % CI [1.07 to 5.24], k = 2, n = 135 ☐ sign. ☒ n.s.<br>working memory (1) SMD$^+$ = 0.10, p = 0.47, 95 % CI [- 0.17 to 0.36], k = 3, n = 236 ☐ sign. ☒ n.s.<br>attention (1) SMD$^+$ = -0.12, p = 0.37, 95 % CI [- 0.39 to 0.14], k = 3, n = 236 ☐ sign. ☒ n.s.<br>working memory (2) SMD$^+$ = 0.17, p = 0.31, 95 % CI [- 0.16 to 0.50], k = 2, n =152 ☐ sign. ☒ n.s.<br>attention (2) SMD$^+$ = -0.06, p = 0.62, 95 % CI [- 0.30 to 0.18], k = 3, n = 271 ☐ sign. ☒ n.s.<br><br>☐ Brain / Neurochemicals / HRV<br>NR<br><br>☐ Physical Outcomes, Mobility, and Activities of Daily Living<br>NR<br><br>☐ Psychosocial Outcomes<br>NR
Forbes et al.<br>2015 [35]	POPULATION:<br>☐ healthy older adults<br>☒ older adults with mNCD<br>☐ older adults with Dementia<br><br>INTERVENTION:<br>☐ aerobic training<br>☐ resistance training<br>☐ balance training<br>☐ multicomponent physical training<br>☒ mixed physical training<br><br>COMPARISON:<br>mixed (i.e. usual care/group activities)	☒ Cognition<br>global cognition SMD$^+$ = 0.43, p = 0.08, 95 % CI [- 0.05 to 0.92], k = 9, n = 409 ☒ sign. ☐ n.s. very low<br><br>☐ Brain / Neurochemicals / HRV<br>NR<br><br>☒ Physical Outcomes, Mobility, and Activities of Daily Living<br>ADL* SMD$^+$ = 0.68, p = 0.03, 95 % CI [0.08 to 1.27], k = 6, n = 289 ☒ sign. ☐ n.s. low<br><br>☒ Psychosocial Outcomes<br>depression SMD$^+$ = -0.14, p = 0.20, 95 % CI [-0.36 to 0.97], k = 5, n = 341 ☐ sign. ☒ n.s. moderate
Groot et al.<br>2015 [36]	POPULATION:<br>☐ healthy older adults<br>☒ older adults with mNCD<br>☐ older adults with Dementia<br><br>INTERVENTION:<br>☒ aerobic training<br>☒ resistance training<br>☐ balance training<br>☒ multicomponent physical training<br>☒ mixed physical training<br><br>COMPARISON:<br>non-exposed control condition or a control condition that received another intervention	☒ Cognition<br>pooled estimates<br>global cogn. (comb.execut.)** SMD$^+$ = 0.59, p < 0.01, 95 % CI [0.32 to 0.86], k = 6, n = 225 NR<br>global cogn. (pooled)** SMD$^+$ = 0.42, p < 0.01, 95 % CI [0.23 to 0.62], k = 16, n = 691 NR<br>global cogn. (aerobic)** SMD$^+$ = 0.41, p < 0.01, 95 % CI [0.05 to 0.76], k = 6, n = 320 NR<br>global cogn. (resistance) SMD$^+$ = - 0.10, p > 0.05, 95 % CI [- 0.38 to 0.19], k = 4, n = 191 NR<br>ADAS-cog (pooled)** SMD$^+$ = 0.38, p < 0.01, 95 % CI [0.09 to 0.66], k = 6, n = 210<br>Dementia NR<br>global cogn. (pooled)** SMD$^+$ = 0.47, p < 0.01, 95 % CI [0.14 to 0.80], k = 6, n = 248<br>NR<br><br>☐ Brain / Neurochemicals / HRV<br>NR<br><br>☒ Physical Outcomes, Mobility, and Activities of Daily Living<br>ADL (pooled)** SMD$^+$ = 1.18, p < 0.01, 95 % CI [0.57 to 1.79], k = 4 NR<br><br>☐ Psychosocial Outcomes<br>NR
Ströhle et al.<br>2015 [37]	POPULATION:<br>☐ healthy older adults<br>☒ older adults with mNCD<br>☐ older adults with Dementia<br><br>INTERVENTION:<br>☐ aerobic training<br>☐ resistance training<br>☐ balance training<br>☐ multicomponent physical training<br>☒ mixed physical training<br><br>COMPARISON:<br>non-exposed control condition or a control condition that received another intervention	☒ Cognition<br>mNCD<br>global cognition* SMCR$^+$ = 0.20, p < 0.05, 95 % CI [0.59 to 1.07], k = 6, n = 451 ☒ sign. ☐ n.s.<br>ADAS-cog* SMCR$^+$ = 0.83, p < 0.05, 95 % CI [0.11 to 0.28], k = 4, n = 119 ☒ sign. ☐ n.s.<br><br>☐ Brain / Neurochemicals / HRV<br>NR<br><br>☐ Physical Outcomes, Mobility, and Activities of Daily Living<br>NR<br><br>☐ Psychosocial Outcomes<br>NR

Table S3: Summary of meta-analytic results for simultaneous cognitive-motor training interventions
color-coding: black = significant effect, grey = no significant effect
* = significant at p ≤ 0.05, ** = significant at p ≤ 0.01

Abbreviations: ADL = Activities of Daily Living, BBS = Berg Balance Scale, COP = centre of pressure, FAS = F-A-S test, g = Hedge's g, GRADE = Grading of Recommendations Assessment, Development and Evaluation, IADL = Instrumeted Activities of Daily Living, HOA = healthy older adults, k = number of studies; mNCD = mild neurocognitive disorder, n = number of participants; SMD = standardized mean difference, TMT-A = Trail Making Test Part 1, TMT-B = Trail Making Test Part B, TUG = Timed Up and Go, VFT = Verbal Fluency test

Reference	Study Characteristics	Outcomes<br>Type | Statistics | Heterogeneity | GRADE
Corregidor-Sánchez et al. 2021 [38]	POPULATION:<br>☒ healthy older adults<br>☐ older adults with mNCD<br>☐ older adults with Dementia<br><br>INTERVENTION:<br>☐ sequential motor-cognitive<br>☐ coupled motor-cognitive<br>☒ exergame/VR/active videogame<br>☐ mixed cognitive-motor int.<br><br>COMPARISON:<br>active (i.e. physical training (six studies) and health education (three studies))	☐ Cognition<br><br>☐ Brain / Neurochemicals / HRV<br>NR<br><br>☒ Physical Outcomes, Mobility, and Activities of Daily Living<br>ADL — SMD^+ = 0.61, p = 0.11, 95 % CI [-0.15 to 1.37], k = 7, n = 192 — ☒ sign. ☐ n.s.<br>IADL — SMD^+ = - 0.34, p = 0.17, 95 % CI [- 0.82 to 0.15], k = 3, n = 67 — ☐ sign. ☒ n.s.<br>transfer — SMD^- = - 0.23, p = 0.35, 95 % CI [- 0.71 to 0.25], k = 6, n = 88 — ☐ sign. ☒ n.s.<br>ambulation** — SMD^- = - 0.63, p < 0.00001, 95 % CI [- 0.86 to - 0.40], k = 32, n = 1,315 — ☒ sign. ☐ n.s.<br><br>☐ Psychosocial Outcomes<br>NR
Gavelin et al. 2021 [39]	POPULATION:<br>☒ healthy older adults<br>☒ older adults with mNCD<br>☐ older adults with Dementia<br><br>INTERVENTION:<br>☒ sequential motor-cognitive<br>☒ coupled motor-cognitive<br>☒ exergame/VR/active videogame<br>☒ mixed cognitive-motor int.<br><br>COMPARISON:<br>active (i.e. combined intervention with physical training, cognitive training, a sham intervention (e.g. health education, relaxation, stretching or non-specific cognitive activ- ities such as data entry on a computer)), or passive (i.e. wait-list, no-contact).	☒ Cognition<br>pooled estimates:<br>global cognition* — g^+ = 0.30, p < 0.05, 95 % CI [0.06 to 0.53], k = 41 — NR<br>short-term memory* — g^+ = 0.30, p < 0.05, 95 % CI [0.05 to 0.55], k = 21 — NR<br>fluid reasoning* — g^+ = 0.24, p < 0.05, 95 % CI [0.02 to 0.46], k = 7 — NR<br>executive function* — g^+ = 0.22, p < 0.05, 95 % CI [0.13 to 0.30], k = 27 — NR<br>overall effect* — g^+ = 0.22, p < 0.05, 95 % CI [0.14 to 0.30], k = 41 — ☐ sign. ☒ n.s.<br>processing speed* — g^+ = 0.17, p < 0.05, 95 % CI [0.07 to 0.27], k = 22 — NR<br>visual processing — g^+ = 0.11, p > 0.05, 95 % CI [- 0.18 to 0.40], k = 6 — NR<br>HOA:<br>overall effect** — g^+ = 0.20, p < 0.001, 95 % CI [0.12 to 0.29], k = 28 — NR<br>mNCD:<br>overall effect* — g^+ = 0.26, p = 0.02, 95 % CI [0.06 to 0.46], k = 13 — NR<br><br>☐ Brain / Neurochemicals / HRV<br>NR<br><br>☒ Physical Outcomes, Mobility, and Activities of Daily Living<br>pooled estimates:<br>functional mobility* — g^+ = 0.34, p < 0.05, 95 % CI [0.16 to 0.52], k = 22 — NR<br>overall effect* — g^+ = 0.25, p < 0.05, 95 % CI [0.13 to 0.37], k = 32 — ☐ sign. ☒ n.s.<br>balance — g^+ = 0.23, p > 0.05, 95 % CI [-0.04 to 0.49], k = 12 — NR<br>cognitive-motor ability — g^+ = 0.19, p > 0.05, 95 % CI [-0.01 to 0.39], k = 14 — NR<br>strength — g^+ = 0.17, p > 0.05, 95 % CI [-0.10 to 0.44], k = 9 — NR<br>aerobic capacity — g^+ = 0.10, p > 0.05, 95 % CI [-0.06 to 0.26], k = 14 — NR<br>gait — g^+ = 0.06, p > 0.5, 95 % CI [-0.23 to 0.34], k = 10 — NR<br>HOA:<br>overall effect** — g^+ = 0.23, p = 0.005, 95 % CI [0.08 to 0.38], k = 24 — NR<br>mNCD:<br>overall effect** — g^+ = 0.32, p = 0.008, 95 % CI [0.12 to 0.52], k = 8 — NR<br><br>☒ Psychosocial Outcomes<br>pooled estimates:<br>overall effect* — g^+ = 0.10, p < 0.05, 95 % CI [0.01 to 0.20], k = 8 — ☐ sign. ☒ n.s

Biazus-Sehn et al. 2020 [19]	POPULATION:<br>☐ healthy older adults<br>☒ older adults with mNCD<br>☐ older adults with Dementia<br><br>INTERVENTION:<br>☐ sequential motor-cognitive<br>☒ coupled motor-cognitive<br>☐ exergame/VR/active videogame<br>☐ mixed cognitive-motor int.<br><br>COMPARISON:<br>mixed (i.e. no treatment; balance, tone or stretching programs; or social and/or mental activities)	☒ Cognition<br><br>delayed recall** SMD$^+$ = 0.593, p < 0.001, 95 % CI [0.269 to 0.917] NR<br>global cognition** SMD$^+$ = 0.531, p = 0.003, 95 % CI [0.172 to 0.529] NR<br>executive function* SMD$^+$ = 0.499, p = 0.015, 95 % CI [0.093 to 0.905] NR<br><br>☐ Brain / Neurochemicals / HRV<br><br>NR<br><br>☐ Physical Outcomes, Mobility, and Activities of Daily Living<br><br>NR<br><br>☐ Psychosocial Outcomes<br><br>NR
Chan et al. 2020 [40]	POPULATION:<br>☐ healthy older adults<br>☒ older adults with mNCD<br>☐ older adults with Dementia<br><br>INTERVENTION:<br>☐ sequential motor-cognitive<br>☒ coupled motor-cognitive<br>☐ exergame/VR/active videogame<br>☐ mixed cognitive-motor int.<br><br>COMPARISON:<br>mixed (i.e. waitlist control group, health education)	☒ Cognition<br><br>immediate recall** SMD$^+$ = 0.54, p < 0.001, 95 % CI [0.38 to 0.71], k = 4 ☐ sign. ☒ n.s.<br>global cognition** SMD$^+$ = 0.48, p < 0.001, 95 % CI [0.21 to 0.74], k = 4 ☐ sign. ☒ n.s.<br>attention** SMD$^+$ = 0.33, p < 0.01, 95 % CI [0.12 to 0.54], k = 4 ☐ sign. ☒ n.s.<br>delayed recall* SMD$^+$ = 0.33, p < 0.05, 95 % CI [0.01 to 0.64], k = 3 ☒ sign. ☐ n.s.<br>visuospatial skills* SMD$^+$ = 0.16, p < 0.05, 95 % CI [0.01 to 0.32], k = 4 ☐ sign. ☒ n.s.<br>language SMD$^+$ = 0.37, p > 0.05, 95 % CI [- 0.16 to 0.91], k = 2 ☐ sign. ☒ n.s.<br>mental flexibility SMD$^+$ = 0.18, p > 0.05, 95 % CI [- 0.13 to 0.49], k = 4 ☒ sign. ☐ n.s.<br>working memory SMD$^+$ = 0.11, p > 0.05, 95 % CI [- 0.18 to 0.40], k = 3 ☐ sign. ☒ n.s.<br><br>☐ Brain / Neurochemicals / HRV<br><br>☐ Physical Outcomes, Mobility, and Activities of Daily Living<br><br>☐ Psychosocial Outcomes
Chen et al. 2020 [20]	POPULATION:<br>☒ healthy older adults<br>☒ older adults with mNCD<br>☐ older adults with Dementia<br><br>INTERVENTION:<br>☐ sequential motor-cognitive<br>☒ coupled motor-cognitive<br>☐ exergame/VR/active videogame<br>☒ mixed cognitive-motor int.<br><br>COMPARISON:<br>mixed (no contact, no treatment, waiting list, sham training, and alternative active treatments for the comparison condition	☒ Cognition<br><br>pooled older adults:<br>overall effect (coupled)* g$^+$ = 0.44, p < 0.05, 95 % CI [0.29 to 0.60], k = 10 NR<br><br>☐ Brain / Neurochemicals / HRV<br><br>☐ Physical Outcomes, Mobility, and Activities of Daily Living<br><br>☐ Psychosocial Outcomes
Mansor et al. 2020 [3]	POPULATION:<br>☒ healthy older adults<br>☐ older adults with mNCD<br>☐ older adults with Dementia<br><br>INTERVENTION:<br>☐ sequential motor-cognitive<br>☐ coupled motor-cognitive<br>☐ exergame/VR/active videogame<br>☒ mixed cognitive-motor int.<br><br>COMPARISON:<br>mixed (passive (i.e. no-contact; k = 24) or active (e.g. watched a movie or a documentary, read a book, completed quizzes, or exercised; k = 9))	☒ Cognition<br><br>updating memory* g$^+$ = 0.37, p = 0.02, 95 % CI [0.07 to 0.66], k = 23, n = 879 ☒ sign. ☐ n.s.<br>inhibition* g$^+$ = 0.28, p = 0.02, 95 % CI [0.02 to 0.53], k = 20, n = 709 ☒ sign. ☐ n.s.<br>processing speed g$^+$ = 0.15, p = 0.20, 95 % CI [- 0.08 to 0.38], k = 21, n = 800 ☒ sign. ☐ n.s.<br>attention g$^+$ = 0.08, p = 0.77, 95 % CI [- 0.47 to 0.64], k = 11, n = 394 ☒ sign. ☐ n.s.<br>delayed memory g$^+$ = -0.03, p = 0.90, 95% CI [- 0.48 to 0.42], k = 10, n = 387 ☒ sign. ☐ n.s.<br>reasoning g$^+$ = 0.17, p = 0.45, 95 % CI [- 0.28 to 0.62], k = 13, n = 523 ☒ sign. ☐ n.s.<br>shifting g$^+$ = 0.14, p = 0.46,95 % CI [- 0.21 to 0.50], k = 18, n = 693 ☒ sign. ☐ n.s.<br><br>☐ Brain / Neurochemicals / HRV<br><br>NR<br><br>☐ Physical Outcomes, Mobility, and Activities of Daily Living<br><br>NR<br><br>☐ Psychosocial Outcomes<br><br>NR

Pacheco et al. 2020 [41]	POPULATION:<br>☒ healthy older adults<br>☐ older adults with mNCD<br>☐ older adults with Dementia<br><br>INTERVENTION:<br>☐ sequential motor-cognitive<br>☐ coupled motor-cognitive<br>☒ exergame/VR/active videogame<br>☐ mixed cognitive-motor int.<br><br>COMPARISON:<br>mixed (no intervention (e.g., no physical training) or to health education, or cognitive training without physical activity)	☐ Cognition<br>NR<br>☐ Brain / Neurochemicals / HRV<br>NR<br>☒ Physical Outcomes, Mobility, and Activities of Daily Living<br>balance (BBS)** MD^{+} = 2.46, p = 0.0001, 95 % CI [0.49 to 4.44], k = 3, n = 102 ☒ sign. ☐ n.s.<br>balance (COP sway)*" SMD^{-} = -0.93, p = 0.0001, 95 % CI [- 1.52 to - 0.34], k = 4, n = 124 ☒ sign. ☐ n.s.<br>mobility (TUG)** MD^{-} = -2.48, p = 0.0001, 95 % CI [- 3.83 to - 1.12], k = 3, n = 103 ☐ sign. ☒ n.s.<br>☐ Psychosocial Outcomes<br>NR
Zhu et al. 2020 [42]	POPULATION:<br>☐ healthy older adults<br>☒ older adults with mNCD<br>☐ older adults with Dementia<br><br>INTERVENTION:<br>☐ sequential motor-cognitive<br>☒ coupled motor-cognitive<br>☐ exergame/VR/active videogame<br>☐ mixed cognitive-motor int.<br><br>COMPARISON:<br>active (i.e. health education and/or training but not aerobic dance training)	☒ Cognition<br>global cognition** MD^{+} = 1.43, p = 0.0009, 95 % CI [0.59 to 2.27], k = 3, n = 524 ☐ sign. ☒ n.s.<br>verbal fluency (FAS)** MD^{+} = 1.73, p = 0.003, 95 % CI [0.58 to 2.88], k = 1, n = 129 not applicable<br>delayed recall** MD^{+} = 0.46, p < 0.00001, 95 % CI [0.30 to 0.62], k = 5, n = 647 ☐ sign. ☒ n.s.<br>immediate recall* MD^{+} = 0.24, p = 0.04, 95 % CI [0.01 to 0.46], k = 4, n = 513 ☐ sign. ☒ n.s.<br>verbal fluency (VFT) MD^{+} = 0.22, p = 0.9, 95 % CI [- 3.43 to 3.88], k = 2, n = 324 ☒ sign. ☐ n.s.<br>☐ Brain / Neurochemicals / HRV<br>NR<br>☐ Physical Outcomes, Mobility, and Activities of Daily Living<br>NR<br>☐ Psychosocial Outcomes<br>NR
Wang et al. 2019 [27]	POPULATION:<br>☐ healthy older adults<br>☒ older adults with mNCD<br>☐ older adults with Dementia<br><br>INTERVENTION:<br>☐ sequential motor-cognitive<br>☒ coupled motor-cognitive<br>☒ exergame/VR/active videogame<br>☐ mixed cognitive-motor int.<br><br>COMPARISON:<br>mixed (i.e. stretching, education, usual lifestyle, and social recreational activities)	☒ Cognition<br>global cogn. (combined)** SMD^{+} = 0.41, p < 0.001, 95 % CI [0.20 to 0.62], k = 7, n = 732 ☐ sign. ☒ n.s.<br>global cogn. (exergame) SMD^{+} = 0.57, p = 0.21, 95 % CI [-0.32 to 1.47], k = 1, n = 20 N/A<br>☐ Brain / Neurochemicals / HRV<br>☐ Physical Outcomes, Mobility, and Activities of Daily Living<br>☐ Psychosocial Outcomes
Wu et al. 2019 [43]	POPULATION:<br>☒ healthy older adults<br>☒ older adults with mNCD<br>☐ older adults with Dementia<br><br>INTERVENTION:<br>☐ sequential motor-cognitive<br>☒ coupled motor-cognitive<br>☐ exergame/VR/active videogame<br>☐ mixed cognitive-motor int.<br><br>COMPARISON:<br>mixed (i.e. active controls including aerobic training, memory training, and resistance training, or usual care)	☒ Cognition<br>pooled estimates:<br>global cogn. (dance)* MD^{+} = 1.12, p = 0.02, 95 % CI [0.16 to 2.08], k = 2, n = 263 ☐ sign. ☒ n.s.<br>global cogn. (tai chi)* MD^{+} = 0.97, p = 0.02, 95 % CI [0.19 to 1.76], k = 10, n = 1,604 ☒ sign. ☐ n.s.<br>global cogn. (pooled)** MD^{+} = 0.92, p = 0.002, 95 % CI [0.33 to 1.51], k = 13, n = 1,892 ☒ sign. ☐ n.s.<br>language fluency (dance)** MD^{+} = 0.61, p < 0.001, 95 % CI [0.09 to 0.45], k = 1, n = 129 N/A<br>work. memory (tai chi) MD^{+} = 0.46, p = 0.06, 95 % CI [- 0.03 to 0.94], k = 6, n = 837 ☒ sign. ☐ n.s.<br>work. memory (dance) MD^{+} = 0.41, p = 0.18, 95 % CI [- 0.19 to 1.00], k = 2, n = 92 ☐ sign. ☒ n.s.<br>memory (dance)* MD^{+} = 0.35, p = 0.003, 95 % CI [0.12 to 0.58], k = 3, n = 285 ☐ sign. ☒ n.s.<br>work. memory (pooled)* MD^{+} = 0.32, p = 0.05, 95 % CI [0.01 to 0.64], k = 10, n = 1,084 ☐ sign. ☒ n.s.<br>language fluency (pooled)* MD^{+} = 0.27, p = 0.003, 95 % CI [0.09 to 0.45], k = 9, n = 1,166 ☐ sign. ☒ n.s.<br>memory (pooled)** MD^{+} = 0.24, p = 0.001, 95 % CI [0.10 to 0.39], k = 15, n = 1,593 ☒ sign. ☐ n.s.<br>memory (tai chi)* MD^{+} = 0.20, p = 0.005, 95 % CI [0.06 to 0.35], k = 7, n = 888 ☐ sign. ☒ n.s.<br>language fluency (tai chi)* MD^{+} = 0.18, p = 0.05, 95 % CI [0.00 to 0.37], k = 6, n = 887 ☐ sign. ☒ n.s.<br>proc. speed (dance) MD^{-} = -0.69, p = 0.22, 95 % CI [- 1.78 to 0.41], k = 3, n = 222 ☐ sign. ☒ n.s.<br>proc. speed (tai chi) MD^{-} = -1.29, p = 0.74, 95 % CI [- 9.0 to 6.4], k = 4, n = 758 ☒ sign. ☐ n.s.<br>proc. speed (pooled) MD^{-} = -1.44, p = 0.39, 95 % CI [- 4.7 to 1.8], k = 9, n = 1,208 ☐ sign. ☒ n.s.<br>cogn. flexibility (dance) MD^{-} = -6.19, p = 0.38, 95 % CI [- 20.1 to 7.7], k = 3, n = 222 ☒ sign. ☐ n.s<br>cogn. flexibility (pooled)** MD^{-} = -8.80, p = 0.007, 95 % CI [- 15.2 to - 2.4], k = 13, n = 1,423 ☒ sign. ☐ n.s<br>cogn. flexibility (tai chi) MD^{-} = -9.06, p = 0.11, 95 % CI [- 20.0 to 1.9], k = 7, n = 894 ☒ sign. ☐ n.s<br>HOA:<br>global cogn. (pooled) MD^{+} = 0.57, p = 0.19, 95 % CI [- 0.28 to 141], k = 3, n = 705 ☒ sign. ☐ n.s.<br>mNCD:<br>global cogn. (pooled)** MD^{+} = 1.04, p = 0.008, 95 % CI [0.27 to 1.80], k = 10, n = 1,187 ☒ sign. ☐ n.s.<br>☐ Brain / Neurochemicals / HRV

		☐ Physical Outcomes, Mobility, and Activities of Daily Living<br><br>☐ Psychosocial Outcomes
Vaportzis et al. 2019 [7]	POPULATION:<br>☒ healthy older adults<br>☐ older adults with mNCD<br>☐ older adults with Dementia<br><br>INTERVENTION:<br>☐ sequential motor-cognitive<br>☐ coupled motor-cognitive<br>☐ exergame/VR/active videogame<br>☒ mixed cognitive-motor int.<br><br>COMPARISON:<br>(1) active control intervention (e.g. stretching or balance classes)<br>(2) wait-list control<br>(3) passive control (no intervention	☒ Cognition<br>TMT B (1) — SMD^+ = 0.16, p = 0.34, 95 % CI [-0.16 to 0.48], k = 2, n = 202 — ☐ sign. ☒ n.s.<br>TMT A (1) — SMD^+ = 0.10, p = 0.47, 95 % CI [-0.17 to 0.36], k = 4, n = 270 — ☐ sign. ☒ n.s.<br>digit symb. substitution(1) — SMD^+ = 0.07, p = 0.70, 95 % CI [-0.27 to 0.40], k = 2, n = 170 — ☐ sign. ☒ n.s.<br><br>☐ Brain / Neurochemicals / HRV<br><br>☐ Physical Outcomes, Mobility, and Activities of Daily Living<br><br>☐ Psychosocial Outcomes
Northey et al. 2018 [30]	POPULATION:<br>☒ healthy older adults<br>☒ older adults with mNCD<br>☐ older adults with Dementia<br><br>INTERVENTION:<br>☐ sequential motor-cognitive<br>☒ coupled motor-cognitive<br>☐ exergame/VR/active videogame<br>☐ mixed cognitive-motor int.<br><br>COMPARISON:<br>mixed (i.e. (1) sham training or alternative active treatment, (2) attention control, (3) no contact)	☒ Cognition<br>overall (tai chi)** — SMD^+ = 0.25, p < 0.01, 95 % CI [0.32 to 0.71], k = 47 — NR<br><br>☐ Brain / Neurochemicals / HRV<br><br>☐ Physical Outcomes, Mobility, and Activities of Daily Living<br><br>☐ Psychosocial Outcomes
Stanmore et al. 2017 [44]	POPULATION:<br>☒ healthy older adults<br>☒ older adults with mNCD<br>☐ older adults with Dementia<br><br>INTERVENTION:<br>☐ sequential motor-cognitive<br>☒ coupled motor-cognitive<br>☒ exergame/VR/active videogame<br>☐ mixed cognitive-motor int.<br><br>COMPARISON:<br>(1) physically active (e.g. aerobic training, balance, stretching, strenth training)<br>(2) cognitively active (e.g. health education sessions, reading, cognitive remediation, or memory training)<br>(3) passive (e.g. waitlist/usual treatment)	☒ Cognition<br>pooled estimates:<br>spat. learning / memory — g^+ = 1.230, p = 0.264, 95 % CI [- 0.93 to 3.39], k = 3, n = 135 — ☒ sign. ☐ n.s.<br>inhibitory control** — g^+ = 0.900, p < 0.001, 95 % CI [0.48 to 1.33], k = 5, n = 139 — ☐ sign. ☒ n.s.<br>language — g^+ = 0.570, p = 0.360, 95 % CI [- 0.65 to 1.79], k = 3, n = 184 — ☒ sign. ☐ n.s.<br>verb. learning / memory — g^+ = 0.525, p = 0.085, 95 % CI [- 0.07 to 1.13], k = 4, n = 171 — ☒ sign. ☐ n.s.<br>global cognition (1 - 3)** — g^+ = 0.436, p = 0.001, 95 % CI [0.18 to 0.69], k = 17, n = 926 — ☒ sign. ☐ n.s.<br>global cognition (1)* — g^+ = 0.435, p = 0.030, 95 % CI [0.04 to 0.83], k = 8, n = 632 — ☒ sign. ☐ n.s.<br>reasoning — g^+ = 0.393, p = 0.495, 95 % CI [- 0.74 to 1.52], k = 3, n = 134 — ☒ sign. ☐ n.s.<br>global cognition (1 & 2)* — g^+ = 0.363, p = 0.020, 95 % CI [0.06 to 0.67], k = 12, n = 768 — ☒ sign. ☐ n.s.<br>cognitive flexibility* — g^+ = 0.348, p = 0.049, 95 % CI [0.002 to 0.694], k = 8, n = 245 — ☒ sign. ☐ n.s.<br>visuo-spatial skills * — g^+ = 0.345, p = 0.033, 95 % CI [0.03 to 0.66], k = 4, n = 226 — ☐ sign. ☒ n.s.<br>attention / proc. speed* — g^+ = 0.298, p = 0.027, 95 % CI [0.03 to 0.56], k = 11, n = 688 — ☒ sign. ☐ n.s.<br>Executive Function* — g^+ = 0.256, p = 0.048, 95 % CI [0.002 to 0.510], k = 13, n = 745 — ☒ sign. ☐ n.s.<br>working memory — g^+ = 0.032, p = 0.831, 95 % CI [- 0.26 to 0.33], k = 4, n = 171 — ☐ sign. ☒ n.s.<br>HOA:<br>global cognition (1 - 3)** — g^+ = 0.573, p = 0.010, 95 % CI [0.14 to 1.01], k = 9, n = 415 — ☒ sign. ☐ n.s.<br>mNCD (mixed):<br>global cognition (1 - 3)* — g^+ = 0.340, p = 0.017, 95 % CI [0.06 to 0.62], k = 6, n = 193 — ☐ sign. ☒ n.s.<br><br>☐ Brain / Neurochemicals / HRV<br><br>☐ Physical Outcomes, Mobility, and Activities of Daily Living<br><br>☐ Psychosocial Outcomes

Howes et al. 2017 [45]	POPULATION:<br>☒ healthy older adults<br>☐ older adults with mNCD<br>☐ older adults with Dementia<br><br>INTERVENTION:<br>☐ sequential motor-cognitive<br>☐ coupled motor-cognitive<br>☒ exergame/VR/active videogame<br>☐ mixed cognitive-motor int.<br><br>COMPARISON:<br>(1) active control<br>(2) inactive control	☒ Cognition<br><br>executive function (1 & 2)* g^- = - 0.48, p = 0.003, 95 % CI [- 0.80 to - 0.17], k = 8, n = 459 ☒ sign. ☐ n.s low<br>executive function (1)** g^- = - 0.65, p < 0.001, 95 % CI [- 1.03 to - 0.28], k = 3, n = 144 ☐ sign. ☒ n.s low<br>executive function (2)* g^- = - 0.40, p = 0.05, 95 % CI [- 0.79 to - 0.00], k = 5, n = 315 ☒ sign. ☐ n.s low<br><br>☐ Brain / Neurochemicals / HRV<br><br>☒ Physical Outcomes, Mobility, and Activities of Daily Living<br><br>balance (1 & 2)* g^+ = 0.52, p < 0.05, 95 % CI [0.24 to 0.79], k = 17, n = 743 NR low<br>balance (1)* g^+ = 0.56, p < 0.05, 95 % CI [0.25 to 0.87], k = 10, n = 394 NR very low<br>balance (2)* g^+ = 0.51, p < 0.05, 95 % CI [0.02 to 1.01], k = 7, n = 349 NR very low<br>funct. exercise cap. (1 & 2)* g^+ = 0.29, p < 0.05, 95 % CI [0.04 to 0.55], k = 7, n = 248 NR low<br>funct. exercise cap. (1)* g^+ = 0.58, p < 0.05, 95 % CI [0.09 to 1.07], k = 3, n = 70 NR very low<br>funct. exercise cap. (2) g^+ = 0.19, p > 0.05, 95 % CI [- 0.11 to 0.48], k = 4, n = 178 NR very low<br>functional mobility (1 & 2) g^- = -0.36, p > 0.05, 95 % CI [- 0.36 to 0.09], k = 16, n = 670 NR very low<br>functional mobility (1) g^- = -0.12, p > 0.05, 95 % CI [- 0.48 to 0.25], k = 6, n = 260 NR very low<br>functional mobility (2) g^- = -0.14, p > 0.05, 95 % CI [- 0.45 to 0.17], k = 10, n = 410 NR very low<br><br>☒ Psychosocial Outcomes<br><br>fear of falling (1 & 2) g^+ = 0.18, p > 0.05, 95 % CI [- 0.16 to 0.53], k = 16, n = 816 NR very low<br>fear of falling (1) g^+ = 0.28, p > 0.05, 95 % CI [- 0.50 to 1.05], k = 8, n = 325 NR very low<br>fear of falling (2) g^+ = 0.10, p > 0.05, 95 % CI [- 0.09 to 0.29], k = 8, n = 491 NR low

Table S4: Synthesis of Evidence for Moderating Effects of Training Parameters in Older Adults with mNCD or Dementia based on Meta-Analyses
color-coding: black = significant effect, grey = no significant effect
* = significant at p ≤ 0.05, ** = significant at p ≤ 0.01
↑ = significant improvement, ↗ trend for improvement, → no significant effect

Abbreviations: DUAL = simultaneous cognitive-motor training, COG = cognitive training, g = Hedge's g, k = number of studies; MD = mean difference (absolute), mNCD = mild neurocognitive disorder, n = number of participants; NR = not reported, PHYS = physical training, SMD = standardized mean difference, SE = standard error

Parameter	Moderating Effects					
	Outcome	Reference	Type of Training	Variable & Relation		Statistics
Frequency	☒ Cognition					
	Older Adults with mNCD/Dementia:					
	Global Cognition:					
	subgroup analysis	Bahar-Fuchs et al. 2019 [5]	☒ COG ☐ PHYS ☐ DUAL	pooled estimate**		SMD^+ = 0.42, p < 0.0001, 95 % CI [0.23 to 0.61], k = 27
				≤ 3x/week*		SMD^+ = 0.33, p = 0, 95 % CI [0.13 to 0.53], k = 20
				> 3x/week*		SMD^+ = 0.71, p = 0, 95 % CI [0.27 to 1.14], k = 7
				between-group diff.	↗	Chi^2 = 2.39, p = 0.12, I^2 = 58.13 %
	moderator analysis	Sanders et al. 2019 [26]	☐ COG ☒ PHYS ☐ DUAL	moderator effect*	↑*	F(2, 51) = 1.589, p < 0.05
				pooled estimate*		g^+ = 0.22, p < 0.05, 95 % CI [0.05 to 0.39], k = 13
				2x/week		g^+ = 0.05, p > 0.05, 95 % CI [-0.15 to 0.26]
				3x/week*		g^+ = 0.35, p < 0.05, 95 % CI [0.04 to 0.66]
				post-hoc test$_{reference = 2x/week}$	→	ß = 0.22, 95 % CI [-0.16 to 0.61], p > 0.05
				4x/week**		g^+ = 0.50, p < 0.01, 95 % CI [0.24 to 0.76]
				post-hoc test$_{reference = 2x/week}$*	↑*	ß = 0.42, 95 % CI [0.06 to 0.78], p < 0.05
	subgroup analysis	Groot et al. 2015 [36]	☐ COG ☒ PHYS ☐ DUAL	pooled estimate**		SMD^+ = 0.42, p < 0.01, 95 % CI [0.23 to 0.62], k = 16
				multicomponent$_{pooled}$ (1)**		SMD^+ = 0.59, p < 0.01, 95 % CI [0.32 to 0.86], k = 6
				aerobic only$_{pooled}$ (2)**		SMD^+ = 0.41, p < 0.01, 95 % CI [0.05 to 0.76], k = 6
				resistance only$_{pooled}$ (3)		SMD^+ = -0.10, p > 0.05, 95 % CI [-0.38 to 0.19], k = 4
				low frequency (1-3)*		SMD^+ = 0.33, p < 0.05, 95 % CI [0.03 to 0.63], k = 7
				high frequency (1-3)**		SMD^+ = 0.64, p < 0.01, 95 % CI [0.39 to 0.89], k = 7
				between-group diff. (1-3)**	↓**	t(12) = 4.02, p < 0.01
				low frequency (1 + 2)**		SMD^+ = 0.68, p < 0.01, 95 % CI [0.39 to 0.96], k = 5
				high frequency (1 + 2)*		SMD^+ = 0.47, p < 0.05, 95 % CI [0.06 to 0.88], k = 5
				between-group diff. (1 + 2)	→	t(8) = 1.79, p = 0.11
	Executive Function:					
	subgroup analysis	Bahar-Fuchs et al. 2019 [5]	☒ COG ☐ PHYS ☐ DUAL	pooled estimate**		SMD^+ = 0.75, p = 0, 95 % CI [0.28 to 1.22], k = 12
				≤ 3x/week*		SMD^+ = 0.57, p = 0.04, 95 % CI [0.01 to 1.13], k = 8
				> 3x/week*		SMD^+ = 1.20, p = 0.02, 95 % CI [0.2 to 2.20], k = 4
				between-group diff.	→	Chi^2 = 1.14, p = 0.29, I^2 = 12.19 %
	Learning and Memory:					
	subgroup analysis	Bahar-Fuchs et al. 2019 [5]	☒ COG ☐ PHYS ☐ DUAL	immediate memory:		
				pooled estimate**		SMD^+ = 0.74, p < 0.0001, 95 % CI [0.29 to 1.19], k = 18
				≤ 3x/week**		SMD^+ = 0.73, p = 0, 95 % CI [0.27 to 1.19], k = 14
				> 3x/week**		SMD^+ = 0.74, p = 0, 95 % CI [0.29 to 1.19], k = 4
				between-group diff.	→	Chi^2 = 0, p = 0.97, I^2 = 0 %
	Language:					
	subgroup analysis	Bahar-Fuchs et al. 2019 [5]	☒ COG ☐ PHYS ☐ DUAL	pooled estimate		SMD^+ = 0.22, p = 0.13, 95 % CI [- 0.07 to 0.50], k = 12
				≤ 3x/week		SMD^+ = 0.05, p = 0.57, 95 % CI [- 0.13 to 0.24], k = 9
				> 3x/week*		SMD^+ = 1.00, p = 0.03, 95 % CI [0.09 to 1.92], k = 3
				between-group diff.	→	Chi^2 = 3.96, p = 0.05, I^2 = 74.72 %
	Mixed Healthy Older Adults and Older Adults with mNCD or Dementia:					
	Global Cognition:					
	bivariate correlation	Gomes-Osman et al. 2018 [46]	☐ COG ☒ PHYS ☐ DUAL	frequency (per week)	→	r = -0.05, p = 0.67
	Healthy Older Adults:					
	Global Cognition:					
	moderator analysis	Sanders et al. 2019 [26]	☐ COG ☒ PHYS ☐ DUAL	moderator effect	→	F(3, 128) = 0.283, p > 0.05
				pooled estimate**		g^+ = 0.26, p < 0.01, 95 % CI [0.15 to 0.37], k = 23
				1x/week		g^+ = 0.23, p > 0.05, 95 % CI [-0.17 to 0.63]
				2x/week*		g^+ = 0.34, p < 0.05, 95 % CI [0.07 to 0.62]
				3x/week**		g^+ = 0.23, p < 0.01, 95 % CI [0.09 to 0.37]
				4x/week		g^+ = 0.41, p > 0.05, 95 % CI [-0.19 to 1.00]
	subgroup analysis	Jia et al. 2019 [23]	☐ COG ☒ PHYS ☐ DUAL	pooled estimate**		SMD^+ = 1.12, p < 0.001, 95 % CI [0.66 to 1.59], k = 13
				up to 3x/week*		SMD^+ = 1.58, 95 % CI [1.01 to 2.14], k = 3
				> 3x/week*		SMD^+ = 0.99, 95 % CI [0.49 to 1.50], k = 10
				between-group difference	→	NR
	moderator analysis	Northey et al. 2018 [30]	☐ COG ☒ PHYS ☒ DUAL	moderator effect**	↑**	Q_5 = 24.12, p < 0.01
				pooled estimate**		SMD^+ = 0.29, p < 0.01, 95 % CI [0.17 to 0.41], k = 36
				< 2x/week*		SMD^+ = 0.32, p < 0.05, 95 % CI [0.13 to 0.52], n = 92
				3 - 4x/week*		SMD^+ = 0.24, p < 0.05, 95 % CI [0.07 to 0.52], n = 229

Frequency (continued)				5 - 7x/week*		SMD$^+$ = 0.69, p < 0.05, 95 % CI [0.10 to 1.28], n = 13
	moderator analysis	Mewborn et al. 2017 [13]	☒ COG ☐ PHYS ☐ DUAL	moderator effect**	↓**	Q = 816.39, p = 0.001
				pooled estimate**		g^+ = 0.298, p < 0.001, 95 % CI [0.25 to 0.35], k = 279
				3+ times/week**		g^+ = 0.304, p < 0.001, 95 % CI [0.22 to 0.39], k = 122
				1 – 2 times/week**		g^+ = 0.363, p < 0.001, 95 % CI [0.29 to 0.44], k = 144
	subgroup analysis	Lampit et al. 2014 [17]	☒ COG ☐ PHYS ☐ DUAL	pooled estimate**		g^+ = 0.22, p < 0.001, 95 % CI [0.15 to 0.29], k = 52
				1x/week*		g^+ = 0.34, p < 0.05, 95 % CI [0.16 to 0.51], k = 5
				2 – 3x/week*		g^+ = 0.28, p < 0.05, 95 % CI [0.18 to 0.37], k = 29
				> 3x/week		g^+ = 0.07, p > 0.05, 95 % CI [-0.05 to 0.19], k = 16
				between-group diff.**	↓**	p = 0.01
	Attention:					
	correlation	Mansor et al. 2020 [3]	☐ COG ☐ PHYS ☒ DUAL	sessions per week	→	coefficient (SE) = -0.05 (0.20), p = 0.81
	Executive Function:					
	moderator analysis	Chen et al. 2020 [20]	☐ COG ☒ PHYS ☒ DUAL	frequency (weeks)*	↑*	Q(1) = 10.86, p < 0.05
				low (1 – 2 times)*		g^+ = 0.15, p < 0.05, 95 % CI [0.08 to 0.22], k = 46
				moderate (3 – 4 times)*		g^+ = 0.31, p < 0.05, 95 % CI [0.24 to 0.38], k = 56
	Inhibition:					
	correlation	Mansor et al. 2020 [3]	☐ COG ☐ PHYS ☒ DUAL	sessions per week	→	coefficient (SE) = -0.10 (0.12), p = 0.40
	Shifting:					
	correlation	Mansor et al. 2020 [3]	☐ COG ☐ PHYS ☒ DUAL	sessions per week	→	coefficient (SE) = -0.05 (0.18), p = 0.79
	Updating:					
	correlation	Mansor et al. 2020 [3]	☐ COG ☐ PHYS ☒ DUAL	sessions per week	→	coefficient (SE) = -0.11 (0.14), p = 0.44
	Memory:					
	correlation	Mansor et al. 2020 [3]	☐ COG ☐ PHYS ☒ DUAL	sessions per week	→	coefficient (SE) = 0.04 (0.19), p = 0.85
	Processing Speed:					
	correlation	Mansor et al. 2020 [3]	☐ COG ☐ PHYS ☒ DUAL	sessions per week	→	coefficient (SE) = -0.01 (0.11), p = 0.89
	Reasoning:					
	correlation	Mansor et al. 2020 [3]	☐ COG ☐ PHYS ☒ DUAL	sessions per week	→	coefficient (SE) = 0.10 (0.22), p = 0.66
	☒ Brain / Neurochemicals / HRV					
	Heart Rate Variability:					
	ST-SDNN					
	meta-regression	Raffin et al. 2019 [25]	☐ COG ☒ PHYS ☐ DUAL	frequency/week*	↑*	coefficient (SE) = 0.191 (0.096), p = 0.046
	RMSSD					
	meta-regression	Raffin et al. 2019 [25]	☐ COG ☒ PHYS ☐ DUAL	frequency/week	→	coefficient (SE) = -0.008 (0.130), p = 0.928
	HF-HRV					
	meta-regression	Raffin et al. 2019 [25]	☐ COG ☒ PHYS ☐ DUAL	frequency/week	→	coefficient (SE) = 0.111 (0.081), p = 0.174
	☐ Physical Outcomes, Mobility, and Activities of Daily Living					
	☐ Psychosocial Outcomes					
Intensity / Complexity	☒ Cognition					
	Older Adults with mNCD/Dementia:					
	Global Cognition:					
	subgroup analysis	Law et al. 2020 [22]	☐ COG ☒ PHYS ☐ DUAL	aerobic training (pooled)**		SMD$^+$ = 0.45, 95 % CI [0.15 to 0.76], k = 11
				mod. to high intensity**		SMD$^+$ = 0.60, 95 % CI [0.21 to 0.98], k = 7
				between-group difference	→	NR
	moderator analysis	Sanders et al. 2019 [26]	☐ COG ☒ PHYS ☐ DUAL	moderator effect	→	F(1, 25) = 1.967, p > 0.05, and F(2, 6) = 0.845, p > 0.05
				aerobic training (pooled)		g^+ = 0.22, p > 0.05, 95 % CI [-0.20 to 0.64]
				low intensity		g^+ = 0.42, p > 0.05, 95 % CI [-0.10 to 0.93]
				moderate intensity		g^+ = 0.06, p > 0.05, 95 % CI [-0.12 to 0.24]
				multicomp. ex. (pooled)**		g^+ = 0.36, p < 0.01, 95 % CI [0.04 to 0.68]
				low intensity		g^+ = 1.02, p > 0.05, 95 % CI [-0.35 to 2.39]
				moderate intensity		g^+ = 0.21, p > 0.05, 95 % CI [-0.47 to 0.89]
				high intensity		g^+ = 0.42, p > 0.05, 95 % CI [-0.80 to 1.64]
	Executive Function:					
	moderator analysis	Chen et al. 2020 [20]	☐ COG ☒ PHYS ☒ DUAL	intensity	→	Q(1) = 2.87, p > 0.05
				moderate*		g^+ = 0.11, p < 0.05, 95 % CI [0.00 to 0.21], k = 25
				vigorous*		g^+ = 0.22, p < 0.05, 95 % CI [0.14 to 0.30], k = 44
	subgroup-analysis	Biazus-Sehn et al. 2020 [19]	☐ COG ☒ PHYS ☒ DUAL	pooled effect*		SMD$^+$ = 0.21, p = 0.026, 95 % CI [0.03 to 0.40], k = 19
				light intensity		SMD$^+$ = 0.04, p = 0.928, 95 % CI [-0.82 to 0.90]
				moderate intensity*	↑*	SMD$^+$ = 0.45, p = 0.039, 95 % CI [0.08 to 0.82]
				vigorous intensity		SMD$^+$ = 0.07, p = 0.688, 95 % CI [-0.26 to 0.39]
	Working Memory:					
	subgroup-analysis	Biazus-Sehn et al. 2020 [19]		pooled effect		SMD$^+$ = -0.01, p = 0.939, 95 % CI [-0.03 to 0.30], k = 15
				moderate intensity*	↑*	SMD$^+$ = -0.06, p = 0.039, 95 % CI [-1.26 to -0.03]
	Memory:					

Intensity / Complexity (continued)	subgroup-analysis	Biazus-Sehn et al. 2020 [19]	☐ COG ☒ PHYS ☒ DUAL	pooled effect*		SMD^{+} = 0.18, p = 0.047, 95 % CI [0.00 to 0.36], k = 17
				light intensity		SMD^{+} = 0.26, p = 0.244, 95 % CI [-0.18 to 0.70]
				moderate intensity*	↑*	SMD^{+} = 0.66, p = 0.002, 95 % CI [0.023 to 1.09]
				vigorous intensity		SMD^{+} = -0.01, p = 0.930, 95 % CI [-0.35 to 0.32]
	**Healthy Older Adults:**					
	Global cognition:					
	moderator analysis	Sanders et al. 2019 [26]	☐ COG ☒ PHYS ☐ DUAL	moderator effect	→	F(2, 77) = 2.396, p > 0.05 AND F(2, 40) = 0.243, p > 0.05
				aerobic exerc. (pooled)**		g^{+} = 0.22, p < 0.01, 95 % CI [0.03 to 0.41]
				low intensity		g^{+} = 0.65, p > 0.05, 95 % CI [-0.32 to 1.62]
				moderate intensity**		g^{+} = 0.25, p < 0.01, 95 % CI [0.07 to 0.43]
				high intensity		g^{+} = -0.30, p > 0.05, 95 % CI [-0.94 to 0.34]
				multicomp. ex. (pooled)		g^{+} = 0.30, p > 0.05, 95 % CI [-0.01 to 0.60]
				low intensity		g^{+} = 0.03, p > 0.05, 95 % CI [-1.77 to 1.84]
				moderate intensity		g^{+} = 0.24, p > 0.05, 95 % CI [-1.57 to 2.04]
				high intensity		g^{+} = 0.49, p > 0.05, 95 % CI [-0.80 to 1.79]
	moderator analysis	Northey et al. 2018 [30]	☐ COG ☒ PHYS ☐ DUAL	moderator effect	↑**	Q_5 = 13.55, p < 0.01
				aerobic exerc. (pooled)**		SMD^{+} = 0.29, p < 0.01, 95 % CI [0.17 to 0.41], k = 36
				low intensity		SMD^{+} = 0.10, p > 0.05, 95 % CI [-0.02 to 0.23], n = 71
				moderate intensity		SMD^{+} = 0.17, p < 0.05, 95 % CI [0.03 to 0.33], n = 57
				high intensity		SMD^{+} = 0.16, p < 0.05, 95 % CI [0.04 to 0.27], n = 8
	moderator analysis	Toril et al. 2014 [16]	☒ COG ☐ PHYS ☐ DUAL	moderator effect	→	Q(1) = 0.55, p > 0.05
				pooled estimate**		d^{+} = 0.37, p < 0.01, 95 % CI [0.26 to 0.48
				simple games**		d^{+} = 0.42, p < 0.01, 95 % CI [0.25 to 0.58]
				complex games**		d^{+} = 0.33, p < 0.01, 95 % CI [0.18 to 0.48]
	☐ **Brain / Neurochemicals / HRV**					
	☒ **Physical Outcomes, Mobility, and Activities of Daily Living**					
	**Healthy Older Adults:**					
	Fall Rate:					
	meta-regression	Sherrington et al. 2017 [33]	☐ COG ☒ PHYS ☒ DUAL	resistance training		
				moderate-high intensity	→	RR^{-} (95 % CI) = 0.97 (0.82 to 1.15), p = 0.73, ΔI^2 = - 4 %
				high-intensity	→	RR^{-} (95 % CI) = 1.23 (0.96 to 1.57), p = 0.11, ΔI^2 = 9 %
				balance training		
				moderate-high challenge	→	RR^{-} (95 % CI) = 0.85 (0.71 to 1.00), p = 0.06, ΔI^2 = 19 %
				high challenge*	↑*	RR^{-} (95 % CI) = 0.85 (0.73 to 1.00), p = 0.04, ΔI^2 = 28 %
	☐ **Psychosocial Outcomes**					
Type (of training)	☒ **Cognition**					
	**Older Adults with mNCD/Dementia:**					
	Global Cognition:					
	network meta-analysis	Gavelin et al. 2021 [39]	☒ COG ☒ PHYS ☒ DUAL	pairwise comparisons		see table 4 of publication
				pooled estimate*		g^{+} = 0.26, p = 0.02, 95 % CI [0.06 to 0.46], k = 13
				simultaneous training*		g^{+} = 0.45, p < 0.05, 95 % CI [0.11 to 0.78], k = 3
				sequential training		g^{+} = 0.25, p > 0.05, 95 % CI [-0.05 to 0.55], k = 2
				cognitive training		g^{+} = 0.26, p > 0.05, 95 % CI [-0.12 to 0.64], k = 2
				exergaming		g^{+} = 0.13, p > 0.05, 95 % CI [-0.22 to 0.48], k = 2
				physical training		g^{+} = 0.07, p > 0.05, 95 % CI [-0.26 to 0.39], k = 2
	subgroup analysis	Biazus-Sehn et al. 2020 [19]	☐ COG ☒ PHYS ☒ DUAL	pooled estimate**		SMD^{+} = 0.35, p < 0.001, 95 % CI [0.17 to 0.43], k = 18
				aerobic training only		SMD^{+} = 0.19, p = 0.364, 95 % CI [-0.22 to 0.59]
				resistance training only		SMD^{+} = 0.19, p = 0.488, 95 % CI [-0.35 to 0.74]
				multicomp. training only		SMD^{+} = 0.38, p = 0.066, 95 % CI [-0.03 to 0.79]
				comb. cognitive-motor**	↑**	SMD^{+} = 0.53, p = 0.003, 95 % CI [0.17 to 0.53]
				between-group difference		NR
	subgroup analysis	Law et al. 2020 [22]	☐ COG ☒ PHYS ☐ DUAL	pooled estimate**		SMD^{+} = 0.42, p < 0.001, 95 % CI [0.25 to 0.59], k = 26
				aerobic training only**		SMD^{+} = 0.45, 95 % CI [0.15 to 0.76], k = 11, n = 704
				multicomp. exerc. only**		SMD^{+} = 0.43, 95 % CI [0.13 to 0.73], k = 9, n = 831
				between-group difference		NR
	subgroup analysis	Bahar-Fuchs et al. 2019 [5]	☒ COG ☐ PHYS ☐ DUAL	pooled estimate*		SMD^{+} = 0.42, p < 0.001, 95 % CI [0.23 to 0.61], k = 27
				traditional training*		SMD^{+} = 0.43, p = 0, 95 % CI [0.18 to 0.68], k = 18
				augmented training*		SMD^{+} = 0.37, p = 0.01, 95 % CI [0.10 to 0.65], k = 9
				between-group difference		Chi^2 = 0.09, p = 0.77, I^2 = 0 %
	subgroup analysis	Wu et al. 2019 [43]	☐ COG ☐ PHYS ☒ DUAL	pooled estimate**		MD^{+} = 0.92, p = 0.002, 95 % CI [0.33 to 1.51], k = 13
				dance only*		MD^{+} = 1.12, p = 0.02, 95 % CI [0.16 to 2.08], k = 2
				tai chi only*	→	MD^{+} = 0.97, p = 0.02, 95 % CI [0.19 to 1.76], k = 10
				between-group difference		p = 0.25, I^2 = 59 %
	moderator analysis	Sanders et al. 2019 [26]	☐ COG ☒ PHYS ☐ DUAL	moderator effect	→	F(2, 51) = 0.432, p > 0.05
				pooled estimate*		g^{+} = 0.22, p < 0.05, 95 % CI [0.05 to 0.39], k = 13
				aerobic only		g^{+} = 0.22, p > 0.05, 95 % CI [-0.20 to 0.64]

Type (of training; continued)						
				resistance only		g^+ = 0.22, p > 0.05, 95 % CI [-0.20 to 0.64]
				multicomponent only**		g^+ = 0.36, p < 0.01, 95 % CI [0.04 to 0.68]
				between-group difference	→	F(3, 182) = 0.322, p > 0.05
	subgroup analysis	Panza et al. 2018 [31]	☐ COG ☒ PHYS ☐ DUAL	pooled estimate**		d^+ = 0.47, p < 0.05, 95 % CI [0.26 to 0.68], k = 23
				aerobic training only*		d^+ = 0.65, p < 0.05, 95 % CI [0.35 to 0.95], k = 15
				multicomp. training only		d^+ = 0.19, p > 0.05, 95 % CI [-0.06 to 0.43], k = 8
				between group difference	→	p = 0.11
	subgroup analysis	Liang et al. 2018 [9]	☒ COG ☒ PHYS ☐ DUAL	pooled estimate		SMD^+ = 0.13, p = 0.212, 95 % CI [-0.00 to 0.25], k = 15
				aerobic training only		SMD^+ = 0.35, p = 0.051, 95 % CI [-0.12 to 0.82], k = 4
				cognitive training only		SMD^+ = -0.02, p = 0.588, 95 % CI [-0.31 to 0.27], k = 4
				between-group difference		NR
	direct comparison	García-Casal et al. 2017 [11]	☒ COG ☐ PHYS ☐ DUAL	computer based vs. not	↑*	SMD^+ = 0.48, p = 0.02, 95 % CI [0.09 to 0.87], n = 119
	subgroup analysis	Shermann et al. 2017 [14]	☒ COG ☐ PHYS ☐ DUAL	pooled estimate		g^+ = 0.445, p = 0.002, 95 % CI [0.072 to 0.730], k = 26
				restorative training		g^+ = 0.389, p = 0.156, 95 % CI [-0.149 to 0.927], k = 8
				compensatory training		g^+ = 0.623, p = 0.150, 95 % CI [-0.224 to 1.470], k = 3
				multidomain training*		g^+ = 0.438, p = 0.019, 95 % CI [0.072 to 0.804], k = 15
				between-group difference		Q = 0.211, p = 0.900
				group training		g^+ = 0.297, p = 0.158, 95 % CI [-0.116 to 0.710], k = 12
				individual training*		g^+ = 1.008, p = 0.006, 95 % CI [0.293 to 1.723], k = 4
				computer training		g^+ = 0.394, p = 0.098, 95 % CI [-0.072 to 0.859], k = 10
				between-group difference		Q = 2.918, p = 0.232
	subgroup analysis	Groot et al. 2015 [36]	☐ COG ☒ PHYS ☐ DUAL	pooled estimate**		SMD^+ = 0.42, p < 0.01, 95 % CI [0.23 to 0.62], k = 16
				multicomponent (1)**		SMD^+ = 0.59, p < 0.01, 95 % CI [0.32 to 0.86], k = 6
				aerobic only (2)**		SMD^+ = 0.41, p < 0.01, 95 % CI [0.05 to 0.76], k = 6
				resistance only (3)		SMD^+ = -0.10, p > 0.05, 95 % CI [-0.38 to 0.19], k = 4
				subgroup diff. (1+2 vs 3)**	↑**	t(14) = 9.08, p < 0.01
				subgroup diff. (1 vs 3)**	↑**	t(8) = 5.47, p < 0.01
				subgroup diff. (2 vs 3)**	↑**	t(8) = 4.54, p < 0.01
				subgroup diff. (1 vs 2)	→	t(10) = 1.98, p = 0.08
	Complex Attention:					
	meta-regression	Mansor et al. 2020 [3]	☐ COG ☐ PHYS ☒ DUAL	exergame		coefficient (SE) = 1.57 (1.02), p = 0.16
				brain game**	↓**	coefficient (SE) = -1.67 (0.44), p < 0.01
	subgroup analysis	Law et al. 2020 [22]	☐ COG ☒ PHYS ☐ DUAL	pooled estimate		SMD^+ = 0.04, p = 0.52, 95 % CI [-0.07 to 0.15], k = 15
				aerobic training only		SMD^+ = 0.12, 95 % CI [-0.06 to 0.30], k = 6
				between-group difference		NR
	subgroup analysis	Bahar-Fuchs et al. 2019 [5]	☒ COG ☐ PHYS ☐ DUAL	pooled estimate*		SMD^+ = 0.56, p = 0.02, 95 % CI [0.07 to 0.95], k = 12
				traditional training		SMD^+ = 0.56, p = 0.13, 95 % CI [- 0.17 to 1.30], k = 8
				augmented training*		SMD^+ = 0.51, p = 0.02, 95 % CI [0.07 to 0.95], k = 4
				between-group difference		Chi^2 = 0.01, p = 0.91, I^2 = 0 %
	Executive Function:					
	subgroup analysis	Biazus-Sehn et al. 2020 [19]	☐ COG ☒ PHYS ☒ DUAL	pooled estimate*		SMD^+ = 0.21, p = 0.026, 95 % CI [0.03 to 0.40], k = 19
				aerobic training only		SMD^+ = 0.13, p = 0.407, 95 % CI [-0.18 to 0.44]
				resistance training only		SMD^+ = 0.14, p = 0.499, 95 % CI [-0.26 to 0.53]
				multicomp. training only		SMD^+ = 0.04, p = 0.928, 95 % CI [-0.81 to 0.90]
				comb. cognitive-motor*	↑*	SMD^+ = 0.50, p = 0.015, 95 % CI [0.93 to 0.91]
				between-group difference		NR
	subgroup analysis	Bahar-Fuchs et al. 2019 [5]	☒ COG ☐ PHYS ☐ DUAL	pooled estimate*		SMD^+ = 0.75, p = 0, 95 % CI [0.28 to 1.22], k = 12
				traditional training*		SMD^+ = 0.64, p = 0.01, 95 % CI [0.14 to 1.14], k = 9
				augmented training		SMD^+ = 1.32, p = 0.10, 95 % CI [- 0.26 to 2.91], k = 3
				between-group difference		Chi^2 = 0.65, p = 0.42, I^2 = 0 %
				pooled estimate		SMD^+ = 0.03, p = 0.74, 95 % CI [-0.14 to 0.20], k = 8
				aerobic training only		SMD^+ = 0.01, 95 % CI [-0.16 to 0.19], k = 6
				between-group difference	→	NR
	Cognitive Flexibility:					
	subgroup analysis	Law et al. 2020 [22]	☐ COG ☒ PHYS ☐ DUAL	pooled estimate*		MD^- = -8.80, p = 0.007, 95 % CI [-15.2 to -2.4], k = 13
				dance only		MD^- = -6.19, p = 0.38, 95 % CI [-20.1 to 7.7], k = 3
				tai chi only		MD^- = -9.06, p = 0.11, 95 % CI [-20.0 to 1.9], k = 7
	subgroup analysis	Wu et al. 2019 [43]	☐ COG ☐ PHYS ☒ DUAL	between-group difference		p = 0.82, I^2 = 0 %
	Inhibition:					
	meta-regression	Mansor et al. 2020 [3]	☐ COG ☐ PHYS ☒ DUAL	exergame	→	coefficient (SE) = 0.28 (0.28), p = 0.34
				brain game	→	coefficient (SE) = 0.13 (0.31), p = 0.71
	Shifting:					
	meta-regression	Mansor et al. 2020 [3]	☐ COG ☐ PHYS ☒ DUAL	exergame	→	coefficient (SE) = 0.49 (0.41), p = 0.25
				brain game	→	coefficient (SE) = 0.13 (0.31), p = 0.71
	Updating:					
	meta-regression	Mansor et al. 2020 [3]	☐ COG ☐ PHYS ☒ DUAL	exergame	→	coefficient (SE) = -0.32 (0.41), p = 0.44
				brain game	→	coefficient (SE) = 0.18 (0.39), p = 0.65
	Working Memory:					
	subgroup analysis	Wu et al. 2019 [43]	☐ COG ☐ PHYS ☒ DUAL	pooled estimate*		MD^+ = 0.32, p = 0.05, 95 % CI [0.01 to 0.64], k = 10
				dance only		MD^+ = 0.41, p = 0.18, 95 % CI [-0.19 to 1.00], k = 2
				tai chi only		MD^+ = 0.46, p = 0.06, 95 % CI [-0.03 to 0.94], k = 6
				between-group difference	→	p = 0.52, I^2 = 0 %
	Learning & Memory:					
	subgroup analysis	Biazus-Sehn et al. 2020 [19]	☐ COG ☒ PHYS ☒ DUAL	pooled estimate*		SMD^+ = 0.18, p = 0.047, 95 % CI [0.00 to 0.36], k = 17
				aerobic training only		SMD^+ = 0.07, p = 0.596, 95 % CI [-0.19 to 0.34]
				resistance training only		SMD^+ = -0.04, p = 0.856, 95 % CI [-0.44 to 0.37]
				multicomp. training only		SMD^+ = 0.01, p = 0.973, 95 % CI [-0.38 to 0.39]
				comb. cognitive-motor**	↑**	SMD^+ = 0.59, p < 0.001, 95 % CI [0.27 to 0.92]

Type (of training; continued)	subgroup analysis	Bahar-Fuchs et al. 2019 [5]	☒ COG ☐ PHYS ☐ DUAL	between-group difference<br>immediate memory:<br>pooled estimate*<br>traditional training*<br>augmented training*<br>between-group difference →<br>delayed memory:<br>between-group difference<br>traditional training*<br>augmented training<br>between-group difference →	NR<br><br>SMD^+ = 0.74, p < 0.001, 95 % CI [0.04 to 1.02], k = 18<br>SMD^+ = 0.82, p = 0, 95 % CI [0.33 to 1.30], k = 13<br>SMD^+ = 0.53, p = 0.03, 95 % CI [0.04 to 1.02], k = 5<br>Chi^2 = 0.65, p = 0.42, I^2 = 0 %<br><br>SMD^+ = 0.81, p = 0, 95 % CI [0.29 to 1.32], k = 11<br>SMD^+ = 0.86, p = 0.02, 95 % CI [0.15 to 1.58], k = 7<br>SMD^+ = 0.68, p = 0.09, 95 % CI [- 0.11 to 1.46], k = 4<br>Chi^2 = 0.12, p = 0.73, I^2 = 0 %
	Language Fluency:<br>subgroup analysis	Bahar-Fuchs et al. 2019 [5]	☒ COG ☐ PHYS ☐ DUAL	pooled estimate*<br>traditional training*<br>augmented training<br>between-group difference →	SMD^+ = 0.52, p = 0.0005, 95 % CI [0.23 to 0.81], k = 9<br>SMD^+ = 0.64, p = 0, 95 % CI [0.26 to 1.01], k = 6<br>SMD^+ = 0.33, p = 0.05, 95 % CI [0 to 0.65], k = 3<br>Chi^2 = 1.5, p = 0.22, I^2 = 33.37 %
	subgroup analysis	Wu et al. 2019 [43]	☐ COG ☐ PHYS ☒ DUAL	pooled estimate*<br>dance only**<br>tai chi only*<br>between-group difference →	MD^+ = 0.27, p = 0.003, 95 % CI [0.09 to 0.45], k = 9<br>MD^+ = 0.61, p < 0.001, 95 % CI [0.09 to 0.45], k = 1<br>MD^+ = 0.18, p = 0.05, 95 % CI [0.00 to 0.37], k = 6<br>p = 0.11, I^2 = 55.5 %
	Memory:<br>subgroup analysis	Wu et al. 2019 [43]	☐ COG ☐ PHYS ☒ DUAL	pooled estimate**<br>dance only*<br>tai chi only*<br>between-group difference →	MD^+ = 0.24, p = 0.001, 95 % CI [0.10 to 0.39], k = 15<br>MD^+ = 0.35, p = 0.003, 95 % CI [0.12 to 0.58], k = 3<br>MD^+ = 0.20, p = 0.005, 95 % CI [0.06 to 0.35], k = 7<br>p = 0.49, I^2 = 0 %
	Processing Speed:<br>subgroup analysis	Wu et al. 2019 [43]	☐ COG ☐ PHYS ☒ DUAL	pooled estimate<br>dance only<br>tai chi only<br>between-group difference →	MD^- = -1.44, p = 0.39, 95 % CI [-4.7 to 1.8], k = 9<br>MD^- = -0.69, p = 0.22, 95 % CI [-1.78 to 0.41], k = 3<br>MD^- = -1.29, p = 0.74, 95 % CI [-9.0 to 6.4], k = 4<br>p = 0.94, I^2 = 0 %
	Healthy Older Adults:				
	Global Cognition:<br>network meta-analysis	Gavelin et al. 2021 [39]	☒ COG ☒ PHYS ☒ DUAL	pairwise comparisons<br>pooled estimate*<br>simultaneous training*<br>sequential training*<br>cognitive training*<br>physical training*<br>exergaming*	see table 4 of publication<br>g^+ = 0.20, p < 0.001, 95 % CI [0.12 to 0.29], k = 28<br>g^+ = 0.45, p < 0.05, 95 % CI [0.23 to 0.53], k = 2<br>g^+ = 0.38, p < 0.05, 95 % CI [0.22 to 0.56], k = 2<br>g^+ = 0.36, p < 0.05, 95 % CI [0.16 to 0.41], k = 1<br>g^+ = 0.24, p < 0.05, 95 % CI [0.07 to 0.38], k = 1<br>g^+ = 0.21, p < 0.05, 95 % CI [0.04 to 0.35], k = 5
	moderator analysis	Sanders et al. 2019 [26]	☐ COG ☒ PHYS ☐ DUAL	moderator effect →<br>pooled estimate*<br>aerobic only*<br>resistance only**<br>multicomponent only	F(3, 128) = 0.081, p > 0.05<br>g^+ = 0.26, p < 0.01, 95 % CI [0.15 to 0.37], k = 23<br>g^+ = 0.22, p < 0.05, 95 % CI [0.03 to 0.41]<br>g^+ = 0.27, p < 0.01, 95 % CI [0.09 to 0.46]<br>g^+ = 0.30, p > 0.05, 95 % CI [-0.01 to 0.60]
	moderator analysis	Northey et al. 2018 [30]	☐ COG ☒ PHYS ☐ DUAL	moderator effect**<br>pooled estimate**<br>aerobic only**<br>resistance only**<br>multicomponent only** ↑**	Q_5 = 39.53, p < 0.01<br>SMD^+ = 0.29, p < 0.01, 95 % CI [0.17 to 0.41], k = 36<br>SMD^+ = 0.24, p < 0.01, 95 % CI [0.10 to 0.37], n = 153<br>SMD^+ = 0.29, p < 0.01, 95 % CI [0.13 to 0.44], n = 80<br>SMD^+ = 0.52, p < 0.01, 95 % CI [0.32 to 0.71], n = 80
	Executive Function:<br>moderator analysis	Chen et al. 2020 [20]	☐ COG ☒ PHYS ☒ DUAL	Type of training* ↑**<br>aerobic training*<br>resistance training*<br>multicomp. training*<br>Tai Chi / Yoga*<br>cognitive-motor training*	Q(4) = 226.18, p < 0.05<br>g^+ = 0.14, p < 0.05, 95 % CI [0.06 to 0.33], k = 45<br>g^+ = 0.22, p < 0.05, 95 % CI [0.10 to 0.33], k = 20<br>g^+ = 0.10, p < 0.05, 95 % CI [0.00 to 0.19], k = 18<br>g^+ = 0.38, p < 0.05, 95 % CI [0.27 to 0.49], k = 14<br>g^+ = 0.44, p < 0.05, 95 % CI [0.29 to 0.66], k = 10
	☐ Brain / Neurochemicals / HRV				
	☒ Physical Outcomes, Mobility, and Activities of Daily Living				
	Older Adults with mNCD/Dementia:				
	Global Physical Outc.:<br>network meta-analysis	Gavelin et al. 2021 [39]	☒ COG ☒ PHYS ☒ DUAL	pairwise comparisons<br>pooled estimate*<br>sequential training*<br>simultaneous training<br>physical training<br>exergaming<br>cognitive training	see table 4 of publication<br>g^+ = 0.32, p = 0.008, 95 % CI [0.12 to 0.52], k = 8<br>g^+ = 0.66, p < 0.05, 95 % CI [0.13 to 1.19], k = 2<br>g^+ = 0.36, p > 0.05, 95 % CI [-0.02 to 0.75], k = 2<br>g^+ = 0.26, p > 0.05, 95 % CI [-0.19 to 0.70], k = 1<br>g^+ = 0.22, p > 0.05, 95 % CI [-0.11 to 0.54], k = 1<br>g^+ = - 0.09, p > 0.05, 95 % CI [-0.57 to 0.40], k = 5
	Healthy Older Adults:				

Type (of training; continued)	Global Physical Outc.: network meta-analysis	Gavelin et al. 2021 [39]	☒ COG ☒ PHYS ☒ DUAL	pairwise comparisons	see table 4 of publication
				pooled estimate*	g^+ = 0.23, p = 0.005, 95 % CI [0.08 to 0.38], k = 24
				simultaneous training*	g^+ = 0.50, p < 0.05, 95 % CI [0.24 to 0.77], k = 2
				sequential training*	g^+ = 0.43, p < 0.05, 95 % CI [0.20 to 0.65], k = 2
				physical training*	g^+ = 0.38, p < 0.05, 95 % CI [0.15 to 0.61], k = 1
				exergaming*	g^+ = 0.23, p < 0.05, 95 % CI [0.03 to 0.43], k = 1
				cognitive training	g^+ = 0.18, p > 0.05, 95 % CI [-0.11 to 0.47], k = 5
	**☒ Psychosocial Outcomes**				
	Older Adults with mNCD/Dementia:				
	Depression: direct comparison	García-Casal et al. 2017 [11]	☒ COG ☒ PHYS ☐ DUAL	computer based vs. not →	SMD^+ = -0.02, p = 0.95, 95 % CI [-0.54 to 0.50], k = 3
	subgroup analysis	Chan et al. 2020 [2]	☒ COG ☐ PHYS ☐ DUAL	pooled estimate*	SMD^- = - 0.54, p < 0.001, 95 % CI [-0.77 to –0.31], k = 36
				computerized training*	SMD^- = - 0.77, p < 0.001, 95 % CI [-1.09 to –0.44], k = 7
				cognitive rehabilitation*	SMD^- = - 0.32, p < 0.001, 95 % CI [-0.57 to –0.07], k = 16
				cognitive stimulation*	SMD^- = - 0.61, p < 0.001, 95 % CI [-1.08 to –0.15], k = 14
				between-group difference	NR
	Participants' Mood: subgroup analysis	Bahar-Fuchs et al. 2019 [5]	☒ COG ☐ PHYS ☐ DUAL	pooled estimate	SMD^+ = 0.72, p = 0.08, 95 % CI [- 0.10 to 1.54], k = 8
				traditional training	SMD^+ = 0.9, p = 0.14, 95 % CI [0.3 to 2.1], k = 5
				augmented training	SMD^+ = 0.46, p = 0.52, 95 % CI [- 0.94 to 1.86], k = 3
				between-group difference	Chi^2 = 0.22, p = 0.64, I^2 = 0 %
Time (session duration)	**☒ Cognition**				
	Older Adults with mNCD/Dementia:				
	Global Cognition: moderator analysis	Sanders et al. 2019 [26]	☐ COG ☒ PHYS ☐ DUAL	moderator effect** ↓**	F(2, 51) = 5.756, p < 0.01
				pooled estimate*	g^+ = 0.22, p < 0.05, 95 % CI [0.05 to 0.39], k = 13
				≤ 30 min**	g^+ = 0.43, p < 0.01, 95 % CI [0.24 to 0.62]
				post-hoc test$_{reference = \geq 45min}$** ↓**	ß = 0.38, 95 % CI [0.15 to 0.60], p < 0.01
				31 – 45 min	g^+ = 0.28, p > 0.05, 95 % CI [-0.26 to 0.82]
				post-hoc test$_{reference = \geq 45min}$ →	ß = 0.23, 95 % CI [-0.23 to 0.78], p > 0.05
				≥ 45 min	g^+ = -0.07, p > 0.05, 95 % CI [-0.07 to 0.17]
	Mixed Healthy Older Adults and Older Adults with mNCD or Dementia:				
	Global Cognition: bivariate correlation	Gomes-Osman et al. 2018 [46]	☐ COG ☒ PHYS ☐ DUAL	session time (min) →	r = 0.20, p = 0.05 (n.s.)
	Healthy Older Adults:				
	Complex Attention: meta-regression	Mansor et al. 2020 [3]	☐ COG ☐ PHYS ☒ DUAL	sessions duration →	coefficient (SE) = -0.01 (0.02), p = 0.48
	Processing Speed: meta-regression	Mansor et al. 2020 [3]	☐ COG ☐ PHYS ☒ DUAL	sessions duration →	coefficient (SE) = -0.01 (0.01), p = 0.19
	Executive Function: moderator analysis	Chen et al. 2020 [20]	☐ COG ☒ PHYS ☒ DUAL	Session time →	Q(2) = 0.21, p > 0.05
				short (≤ 45 min)*	g^+ = 0.26, p < 0.05, 95 % CI [0.18 to 0.33], k = 50
				moderate (45 – 60 min)*	g^+ = 0.26, p < 0.05, 95 % CI [0.18 to 0.33], k = 39
				long (60+ min)*	g^+ = 0.30, p < 0.05, 95 % CI [0.15 to 0.44], k = 10
	Inhibition: meta-regression	Mansor et al. 2020 [3]	☐ COG ☐ PHYS ☒ DUAL	sessions duration →	coefficient (SE) = -0.00 (0.01), p = 0.60
	Reasoning: meta-regression	Mansor et al. 2020 [3]	☐ COG ☐ PHYS ☒ DUAL	sessions duration →	coefficient (SE) = -0.02 (0.02), p = 0.34
	Shifting: meta-regression	Mansor et al. 2020 [3]	☐ COG ☐ PHYS ☒ DUAL	sessions duration →	coefficient (SE) = -0.01 (0.01), p = 0.13
	Updating: meta-regression	Mansor et al. 2020 [3]	☐ COG ☐ PHYS ☒ DUAL	sessions duration →	coefficient (SE) = 0.01 (0.0.01), p = 0.25
	Global Cognition: moderator analysis	Sanders et al. 2019 [26]	☐ COG ☒ PHYS ☐ DUAL	moderator effect →	F(3, 129) = 0.905, p > 0.05
				pooled estimate**	g^+ = 0.26, p < 0.01, 95 % CI [0.15 to 0.37], k = 23
				≤ 30 min	g^+ = 0.22, p > 0.05, 95 % CI [-0.03 to 0.47]
				31 – 45 min	g^+ = 0.11, p > 0.05, 95 % CI [-0.16 to 0.38]
				≥ 45 min**	g^+ = 0.31, p < 0.01, 95 % CI [0.16 to 0.45]
	subgroup analysis	Jia et al. 2019 [23]	☐ COG ☒ PHYS ☐ DUAL	pooled estimate**	SMD^+ = 1.12, p < 0.001, 95 % CI [0.66 to 1.59], k = 13
				up to 30 min/session* ↓*	SMD^+ = 1.92, 95 % CI [1.55 to 2.30], k = 6
				> 30 min/session*	SMD^+ = 0.34, 95 % CI [0.08 to 0.61], k = 7
				between-group difference*	NR, but no overlap of CI
	moderator analysis	Northey et al. 2018 [30]	☐ COG ☒ PHYS ☒ DUAL	moderator effect** ↑**	Q_3 = 27.83, p < 0.01
				pooled estimate**	SMD^+ = 0.29, p < 0.01, 95 % CI [0.17 to 0.41], k = 36
				≤ 45 min	SMD^+ = 0.09, p > 0.05, 95 % CI [-0.28 to 0.46], n = 36
				46 – 59 min*	SMD^+ = 0.31, p < 0.05, 95 % CI [0.16 to 0.46], n = 263

Time (session duration; continued)				≥ 60 min		SMD^+ = 0.33, p > 0.05, 95 % CI [-0.04 to 0.65], n = 24
	moderator analysis	Mewborn et al. 2017 [13]	☒ COG ☐ PHYS ☐ DUAL	moderator effect**	↓**	Q = 885.04, p = 0.001
				pooled estimate**		g^+ = 0.298, p < 0.001, 95 % CI [0.25 to 0.35], k = 279
				> 0.5 h**		g^+ = 0.302, p < 0.001, 95 % CI [0.25 to 0.36], k = 245
				< 0.5 h**		g^+ = 0.316, p < 0.001, 95 % CI [0.17 to 0.47], k = 29
	subgroup analysis	Lampit et al. 2014 [17]	☒ COG ☐ PHYS ☐ DUAL	pooled estimate**		g^+ = 0.22, p < 0.001, 95 % CI [0.15 to 0.29], k = 52
				< 30 min		g^+ = 0.15, p < 0.05, 95 % CI [0.00 to 0.31], k = 13
				31 – 60 min		g^+ = 0.24, p < 0.05, 95 % CI [0.14 to 0.34], k = 29
				> 60 min		g^+ = 0.23, p < 0.05, 95 % CI [0.06 to 0.40], k = 8
				between-group difference	→	p = 0.62
	Learning and Memory:					
	meta-regression	Mansor et al. 2020 [3]	☐ COG ☐ PHYS ☒ DUAL	sessions duration**	↓**	coefficient (SE) = -0.03 (0.01), p = 0.03
	☒ Brain / Neurochemicals / HRV					
	Healthy Older Adults:					
	Heart Rate Variability:					
	ST-SDNN					
	meta-regression	Raffin et al. 2019 [25]	☐ COG ☒ PHYS ☐ DUAL	training duration	→	coefficient (SE) = 0.003 (0.008), p = 0.760
	RMSSD					
	meta-regression	Raffin et al. 2019 [25]	☐ COG ☒ PHYS ☐ DUAL	training duration	→	coefficient (SE) = 0.003 (0.012), p = 0.829
	HF-HRV					
	meta-regression	Raffin et al. 2019 [25]	☐ COG ☒ PHYS ☐ DUAL	training duration	→	coefficient (SE) = 0.008 (0.006), p = 0.152
	☐ Physical Outcomes, Mobility, and Activities of Daily Living					
	☐ Psychosocial Outcomes					
Duration (of intervention)	☒ Cognition					
	Older Adults with mNCD/Dementia:					
	Global Cognition:					
	subgroup-analysis	Bahar-Fuchs et al. 2019 [5]	☒ COG ☐ PHYS ☐ DUAL	pooled estimate		SMD^+ = 0.42, p < 0.0001, 95 % CI [0.23 to 0.61], k = 27
				≤ 3 months*		SMD^+ = 0.38, p = 0, 95 % CI [0.18 to 0.58], k = 21
				> 3 months*		SMD^+ = 0.54, p = 0.02, 95 % CI [0.07 to 1.01], k = 6
				between-group diff.		Chi^2 = 0.38, p = 0.54, I^2 = 0 %
	subgroup-analysis	Wu et al. 2019 [43]	☐ COG ☒ PHYS ☒ DUAL	pooled estimate**		SMD^+ = 0.92, p < 0.001 95 % CI [0.33 to 0.59], k = 13
				0 – 12 weeks*		SMD^+ = 1.74, p = 0.03, 95 % CI [0.18 to 3.29], k = 4
				13 – 14 weeks*		SMD^+ = 0.46, p = 0.04, 95 % CI [0.02 to 0.90], k = 6
				> 24 weeks*		SMD^+ = 0.58, p = 0.05, 95 % CI [0.01 to 1.15], k = 3
				between-group difference	→	p = 0.30; I^2 = 17 %
	moderator analysis	Sanders et al. 2019 [26]	☐ COG ☒ PHYS ☐ DUAL	moderator effect	→	F(2, 51) = 1.259, p > 0.05
				pooled estimate*		g^+ = 0.22, p < 0.05, 95 % CI [0.05 to 0.39], k = 13
				4 – 12 weeks		g^+ = 0.29, p > 0.05, 95 % CI [-0.01 to 0.58]
				13 – 24 weeks*		g^+ = 0.34, p < 0.05, 95 % CI [0.05 to 0.53]
				≥ 24 weeks		g^+ = 0.10, p > 0.05, 95 % CI [-0.16 to 0.36]
	Learning & Memory:					
	subgroup-analysis	Bahar-Fuchs et al. 2019 [5]	☒ COG ☐ PHYS ☐ DUAL	pooled estimate		SMD^+ = 0.74, p < 0.0001, 95 % CI [0.37 to 1.12], k = 18
				≤ 3 months		SMD^+ = 0.03, p = 0.84, 95 % CI [- 0.23 to 0.28], k = 9
				> 3 months		SMD^+ = 0.66, p = 0.07, 95 % CI [- 0.05 to 1.38], k = 3
				between-group diff.	→	Chi^2 = 2.71, p = 0.10, I^2 = 63.13 %
	Language:					
	subgroup-analysis	Bahar-Fuchs et al. 2019 [5]	☒ COG ☐ PHYS ☐ DUAL	pooled estimate		SMD^+ = 0.22, p = 0.13, 95 % CI [- 0.07 to 0.50], k = 12
				≤ 3 months*		SMD^+ = 0.75, p = 0, 95 % CI [0.29 to 1.21], k = 15
				> 3 months*		SMD^+ = 0.76, p = 0, 95 % CI [0.26 to 1.26], k = 3
				between-group diff.	→	Chi^2 = 0, p = 0.97, I^2 = 0 %
	Mixed Healthy Older Adults and Older Adults with mNCD or Dementia:					
	Global Cognition:					
	moderator analysis	Gavelin et al. 2021 [39]	☐ COG ☐ PHYS ☒ DUAL	moderator effect	→	F(1, 18) = 0.32, p = 0.58
				pooled estimate*		g^+ = 0.22, p < 0.05, 95 % CI [0.14 to 0.30], k = 41
				≤ 12 weeks**		g^+ = 0.24, p < 0.001, 95 % CI [0.13 to 0.36], k = 25
				> 12 weeks**		g^+ = 0.19, p = 0.003, 95 % CI [0.13 to 0.30], k = 16
	bivariate correlation	Gomes-Osman et al. 2018 [46]	☐ COG ☒ PHYS ☐ DUAL	duration (weeks)	→	r = 0.15, p = 0.12
	Healthy Older Adults:					
	Global Cognition:					
	meta-regression	Bonnechère et al. 2020 [1]	☒ COG ☐ PHYS ☐ DUAL	meta-regression	→	ß = -0.007, SE = 0.006, p = 0.24
	meta-regression	Biazus-Sehn et al. 2020 [19]	☐ COG ☒ PHYS ☒ DUAL	length of trial	→	ß = -0.0042, R^2 = 0, p = 0.52

Duration (of intervention; continued)	moderator analysis	Sanders et al. 2019 [26]	☐ COG ☒ PHYS ☐ DUAL	moderator effect<br>pooled estimate**<br>4 – 12 weeks**<br>13 – 24 weeks<br>≥ 24 weeks*	→	$F(3, 128) = 1.487, p > 0.05$<br>$g^+ = 0.26, p < 0.01$, 95 % CI [0.15 to 0.37], k = 23<br>$g^+ = 0.36, p < 0.01$, 95 % CI [0.17 to 0.54]<br>$g^+ = 0.14, p > 0.05$, 95 % CI [-0.04 to 0.32]<br>$g^+ = 0.29, p < 0.05$, 95 % CI [0.06 to 0.52]
	moderator analysis	Northey et al. 2018 [30]	☐ COG ☒ PHYS ☒ DUAL	moderator effect**<br>pooled estimate**<br>4 – 12 weeks<br>13 – 24 weeks<br>≥ 24 weeks	→	$Q_3 = 23.32, p < 0.01$<br>$SMD^+ = 0.29, p < 0.01$, 95 % CI [0.17 to 0.41], k = 36<br>$SMD^+ = 0.31, p < 0.05$, 95 % CI [0.09 to 0.54], n = 78<br>$SMD^+ = 0.28, p < 0.05$, 95 % CI [0.10 to 0.47], n = 170<br>$SMD^+ = 0.27, p < 0.05$, 95 % CI [0.03 to 0.52], n = 86
	subgroup analysis	Stanmore et al. 2017 [44]	☐ COG ☐ PHYS ☒ DUAL	pooled estimate**<br>< 12 weeks*<br>> 12 weeks*<br>between-group difference	→	$g^+ = 0.436, p = 0.01$, 95 % CI [0.18 to 0.69], k = 17<br>$g^+ = 0.255, p = 0.030$, 95 % CI [0.02 to 0.49]<br>$g^+ = 0.759, p = 0.005$, 95 % CI [0.23 to 1.29]<br>$p = 0.086$
	moderator analysis	Toril et al. 2014 [16]	☒ COG ☐ PHYS ☐ DUAL	moderator effect*<br>pooled estimate**<br>short (1 – 6 weeks)**<br>long (7 – 12 weeks)**	↓*	$Q(1) = 3.73, p = 0.05$<br>$d^+ = 0.37, p < 0.01$, 95 % CI [0.26 to 0.48<br>$d^+ = 0.49, p < 0.01$, 95 % CI [0.32 to 0.67]<br>$d^+ = 0.26, p < 0.01$, 95 % CI [0.09 to 0.43]
	Executive Function:<br>moderator analysis	Chen et al. 2020 [20]	☐ COG ☒ PHYS ☒ DUAL	Length of Intervention*<br>short (1 – 3 months)*<br>medium (4 – 6 months)*<br>long (> 6 months)*	↓*	$Q(2) = 16.64, p < 0.05$<br>$g^+ = 0.32, p < 0.05$, 95 % CI [0.23 to 0.41], k = 29<br>$g^+ = 0.26, p < 0.05$, 95 % CI [0.18 to 0.34], k = 49<br>$g^+ = 0.09, p < 0.05$, 95 % CI [0.01 to 0.17], k = 29
	meta-regression	Biazus-Sehn et al. 2020 [19]	☐ COG ☒ PHYS ☒ DUAL	length of trial*	↓*	$ß = -0.0035, R^2 = 0.32, p < 0.047$
	subgroup analysis	Jia et al. 2019 [23]	☐ COG ☒ PHYS ☐ DUAL	pooled estimate**<br>up to 16 weeks*<br>> 16 weeks*	↑	$SMD^+ = 1.12, p < 0.001$, 95 % CI [0.66 to 1.59], k = 13<br>$SMD^+ = 0.91$, 95 % CI [0.40 to 1.43], k = 9<br>$SMD^+ = 1.12$, 95 % CI [0.66 to 1.59], k = 4
	☐ **Brain / Neurochemicals / HRV**					
	☒ **Physical Outcomes, Mobility, and Activities of Daily Living**					
	Mixed Healthy Older Adults and Older Adults with mNCD or Dementia:					
	Global Cognition:<br>moderator analysis	Gavelin et al. 2021 [39]	☐ COG ☐ PHYS ☒ DUAL	moderator effect<br>pooled estimate*<br>≤ 12 weeks**<br>> 12 weeks	↓*	$F(2, 18) = 4.52, p = 0.048$<br>$g^+ = 0.25, p < 0.05$, 95 % CI [0.13 to 0.37], k = 32<br>$g^+ = 0.33, p < 0.001$, 95 % CI [0.17 to 0.48], k = 21<br>$g^+ = 0.09, p = 0.31$, 95 % CI [-0.10 to 0.28], k = 11
	☒ **Psychosocial Outcomes**					
	Older Adults with mNCD/Dementia:					
	Neuropsych. Sympt.:<br>subgroup analysis	Bahar-Fuchs et al. 2019 [5]	☒ COG ☐ PHYS ☐ DUAL	pooled estimate<br>≤ 3 months<br>> months<br>between-group diff.	→	$SMD^+ = 0.44, p = 0.27$, 95 % CI [- 0.34 to 1.22], k = 6<br>$SMD^+ = 0.66, p = 0.59$, 95 % CI [- 1.70 to 3.02], k = 3<br>$SMD^+ = 0.12, p = 0.57$, 95 % CI [- 0.29 to 0.53], k = 3<br>$Chi^2 = 0.19, p = 0.66, I^2 = 0$ %
Volume / Dose	☒ **Cognition**					
	Older Adults with mNCD/Dementia:					
	Global Cognition:<br>subgroup analysis	Law et al. 2020 [22]	☐ COG ☒ PHYS ☐ DUAL	pooled estimate*<br>tot. $time_{training} \leq 24h$*<br>tot. $time_{training} > 24h$*<br>between-group difference		$SMD^+ = 0.44$, 95 % CI [0.27 to 0.61], k = 26<br>$SMD^+ = 0.23$, 95 % CI [0.07 to 0.40], k = 13<br>$SMD^+ = 0.66$, 95 % CI [0.32 to 0.99], k = 12<br>NR
	meta-regression	Biazus-Sehn et al. 2020 [19]	☐ COG ☒ PHYS ☒ DUAL	volume (min/week)*	↓*	$ß = -0.0048, R^2 = 0.33, p = 0.044$
	subgroup-analysis	Wu et al. 2019 [43]	☐ COG ☒ PHYS ☒ DUAL	pooled estimate**<br>low (< 60min/week)<br>mod. (60–120 min/week)*<br>high (> 120min/week)<br>between-group difference	→	$SMD^+ = 0.92, p < 0.001$ 95 % CI [0.33 to 0.59], k = 13<br>$SMD^+ = 0.41, p = 0.16$, 95 % CI [-0.16 to 0.97], k = 2<br>$SMD^+ = 1.15, p = 0.006$, 95 % CI [0.34 to 1.97], k = 8<br>$SMD^+ = 0.15, p = 0.51$, 95 % CI [-0.29 to 0.59], k = 3<br>$p = 0.11; I^2 = 55.5$ %
	subgroup analysis	Jia et al. 2019 [23]	☐ COG ☒ PHYS ☐ DUAL	pooled estimate**<br>up to 2h/week*<br>more than 2h/week*<br>between-group difference		$SMD^+ = 1.12, p < 0.001$, 95 % CI [0.66 to 1.59], k = 13<br>$SMD^+ = 1.74$, 95 % CI [1.32 to 2.15], k = 5<br>$SMD^+ = 0.76$, 95 % CI [0.27 to 1.24], k = 8<br>NR
	subgroup analysis	Zhang et al. 2019 [8]	☒ COG ☐ PHYS ☐ DUAL	pooled estimate<br>≤ 10 hours<br>> 10 hours<br>between-group difference		$g^+ = 0.23, p = 0.09$, 95 % CI [-0.04 to 0.50], k = 9<br>$g^+ = 0.30, p = 0.06$, 95 % CI [-0.01 to 0.61], k = 4<br>$g^+ = 0.20, p = 0.44$, 95 % CI [-0.31 to 0.71], k = 5<br>$Chi^2 = 0.11, p = 0.74, I^2 = 0$ %
	meta-regression	Shermann et al. 2017 [14]	☒ COG ☐ PHYS ☐ DUAL	volume (in hours)		Coefficient = -0.0005, Z-value = -0.07, p = 0.9412
	Attention:					

Volume / Dose (continued)	subgroup analysis	Law et al. 2020 [22]	☐ COG ☒ PHYS ☐ DUAL	pooled estimate<br>tot. time$_{training}$ ≤ 24h<br>tot. time$_{training}$ > 24h<br>between-group difference	SMD^+ = 0.04, p = 0.52, 95 % CI [-0.07 to 0.15], k = 15<br>SMD^+ = 0.05, 95 % CI [-0.09 to 0.19], k = 10<br>SMD^+ = -0.01, 95 % CI [-0.28 to 0.25], k = 5<br>NR
	Executive Function:<br>Working Memory:<br>subgroup analysis	Law et al. 2020 [22]	☐ COG ☒ PHYS ☐ DUAL	pooled estimate<br>tot. time$_{training}$ ≤ 24h<br>between-group difference	SMD^+ = 0.28, 95 % CI [0.04 to 0.52], k = 8<br>SMD^+ = 0.30, 95 % CI [0.02 to 0.58], k = 7<br>NR
	Learning and Memory:<br>subgroup analysis	Law et al. 2020 [22]	☐ COG ☒ PHYS ☐ DUAL	pooled estimate<br>tot. time$_{training}$ ≤ 24h<br>between-group difference	SMD^+ = 0.15, 95 % CI [-0.04 to 0.34], k = 11<br>SMD^+ = 0.16, 95 % CI [-0.04 to 0.36], k = 7<br>NR
	Language:<br>subgroup analysis	Law et al. 2020 [22]	☐ COG ☒ PHYS ☐ DUAL	pooled estimate<br>tot. time$_{training}$ ≤ 24h*<br>tot. time$_{training}$ > 24h<br>between-group difference	SMD^+ = 0.15, p = 0.11, 95 % CI [-0.03 to 0.34], k = 15<br>SMD^+ = 0.22, 95 % CI [0.02 to 0.42], k = 9<br>SMD^+ = 0.08, 95 % CI [-0.28 to 0.43], k = 5<br>NR
	Mixed Healthy Older Adults and Older Adults with mNCD or Dementia:				
	Global Cognition:<br>bivariate correlation	Gomes-Osman et al. 2018 [46]	☐ COG ☒ PHYS ☐ DUAL	tot. length of int.(hours)** ↑**	r = 0.24, p = 0.01
	Healthy Older Adults:				
	Global Cognition:<br>moderator analysis	Mewborn et al. 2017 [13]	☒ COG ☐ PHYS ☐ DUAL	moderator effect* ↑**<br>pooled estimate**<br>> 20h**<br>< 20h**<br>> 20 sessions<br>< 20 session	Q = 329.20, p = 0.035 AND Q = 885.04, p < 0.001<br>g^+ = 0.298, p < 0.001, 95 % CI [0.25 to 0.35], k = 279<br>g^+ = 0.341, p < 0.001, 95 % CI [0.23 to 0.45], k = 69<br>g^+ = 0.286, p < 0.001, 95 % CI [0.23 to 0.34], k = 208<br>g^+ = 0.329, p < 0.001, 95 % CI [0.24 to 0.42], k = 102<br>g^+ = 0.288, p < 0.001, 95 % CI [0.23 to 0.35], k = 172
	subgroup analysis	Lampit et al. 2014 [17]	☒ COG ☐ PHYS ☐ DUAL	pooled estimate**<br>≤ 20 h*<br>> 20 h*<br>between-group difference →	g^+ = 0.22, p < 0.001, 95 % CI [0.15 to 0.29], k = 52<br>g^+ = 0.23, p < 0.05, 95 % CI [0.15 to 0.32], k = 36<br>g^+ = 0.20, p < 0.05, 95 % CI [0.07 to 0.33], k = 15<br>p = 0.69
	Complex Attention:<br>meta-regression	Mansor et al. 2020 [3]	☐ COG ☐ PHYS ☒ DUAL	total duration (h) →	coefficient (SE) = -0.01 (0.02), p = 0.46
	Processing Speed:<br>meta-regression	Mansor et al. 2020 [3]	☐ COG ☐ PHYS ☒ DUAL	total duration (h) →	coefficient (SE) = -0.01 (0.18), p = 0.18
	Executive Function:<br>Inhibition:<br>meta-regression	Mansor et al. 2020 [3]	☐ COG ☐ PHYS ☒ DUAL	total duration (h) →	coefficient (SE) = -0.01 (0.01), p = 0.29
	Shifting:<br>meta-regression	Mansor et al. 2020 [3]	☐ COG ☐ PHYS ☒ DUAL	total duration (h)** ↓**	coefficient (SE) = -0.04 (0.01), p < 0.01
	Updating:<br>meta-regression	Mansor et al. 2020 [3]	☐ COG ☐ PHYS ☒ DUAL	total duration (h) →	coefficient (SE) = 0.00 (0.1), p = 0.92
	Reasoning:<br>meta-regression	Mansor et al. 2020 [3]	☐ COG ☐ PHYS ☒ DUAL	total duration (h) →	coefficient (SE) = -0.02 (0.02), p = 0.24
	Learning and Memory:<br>meta-regression	Mansor et al. 2020 [3]	☐ COG ☐ PHYS ☒ DUAL	total duration (h) →	coefficient (SE) = -0.03 (0.15), p = 0.07
	☒ Brain / Neurochemicals / HRV				
	Healthy Older Adults:				
	Heart Rate Variability:<br>ST-SDNN<br>meta-regression	Raffin et al. 2019 [25]	☐ COG ☒ PHYS ☐ DUAL	number of sessions →	coefficient (SE) = 0.002 (0.002), p = 0.286
	RMSSD<br>meta-regression	Raffin et al. 2019 [25]	☐ COG ☒ PHYS ☐ DUAL	number of sessions →	coefficient (SE) = 0.000 (0.002), p = 0.948
	HF-HRV<br>meta-regression	Raffin et al. 2019 [25]	☐ COG ☒ PHYS ☐ DUAL	number of sessions →	coefficient (SE) = 0.002 (0.001), p = 0.134
	☒ Physical Outcomes, Mobility, and Activities of Daily Living				
	Healthy Older Adults:				
	Fall Rate<br>meta-regression	Sherrington et al. 2017 [33]	☐ COG ☒ PHYS ☐ DUAL	hours per week of training<br>2+ hours/week<br>3+ hours/week* ↑*	 RR^- (95 % CI) = 0.98 (0.83 to 1.16), p = 0.83, ΔI^2 = - 6 %<br>RR^- (95 % CI) = 0.77 (0.65 to 0.91), p = 0.003, ΔI^2 = 61 %
	Funct. Exerc. Capacity:				

Volume / Dose (continued)	subgroup analysis	Howes et al. 2017 [45]	☐ COG ☐ PHYS ☒ DUAL	pooled estimate** ↑<br>< 120 min/week<br>> 120 min/week**<br>> 150 min/week*<br>subgroup difference →	SMD^+ = 0.29, p = 0.01, 95 % CI [0.04 to 0.55], k = 7<br>SMD^+ = 0.10, p = 0.58, 95 % CI [-0.24 to 0.44], k = 2<br>SMD^+ = 0.53, p = 0.006, 95 % CI [0.15 to 0.90], k = 5<br>SMD^+ = 0.85, p = 0.01, 95 % CI [0.19 to 1.51], k = 2<br>p = 0.17, I^2 = 45.8 %
	☒ Psychosocial Outcomes				
	Older Adults with mNCD/Dementia:				
	Behavioral Problems:<br>subgroup analysis	Law et al. 2020 [22]	☐ COG ☒ PHYS ☐ DUAL	pooled estimate**<br>tot. $time_{training}$ ≤ 24h*<br>between-group difference	SMD^+ = 0.35, p = 0.01, 95 % CI [0.07 to 0.64], k = 9<br>SMD^+ = 0.28, 95 % CI [0.10 to 0.46], k = 6<br>NR
Progression & Periodization	☐ Cognition				
	☐ Brain / Neurochemicals / HRV				
	☐ Physical Outcomes, Mobility, and Activities of Daily Living				
	☐ Psychosocial Outcomes				
Variability / Variation	☒ Cognition				
	Healthy Older Adults:				
	Global Cognition:<br>moderator analysis	Toril et al. 2014 [16]	☒ COG ☐ PHYS ☐ DUAL	moderator effect →<br>pooled estimate**<br>many (7 – 12) games**<br>few (1 – 6) games**	Q(1) = 0.37, p > 0.05<br>d^+ = 0.37, p < 0.01, 95 % CI [0.26 to 0.48<br>d^+ = 0.30, p < 0.01, 95 % CI [0.07 to 0.54]<br>d^+ = 0.39, p < 0.01, 95 % CI [0.26 to 0.51]
	☐ Brain / Neurochemicals / HRV				
	☐ Physical Outcomes, Mobility, and Activities of Daily Living				
	☐ Psychosocial Outcomes				
Specificity	☒ Cognition				
	Older Adults with mNCD/Dementia:				
	Global Cognition:<br>subgroup analysis	Bahar-Fuchs et al. 2019 [5]	☒ COG ☐ PHYS ☐ DUAL	pooled estimate*<br>single-domain training*<br>multi-domain training*<br>between-group difference →	SMD^+ = 0.42, p < 0.001, 95 % CI [0.23 to 0.61], k = 27<br>SMD^+ = 0.35, p = 0.01, 95 % CI [0.1 to 0.59], k = 10<br>SMD^+ = 0.44, p = 0, 95 % CI [0.18 to 0.71], k = 17<br>Chi^2 = 0.28, p = 0.6, I^2 = 0 %
	subgroup analysis	Zhang et al. 2019 [8]	☒ COG ☐ PHYS ☐ DUAL	pooled effect ↑*<br>single-domain training<br>multi-domain training*<br>between-group difference	g^+ = 0.30, p = 0.002, 95 % CI 0.11 to 0.50], k = 13<br>g^+ = 0.31, p = 0.23, 95 % CI [-0.19 to 0.81], k = 3<br>g^+ = 0.30, p = 0.008, 95 % CI [0.08 to 0.53], k = 10<br>Chi^2 = 0.00, p = 0.99, I^2 = 0 %
	subgroup analysis	Shermann et al. 2017 [14]	☒ COG ☐ PHYS ☐ DUAL	pooled effect** →<br>attention-specific<br>processing speed-specific<br>memory-specific*<br>multi-domain training**<br>between-group difference**	g^+ = 0.420, p = 0.001, 95 % CI [0.182 to 0.659], k = 26<br>g^+ = 0.585, p = 0.212, 95 % CI [-0.334 to 1.504], k = 2<br>g^+ = 0.636, p = 0.120, 95 % CI [0.166 to 1.438], k = 2<br>g^+ = 0.671, p = 0.005, 95 % CI [0.207 to 1.136], k = 8<br>g^+ = 0.449, p = 0.005, 95 % CI [0.449 to 0.763], k = 14<br>Q(3) = 3.449, p = 0.001
	Complex Attention:<br>subgroup analysis	Bahar-Fuchs et al. 2019 [5]	☒ COG ☐ PHYS ☐ DUAL	pooled estimate*<br>single-domain training<br>multi-domain training<br>between-group difference	SMD^+ = 0.56, p = 0.02, 95 % CI [0.07 to 0.95], k = 12<br>SMD^+ = 0.68, p = 0.10, 95 % CI [-0.14 to 1.49], k = 4<br>SMD^+ = 0.5, p = 0.12, 95 % CI [-0.14 to 1.15], k = 8<br>Chi^2 = 0.11, p = 0.74, I^2 = 0 %

Specificity (continued)	Executive Function:				
	subgroup analysis	Bahar-Fuchs et al. 2019 [5]	☒ COG ☐ PHYS ☐ DUAL	pooled estimate*	SMD$^+$ = 0.75, p = 0, 95 % CI [0.28 to 1.22], k = 12
				single-domain training	SMD$^+$ = 0.08, p = 0.8, 95 % CI [-0.53 to 0.68], k = 3
				multi-domain training* ↑*	SMD$^+$ = 0.99, p = 0, 95 % CI [0.44 to 1.55], k = 9
				between-group difference*	Chi^2 = 4.78, p = 0.03, I^2 = 79.08 %
	Learning and Memory:				
	subgroup analysis	Bahar-Fuchs et al. 2019 [5]	☒ COG ☐ PHYS ☐ DUAL	<u>immediate memory:</u>	
				pooled estimate*	SMD$^+$ = 0.74, p < 0.001, 95 % CI [0.04 to 1.02], k = 18
				single-domain training**	SMD$^+$ = 0.58, p = 0, 95 % CI [0.22 to 0.94], k = 7
				multi-domain training**	SMD$^+$ = 0.8, p = 0, 95 % CI [0.24 to 1.35], k = 11
				between-group difference	Chi^2 = 0.41, p = 0.52, I^2 = 0 %
				<u>delayed memory:</u>	
				pooled estimate*	SMD$^+$ = 0.81, p = 0, 95 % CI [0.29 to 1.32], k = 11
				single-domain training	SMD$^+$ = 0.41, p = 0.12, 95 % CI [-0.11 to 0.94], k = 5
				multi-domain training*	SMD$^+$ = 1.15, p = 0.01, 95 % CI [0.32 to 1.97], k = 6
				between-group difference	Chi^2 = 2.15, p = 0.14, I^2 =53.48 %
	subgroup analysis	Shermann et al. 2017 [14]	☒ COG ☐ PHYS ☐ DUAL	pooled effect**	g$^+$ = 0.659, p = 0.000, 95 % CI [0.383 to 0.936], k = 20
				memory-specific training*	g$^+$ = 1.219, p = 0.007, 95 % CI [0.338 to 2.100], k = 6
				between group difference	NR
	Language Fluency:				
	subgroup analysis	Bahar-Fuchs et al. 2019 [5]	☒ COG ☐ PHYS ☐ DUAL	pooled estimate*	SMD$^+$ = 0.52, p = 0.0005, 95 % CI [0.23 to 0.81], k = 9
				single-domain training	SMD$^+$ = 0.14, p = 0.49, 95 % CI [- 0.25 to 0.52], k = 3
				multi-domain training*	SMD$^+$ = 0.70, p < 0.0001, 95 % CI [0.38 to 1.02], k = 6
				between-group difference* ↑*	Chi^2 = 3.81, p = 0.03, I^2 = 79.22 %
	Healthy Older Adults:				
	Global cognition:				
	moderator analysis	Mewborn et al. 2017 [13]	☒ COG ☐ PHYS ☐ DUAL	moderator effect*	Q = 322.32, p = 0.035
				pooled estimate**	g$^+$ = 0.298, p < 0.001, 95 % CI [0.25 to 0.35], k = 279
				working memoy train.**	g$^+$ = 0.479, p < 0.001, 95 % CI [0.33 to 0.63], k = 56
				memory training**	g$^+$ = 0.349, p < 0.001, 95 % CI [0.24 to 0.46], k = 51
				multi-domain training**	g$^+$ = 0.313, p < 0.001, 95 % CI [0.22 to 0.40], k = 106
				single-domain training**	g$^+$ = 0.288, p < 0.001, 95 % CI [0.23 to 0.35], k = 173
				processing speed train.*	g$^+$ = 0.140, p < 0.05, 95 % CI [0.00 to 0.28], k = 28
				attention training	g$^+$ = 0.169, p > 0.05, 95 % CI [-0.04 to 0.38], k = 7
				execut. funct. train.	g$^+$ = 0.125, p > 0.05, 95 % CI [-0.01 to 0.26], k = 24
				reasoning training	g$^+$ = 0.081, p > 0.05, 95 % CI [-0.12 to 0.29], k = 7
	moderator analysis	Toril et al. 2014 [16]	☒ COG ☐ PHYS ☐ DUAL	moderator effect	Q(3) = 3.449, p = 0.001
				pooled estimate**	d$^+$ = 0.37, p < 0.01, 95 % CI [0.26 to 0.48
				brain training**	d$^+$ = 0.34, p < 0.01, 95 % CI [0.17 to 0.50]
				video games**	d$^+$ = 0.40, p < 0.01, 95 % CI [0.25 to 0.55]
	subgroup analysis	Lampit et al. 2014 [17]	☒ COG ☐ PHYS ☐ DUAL	pooled estimate**	g$^+$ = 0.22, p < 0.001, 95 % CI [0.15 to 0.29], k = 52
				video games*	g$^+$ = 0.42, p < 0.05, 95 % CI [0.09 to 0.76], k = 4
				attention CCT*	g$^+$ = 0.34, p < 0.05, 95 % CI [0.08 to 0.60], k = 6
				speed of proc. CCT*	g$^+$ = 0.25, p < 0.05, 95 % CI [0.10 to 0.39], k = 9
				multidomain CCT*	g$^+$ = 0.18, p < 0.05, 95 % CI [0.08 to 0.29], k = 23
				working memory CCT	g$^+$ = 0.17, p > 0.05, 95 % CI [-0.03 to 0.38], k = 9
				between-group heterog.	p = 0.57
	Learning and Memory:				
	subgroup analysis	Bahar-Fuchs et al. 2019 [5]	☒ COG ☐ PHYS ☐ DUAL	<u>immediate memory:</u>	
				pooled estimate*	SMD$^+$ = 0.74, p < 0.001, 95 % CI [0.04 to 1.02], k = 18
				single-domain training**	SMD$^+$ = 0.58, p = 0, 95 % CI [0.22 to 0.94], k = 7
				multi-domain training**	SMD$^+$ = 0.8, p = 0, 95 % CI [0.24 to 1.35], k = 11
				between-group difference	Chi^2 = 0.41, p = 0.52, I^2 = 0 %
				<u>delayed memory:</u>	
				pooled estimate*	SMD$^+$ = 0.81, p = 0, 95 % CI [0.29 to 1.32], k = 11
				single-domain training	SMD$^+$ = 0.41, p = 0.12, 95 % CI [-0.11 to 0.94], k = 5
				multi-domain training*	SMD$^+$ = 1.15, p = 0.01, 95 % CI [0.32 to 1.97], k = 6
				between-group difference	Chi^2 = 2.15, p = 0.14, I^2 =53.48 %
	subgroup analysis	Shermann et al. 2017 [14]	☒ COG ☐ PHYS ☐ DUAL	pooled effect**	
				memory-specific training*	g$^+$ = 0.659, p = 0.000, 95 % CI [0.383 to 0.936], k = 20
				between group difference	g$^+$ = 1.219, p = 0.007, 95 % CI [0.338 to 2.100], k = 6
					NR
	☐ Brain / Neurochemicals / HRV				
	☐ Physical Outcomes, Mobility, and Activities of Daily Living				
	☐ Psychosocial Outcomes				

1 References

1. Bonnechère, B., C. Langley, and B.J. Sahakian, *The use of commercial computerised cognitive games in older adults: a meta-analysis.* Scientific Reports, 2020. **10**(1): p. 15276.
2. Chan, J.Y.C., et al., *Cognitive training interventions and depression in mild cognitive impairment and dementia: a systematic review and meta-analysis of randomized controlled trials.* Age Ageing, 2020. **49**(5): p. 738-747.
3. Mansor, N.S., C.M. Chow, and M. Halaki, *Cognitive effects of video games in older adults and their moderators: a systematic review with meta-analysis and meta-regression.* Aging Ment Health, 2020. **24**(6): p. 841-856.
4. Gates, N.J., et al., *Computerised cognitive training for 12 or more weeks for maintaining cognitive function in cognitively healthy people in late life.* Cochrane Database Syst Rev, 2020. **2**(2): p. Cd012277.
5. Bahar-Fuchs, A., et al., *Cognitive training for people with mild to moderate dementia.* Cochrane Database Syst Rev, 2019. **3**(3): p. Cd013069.
6. Gates, N.J., et al., *Computerised cognitive training for preventing dementia in people with mild cognitive impairment.* Cochrane Database Syst Rev, 2019. **3**(3): p. Cd012279.
7. Vaportzis, E., M.A. Niechcial, and A.J. Gow, *A systematic literature review and meta-analysis of real-world interventions for cognitive ageing in healthy older adults.* Ageing Res Rev, 2019. **50**: p. 110-130.
8. Zhang, H., et al., *Effect of computerised cognitive training on cognitive outcomes in mild cognitive impairment: a systematic review and meta-analysis.* BMJ Open, 2019. **9**(8): p. e027062.
9. Liang, J.H., et al., *Comparison of multiple interventions for older adults with Alzheimer disease or mild cognitive impairment: A PRISMA-compliant network meta-analysis.* Medicine (Baltimore), 2018. **97**(20): p. e10744.
10. Sala, G., K.S. Tatlidil, and F. Gobet, *Video game training does not enhance cognitive ability: A comprehensive meta-analytic investigation.* Psychological bulletin, 2018. **144**(2): p. 111.
11. García-Casal, J.A., et al., *Computer-based cognitive interventions for people living with dementia: a systematic literature review and meta-analysis.* Aging Ment Health, 2017. **21**(5): p. 454-467.
12. Hill, N.T., et al., *Computerized Cognitive Training in Older Adults With Mild Cognitive Impairment or Dementia: A Systematic Review and Meta-Analysis.* Am J Psychiatry, 2017. **174**(4): p. 329-340.
13. Mewborn, C.M., C.A. Lindbergh, and L. Stephen Miller, *Cognitive Interventions for Cognitively Healthy, Mildly Impaired, and Mixed Samples of Older Adults: A Systematic Review and Meta-Analysis of Randomized-Controlled Trials.* Neuropsychol Rev, 2017. **27**(4): p. 403-439.
14. Sherman, D.S., et al., *The Efficacy of Cognitive Intervention in Mild Cognitive Impairment (MNCD): a Meta-Analysis of Outcomes on Neuropsychological Measures.* Neuropsychol Rev, 2017. **27**(4): p. 440-484.
15. Melby-Lervåg, M., T.S. Redick, and C. Hulme, *Working Memory Training Does Not Improve Performance on Measures of Intelligence or Other Measures of "Far Transfer": Evidence*

From a Meta-Analytic Review. Perspectives on Psychological Science, 2016. **11**(4): p. 512-534.

16. Toril, P., J.M. Reales, and S. Ballesteros, *Video game training enhances cognition of older adults: a meta-analytic study.* Psychol Aging, 2014. **29**(3): p. 706-16.
17. Lampit, A., H. Hallock, and M. Valenzuela, *Computerized cognitive training in cognitively healthy older adults: a systematic review and meta-analysis of effect modifiers.* PLoS medicine, 2014. **11**(11): p. e1001756.
18. Karbach, J. and P. Verhaeghen, *Making working memory work: a meta-analysis of executive-control and working memory training in older adults.* Psychological science, 2014. **25**(11): p. 2027-2037.
19. Biazus-Sehn, L.F., et al., *Effects of physical exercise on cognitive function of older adults with mild cognitive impairment: A systematic review and meta-analysis.* Arch Gerontol Geriatr, 2020. **89**: p. 104048.
20. Chen, F.T., et al., *Effects of Exercise Training Interventions on Executive Function in Older Adults: A Systematic Review and Meta-Analysis.* Sports Med, 2020. **50**(8): p. 1451-1467.
21. Zhou, X.L., et al., *Effects of exercise interventions for specific cognitive domains in old adults with mild cognitive impairment: A meta-analysis and subgroup analysis of randomized controlled trials.* Medicine (Baltimore), 2020. **99**(31): p. e20105.
22. Law, C.-K., et al., *Physical exercise attenuates cognitive decline and reduces behavioural problems in people with mild cognitive impairment and dementia: a systematic review.* Journal of Physiotherapy, 2020. **66**(1): p. 9-18.
23. Jia, R.X., et al., *Effects of physical activity and exercise on the cognitive function of patients with Alzheimer disease: a meta-analysis.* BMC Geriatr, 2019. **19**(1): p. 181.
24. Marinus, N., et al., *The Impact of Different Types of Exercise Training on Peripheral Blood Brain-Derived Neurotrophic Factor Concentrations in Older Adults: A Meta-Analysis.* Sports Med, 2019. **49**(10): p. 1529-1546.
25. Raffin, J., et al., *Exercise Frequency Determines Heart Rate Variability Gains in Older People: A Meta-Analysis and Meta-Regression.* Sports Med, 2019. **49**(5): p. 719-729.
26. Sanders, L.M.J., et al., *Dose-response relationship between exercise and cognitive function in older adults with and without cognitive impairment: A systematic review and meta-analysis.* PLoS One, 2019. **14**(1): p. e0210036.
27. Wang, S., et al., *Efficacy of different types of exercises on global cognition in adults with mild cognitive impairment: a network meta-analysis.* Aging Clin Exp Res, 2019. **31**(10): p. 1391-1400.
28. Firth, J., et al., *Effect of aerobic exercise on hippocampal volume in humans: a systematic review and meta-analysis.* Neuroimage, 2018. **166**: p. 230-238.
29. Lam, F.M., et al., *Physical exercise improves strength, balance, mobility, and endurance in people with cognitive impairment and dementia: a systematic review.* J Physiother, 2018. **64**(1): p. 4-15.
30. Northey, J.M., et al., *Exercise interventions for cognitive function in adults older than 50: a systematic review with meta-analysis.* Br J Sports Med, 2018. **52**(3): p. 154-160.

31. Panza, G.A., et al., *Can Exercise Improve Cognitive Symptoms of Alzheimer's Disease?* Journal of the American Geriatrics Society, 2018. **66**(3): p. 487-495.

32. de Souto Barreto, P., et al., *Exercise Training for Preventing Dementia, Mild Cognitive Impairment, and Clinically Meaningful Cognitive Decline: A Systematic Review and Meta-analysis.* J Gerontol A Biol Sci Med Sci, 2018. **73**(11): p. 1504-1511.

33. Sherrington, C., et al., *Exercise to prevent falls in older adults: an updated systematic review and meta-analysis.* Br J Sports Med, 2017. **51**(24): p. 1750-1758.

34. Kelly, M.E., et al., *The impact of exercise on the cognitive functioning of healthy older adults: a systematic review and meta-analysis.* Ageing Res Rev, 2014. **16**: p. 12-31.

35. Forbes, D., et al., *Exercise programs for people with dementia.* Cochrane Database Syst Rev, 2015(4): p. Cd006489.

36. Groot, C., et al., *The Effect of Physical Activity on Cognitive Function in Patients with Dementia: A Meta-Analysis of Randomized Control Trials.* Ageing research reviews, 2015. **25**.

37. Ströhle, A., et al., *Drug and Exercise Treatment of Alzheimer Disease and Mild Cognitive Impairment: A Systematic Review and Meta-Analysis of Effects on Cognition in Randomized Controlled Trials.* Am J Geriatr Psychiatry, 2015. **23**(12): p. 1234-1249.

38. Corregidor-Sánchez, A.I., et al., *Effectiveness of virtual reality technology on functional mobility of older adults: systematic review and meta-analysis.* Age and Ageing, 2020. **50**(2): p. 370-379.

39. Gavelin, H.M., et al., *Combined physical and cognitive training for older adults with and without cognitive impairment: A systematic review and network meta-analysis of randomized controlled trials.* Ageing Res Rev, 2021. **66**: p. 101232.

40. Chan, J.S.Y., et al., *The effectiveness of dance interventions on cognition in patients with mild cognitive impairment: A meta-analysis of randomized controlled trials.* Neuroscience & Biobehavioral Reviews, 2020. **118**: p. 80-88.

41. Pacheco, T.B.F., et al., *Effectiveness of exergames for improving mobility and balance in older adults: a systematic review and meta-analysis.* Syst Rev, 2020. **9**(1): p. 163.

42. Zhu, Y., et al., *Effects of Aerobic Dance on Cognition in Older Adults with Mild Cognitive Impairment: A Systematic Review and Meta-Analysis.* J Alzheimers Dis, 2020. **74**(2): p. 679-690.

43. Wu, C., et al., *Effects of Mind-Body Exercises on Cognitive Function in Older Adults: A Meta-Analysis.* J Am Geriatr Soc, 2019. **67**(4): p. 749-758.

44. Stanmore, E., et al., *The effect of active video games on cognitive functioning in clinical and non-clinical populations: A meta-analysis of randomized controlled trials.* Neurosci Biobehav Rev, 2017. **78**: p. 34-43.

45. Howes, S.C., et al., *Gaming for Health: Systematic Review and Meta-analysis of the Physical and Cognitive Effects of Active Computer Gaming in Older Adults.* Physical Therapy, 2017. **97**(12): p. 1122-1137.

46. Gomes-Osman, J., et al., *Exercise for cognitive brain health in aging: A systematic review for an evaluation of dose.* Neurol Clin Pract, 2018. **8**(3): p. 257-265.
