# Supplementary material for: Making the Best Out of IT: Design and Development of Exergames for Older Adults With Mild Neurocognitive Disorder – A Methodological Paper
Source: Front Aging Neurosci. 2021 Dec 9;13:734012. doi: 10.3389/fnagi.2021.734012 (PMC8698204; doi:10.3389/fnagi.2021.734012)
Supplement: Supplementary file 2 [file Data_Sheet_2.pdf]

Supplementary Material

-

File 2 - Synthesis of evidence for possible ideas/concepts for the design of the remaining exergame parameters (i.e. complexity, progression & periodization, and variability/variation)

1 BIOLOOP:

Concept:

= Biocybernetic adaptation loop

"The biocybernetic loop is a modulation technique from the physiological computing field, which utilizes body signals in real-time to alter the system in order to assist users" [1-4]. *"This model of closed-loop control detects deviations from an optimal state of brain activity and uses these variations to cue changes at the human-computer interface in order to ''pull'' the psychological state of the user in a desired direction."* [5] In our case, we would define an optimal range ('the zone') of a correlate of brain activity (e.g. HRV) and the system would increase or decrease game demands to push participants into this optimal dosage based on real-time monitoring of a parameter for exergame demands. These parameters could be chosen to reflect the overall internal load or specific component of the exergame demands (i.e. cognitive and physical load).

Previous evidence:

Munoz et al. 2018: Developed a system for *"real-time adaptations in a custom exergame based on recommendations for targeted heart rate (HR) levels"* HOA. *"Results showed that the physiologically-augmented exergame leads players to exert around 40% more time in the recommended HR levels, compared to the conventional training, avoiding over exercising and maintaining good enjoyment levels."* [1]

Koenig et al. 2011: *"We provided healthy subjects and stroke patients with a virtual task during robot-assisted gait training, which allowed modulating cognitive load by adapting the difficulty level of the task. We quantified the cognitive load of stroke patients by using psychophysiological measurements and performance data." "We verified our classification results via questionnaires and obtained 88% correct classification in healthy subjects and 75% in patients. Using the pre-trained, adaptive classifier, we closed the cognitive control loop around healthy subjects and stroke patients by automatically adapting the difficulty level of the virtual task in real-time such that patients were neither cognitively overloaded nor under-challenged."* [6]

Ewing et al. 2015: Description of the Development and Validation Process of a Biocybernetic Loop for real-time adaption of game demand based on EEG monitoring. Depending on the loop-mechanisms, an increased alertness was found that produced the most desirable overall impact on player mood [5].

Advantages:

+ Approach is based on specific marker(s) of internal load [7-14] which offers a high level of personalization of exergame demands (depending on parameters used) to the individuals' capabilities that, in turn, allows to provide comparable inter-individual exercise doses which, in turn, would give more precise insights into dose-response relationships [7, 15]. Furthermore, internal training load determines training outcomes and was therefore recommended to use as a primary monitoring measure [16].
+ This approach was shown to increase alertness and the time spent at the optimal dose [1, 5]. Since adequate dose acts as an essential factor for triggering neurobiological processes [7] this approach would likely increase efficacy.
+ Could be combined with PERF (performance adaptation).
+ Easy to use for participants because the system would adapt automatically and we can easily monitor training dose (especially interesting for home-based applications).
+ Software made accessible by Munoz et el.: Biocybernetic Loop Engine by NeuroRehaLab.
+ Applicable for all types of populations.

Disadvantages:

- Large workload for the Dividat development team (but once done it could be broadly applied).
- Interface between Dividat software and monitoring tools has to be built.
- Necessity of monitoring tools (e.g. heart rate sensor) for personalized training (may limit applicability for home-based exergaming).
- Additional studies required to determine and validate the optimal parameter(s) before applying it in an intervention.

Open Questions:

? Which parameter could be used as an optimal measure of internal training load (i.e. should be easily applicable for monitoring internal training load, while also being valid and reliable)?
? What mechanisms will be used to adapt exergame demands (e.g. game complexity characterized by game speed, amount of distracting stimuli, contrasts of stimuli, game content)?
? Time-window that would be used to classify the individuals' internal load (e.g. rolling average of the last 20 seconds)?

Overall Applicability: limited

Necessary Preliminary Work:

Exemplary description of the development and validation process of a biocybernetic loop for real-time adaption of game demand based on EEG monitoring [5]: *"The design of a biocybernetic closed-loop incorporates a number of distinct processing stages: (1) data collection from sensors, (2) filtering of raw data coupled with artifact correction techniques, (3) data analysis for the extraction of meaningful metrics that permit a valid inference of the user state, (4) conversion of the metrics in order to instigate adaptation at the user interface, i.e., defining criteria/triggers for adaptation or by categorizing data using machine learning algorithms (Baldwin and Penaranda, 2012; Novak et al., 2012); and (5) adaptation of the user interface in a manner designed to promote a desirable user state."* [5]

Step 1: Formulation of a conceptual framework upon which to model the responses of the adaptive game (e.g. Motivational Intensity Theory [17] or the neurovisceral integration model [18-20])
Step 2: Operationalization of the model on basis of psychophysiological measures => Examine the most suitable parameter based on its sensitivity, reliability and validity.
Step 3: Development of the real-time adaptive gaming system => Define the optimal range (i.e. model should be able to classify the state of the participant) and implement the mechanisms used to push participants to this state by triggering changes in exergame demands (e.g. which parameters of the game are changed to increase or decrease the overall load, or the specific cognitive or physical loads).
Step 4: Evaluate the real-time adaptive model (i.e. is there an actual benefit compared to other exergaming systems => enjoyment, motivation, performance, training outcomes?)

2 PERF-LOOP:

Concept:

= Performance adaptation loop

Task level adaptation according to game performance in real-time

Previous evidence:

The performance adaptation loop provided by the Dividat Senso was applied in a qualitative study as well as a pilot RCT of Swinnen et al. 2020 and 2021 in older adults with major neurocognitive disorder. With a mean attendance rate of 82.9 %, the exergame-based intervention was well accepted in the pilot RCT [21]. Additionally, the exergame levels of intensity and complexity were reported to be agreeable in the qualitative study [22].

Advantages:

+ already implemented in Dividat Senso

Disadvantages:

- Game demands (i.e. motor- and cognitive demands) are linked to the physical demands because both change as a function of game complexity. Therefore, we don't know why exactly performance changes => Is it caused by cognitive, physical or any other factors?
- The qualitative study performed in this project revealed that this progression algorithm may not perform as intended in older adults with mild neurocognitive disorder and may thus lead to cognitive overload, which, needs to be avoided in this population.

Open Questions:

? How would it be possible to ensure a moderate physical exercise intensity when the task demands are adapted to game performance (which may primarily be related to cognitive demands, as older adults with mild neurocognitive disorder are cognitively impaired while having no major limitation in physical functions)?

Overall Applicability:

promising

Necessary Preliminary Work:

The performance of the performance adaptation loop provided by the Dividat Senso has to be evaluated and may have to be adapted to work properly in the population of older adults with mild neurocognitive impairment.

3 TARGINT:

Concept:	= Monitoring of target intensity<br><br>Intensity is displayed in real-time by monitoring of internal/external load. Participants have to change their behavior (e.g. increase stepping frequency) in order to reach target intensity. [23, 24]
Previous evidence:	Standard procedure for physical exercise and training prescription and monitoring (aerobic training) [25].
Advantages:	+ norm values and classification for physical exercise intensity available [25]
Disadvantages:	- applicability for HIIT (rocket) good, but challenging for other exergames<br>- only applicable for physical, but not for cognitive load
Open Questions:	? N/A
Overall Applicability:	Not applicable

4 PLAT:

Concept:	= Performance Plateau<br><br>Chosen games will be played until a performance plateau occurs. The occurrence of the performance plateau (after several training sessions) will mark an increase in external task demands (i.e. increase in game demands or the introduction of a new (slightly more difficult) game).
Previous evidence:	N/A
Advantages:	+ readily applicable<br>+ creates variance and contradicts boredom (since games are exchanged as soon as performance plateau occurs)
Disadvantages:	- Game demands (i.e. motor- and cognitive demands) are linked to the physical demands because both change as a function of game complexity. Therefore, we don't know why exactly performance changes => Is it caused by cognitive, physical or any other factors?
Open Questions:	? How do we define the performance plateau (e.g. integrated into Senso software to determine mathematically or visual determination of research team)?
Overall Applicability:	promising
Necessary Preliminary Work:	Definition of specific criteria to read out a plateau in game performance and progression rules to adapt the external task demands.

5 ADAPT:

Concept:	= Adaptation of intensity according to training progress<br><br>Target load is prescribed including a range (e.g. 3 x 10 repetitions per exercise, or 30 min at Borg 14 – 17) and as soon as the top range is achieved, the training load will be increased (e.g. external loads like exercise load or duration)
Previous evidence:	Standard procedure for physical exercise and training prescription and monitoring (resistance training) [25].
Advantages:	+ norm values and classification for physical exercise intensity available [25]
Disadvantages:	- Not applicable for exergaming with the Dividat Senso
Open Questions:	? N/A
Overall Applicability:	Not applicable

6 MYCHOICE:

Concept:	= Self-determined choice of games within groups of games for cognitive domains<br><br>Exergames will be grouped into the trained neurocognitive domains (e.g. learning and memory, executive function, complex attention, visuo-spatial skills) and each participant gets to choose which game within these groups he wants to choose.
Previous evidence:	N/A
Advantages:	+ promotes self-efficacy, which might have a positive influence on motivation [26]. According to the 'Optimizing Performance through Intrinsic Motivation and Attention for Learning (OPTIMAL)' theory of motor learning [27], this is expected to enhance performance expectancies which – accompanied with these autonomy-supportive conditions – "contribute to efficient goal-action coupling by preparing the motor system for task execution" [27]. This is further proposed "to facilitate the development of functional connectivity across brain regions, and structural neural connections more locally, that support effective and efficient motor performance and learning" [27, 28].
Disadvantages:	- might reduce variability of trained exergames within participants (since participants may choose their favorite game over and over again)<br>- increases variance in training content between participants => comparability should be more or less maintained since games are grouped into focus domains (e.g. executive function, attention, visuo-spatial skills, memory)
Open Questions:	? how would we integrate this scenario into the Dividat Software in case of home-based exergaming?
Overall Applicability:	limited

7 HRV-GUIDE:

Concept:	= HRV guided training prescription<br><br>High intensity training on days, when HRV is high (index for good recovery status).
Previous evidence:	Training prescription guided by resting HRV was already shown to enhance training effects of endurance training in younger adults in multiple systematic reviews [29-31].
Advantages:	+ personalized timing of high intensity sessions based on readiness and recovery status<br>+ resting HRV could be used to determine cognitive starting level of exergames
Disadvantages:	- Resting HRV is influence by numerous factors that don't necessarily relate to the recovery status or the readiness of participants for higher intensity exercises<br>- Participants would have to measure resting state HRV each morning or before the exergame sessions => are MCI patients capable of doing so?
Open Questions:	?
Overall Applicability:	limited

8 References

1. Muñoz, J., et al., *Closing the Loop in Exergaming - Health Benefits of Biocybernetic Adaptation in Senior Adults*. 2018. 329-339.
2. Fairclough, S. *A Closed-Loop Perspective on Symbiotic Human-Computer Interaction*. 2015. Cham: Springer International Publishing.
3. Pope, A., C. Stephens, and K. Gilleade, *Biocybernetic Adaptation as Biofeedback Training Method*. 2014. p. 91-115.
4. Pope, A.T., E.H. Bogart, and D.S. Bartolome, *Biocybernetic system evaluates indices of operator engagement in automated task.* Biological psychology, 1995. **40**(1-2): p. 187-195.
5. Ewing, K.C., S.H. Fairclough, and K. Gilleade, *Evaluation of an Adaptive Game that Uses EEG Measures Validated during the Design Process as Inputs to a Biocybernetic Loop.* Front Hum Neurosci, 2016. **10**: p. 223.
6. Koenig, A., et al., *Real-time closed-loop control of cognitive load in neurological patients during robot-assisted gait training.* IEEE transactions on neural systems and rehabilitation engineering : a publication of the IEEE Engineering in Medicine and Biology Society, 2011. **19**(4): p. 453-464.
7. Herold, F., et al., *Dose–Response Matters! – A Perspective on the Exercise Prescription in Exercise–Cognition Research.* Frontiers in Psychology, 2019. **10**(2338).
8. Ross, R., et al., *Precision exercise medicine: understanding exercise response variability.* British journal of sports medicine, 2019. **53**(18): p. 1141-1153.
9. Falck, R.S., et al., *Impact of exercise training on physical and cognitive function among older adults: a systematic review and meta-analysis.* Neurobiology of aging, 2019.
10. Etnier, J.L., E.S. Drollette, and A.B. Slutsky, *Physical activity and cognition: A narrative review of the evidence for older adults.* Psychology of Sport and Exercise, 2019. **42**: p. 156-166.
11. Erickson, K.I., et al., *Physical Activity, Cognition, and Brain Outcomes: A Review of the 2018 Physical Activity Guidelines.* Med Sci Sports Exerc, 2019. **51**(6): p. 1242-1251.
12. Cabral, D.F., et al., *Exercise for brain health: an investigation into the underlying mechanisms guided by dose.* Neurotherapeutics, 2019: p. 1-20.
13. Northey, J.M., et al., *Exercise interventions for cognitive function in adults older than 50: a systematic review with meta-analysis.* Br J Sports Med, 2018. **52**(3): p. 154-160.
14. Wasfy, M.M. and A.L. Baggish, *Exercise Dose in Clinical Practice.* Circulation, 2016. **133**(23): p. 2297-2313.
15. Stojan, R. and C. Voelcker-Rehage, *A Systematic Review on the Cognitive Benefits and Neurophysiological Correlates of Exergaming in Healthy Older Adults.* J Clin Med, 2019. **8**(5).
16. Impellizzeri, F., S. Marcora, and A. Coutts, *Internal and External Training Load: 15 Years On.* International Journal of Sports Physiology and Performance, 2019. **14**: p. 1-4.
17. J W Brehm, a. and E.A. Self, *The Intensity of Motivation.* Annual Review of Psychology, 1989. **40**(1): p. 109-131.
18. Thayer, J.F. and R.D. Lane, *A model of neurovisceral integration in emotion regulation and dysregulation.* J Affect Disord, 2000. **61**(3): p. 201-16.
19. Smith, R., et al., *The hierarchical basis of neurovisceral integration.* Neuroscience & biobehavioral reviews, 2017. **75**: p. 274-296.
20. Thayer, J.F., *Heart Rate Variability: A Neurovisceral Integration Model.* In R.S. Larry (Ed.), Encyclopedia of neuroscience, 2009: p. 1041-1047.

21. Swinnen, N., et al., *The efficacy of exergaming in people with major neurocognitive disorder residing in long-term care facilities: a pilot randomized controlled trial.* Alzheimer's Research & Therapy, 2021. **13**(1): p. 70.
22. Swinnen, N., et al., *Exergaming for people with major neurocognitive disorder: a qualitative study.* Disability and Rehabilitation, 2020: p. 1-9.
23. Panza, G.A., et al., *Can Exercise Improve Cognitive Symptoms of Alzheimer's Disease?* Journal of the American Geriatrics Society, 2018. **66**(3): p. 487-495.
24. Sanders, L.M.J., et al., *Dose-response relationship between exercise and cognitive function in older adults with and without cognitive impairment: A systematic review and meta-analysis.* PLoS One, 2019. **14**(1): p. e0210036.
25. Garber, C.E., et al., *American College of Sports Medicine position stand. Quantity and quality of exercise for developing and maintaining cardiorespiratory, musculoskeletal, and neuromotor fitness in apparently healthy adults: guidance for prescribing exercise.* Med Sci Sports Exerc, 2011. **43**(7): p. 1334-59.
26. Di Lorito, C., et al., *A scoping review of behaviour change theories in adults without dementia to adapt and develop the 'PHYT in dementia', a model promoting physical activity in people with dementia.* Maturitas, 2019. **121**: p. 101-113.
27. Wulf, G. and R. Lewthwaite, *Optimizing performance through intrinsic motivation and attention for learning: The OPTIMAL theory of motor learning.* Psychonomic Bulletin & Review, 2016. **23**(5): p. 1382-1414.
28. Lemos, A., et al., *Autonomy support enhances performance expectancies, positive affect, and motor learning.* Psychology of Sport and Exercise, 2017. **31**: p. 28-34.
29. Düking, P., et al., *Predefined vs. data guided training prescription based on autonomic nervous system variation: A systematic review.* Scandinavian Journal of Medicine and Science in Sports, 2020. **30**.
30. Granero Gallegos, A., et al., *HRV-Based Training for Improving VO2max in Endurance Athletes. A Systematic Review with Meta-Analysis.* International Journal of Environmental Research and Public Health, 2020. **17**: p. 7999.
31. Ruiz, J., et al., *Effectiveness of Training Prescription Guided by Heart Rate Variability Versus Predefined Training for Physiological and Aerobic Performance Improvements: A Systematic Review and Meta-Analysis.* Applied Sciences, 2020. **2020**: p. 8532.
