# Supplementary material for: Making the Best Out of IT: Design and Development of Exergames for Older Adults With Mild Neurocognitive Disorder – A Methodological Paper
Source: Front Aging Neurosci. 2021 Dec 9;13:734012. doi: 10.3389/fnagi.2021.734012 (PMC8698204; doi:10.3389/fnagi.2021.734012)
Supplement: Supplementary file 3 [file Data_Sheet_3.pdf]

Supplementary Material

-

File 3 – Brain-IT Training Concept

1 Training Characteristics

1.1 Overview

Frequency:	≥ 5x/week
Intensity/Complexity:	monitored and adapted according to predefined progression rules (section 5)
Type & Specificity:	exergame-based simultaneous incorporated multi-domain motor-cognitive training with an individualized (deficit-oriented) focus on (1) learning and memory, (2) executive function, (3) complex attention, and (4) visuo-spatial skills
Time & Duration:	≥ 21 min/session for 12 weeks
Volume:	≥ 105 min/week
Variability:	according to the concept of MYCHOICE (section 6)
Progression:	according to predefined progression rules (section 5)

Location:	at participant's homes
Guidance & Supervision:	structured in 3 phases starting with a guided familiarization period with the aim to lead participants to being able to train independently in the long-term

1.2 Description

The final training concept consists of an individually adapted multi-domain exergame-based simultaneous motor-cognitive training with incorporated cognitive tasks that will be adopted with a deficit-oriented focus on the neurocognitive domains of (1) learning and memory, (2) executive functioning, (3) complex attention, and (4) visuo-spatial skills. According to the training concept, each participant is instructed to train at least 5x/week for 21 min per session resulting in a weekly training volume of ≥ 105 min. All training sessions are planned to take place at participant's homes using the exergame train-ing system Dividat Senso Flex. In case of insufficient number of available home-based exergame training systems, the participants will be instructed to train at least 3x/week for 21 min per session at one of the study location sites using the exergame training system Dividat Senso (Dividat AG, Schindellegi, Switzerland; CE certification), but it is still recommended to train at the suggested optimal frequency (5x/week) and volume (105 min/week). In both cases, the system contains a pressure-sensitive platform (1.13 m × 1.13 m) that is sensitive to pressure changes (strain gauges measuring at 50 Hz) thereby detecting participants' position and timing of movements. The stepping platform is divided into five areas: (1) center (home position), (2) front, (3) right, (4) back, and (5) left. The platform will be connected via USB cable to the television screen of the participants (i.e. or the laboratory in case of using the Dividat Senso instead of Dividat Senso Flex). Weight-shifting and stepping movements to the four directions will enable the interaction and control of the virtual exergame scenarios that will be displayed right in front of the participant. Visual, auditory and somatosensory (vibrating platform) feedback will be provided in real-time in order to enrich the game experience.

The training intervention will start with a familiarization period of two weeks. During this phase, most of the training sessions (i.e. 4 out of 5 sessions) will be supervised by our research team. After this initial guided familiarization period, supervision of training sessions will be gradually reduced to 1x/week during a four-week transition phase. This transition phase aims to lead participants to being able to train independently. In this transition phase, the amount of supervision of training sessions will be individually determined within a predefined range (see Figure 1) in accordance with the capabilities and preferences of the participants. From the 7th week until completion of the training intervention, semi-autonomous training with one supervised training session per week will be prescribed for each participant. During independent training sessions, the research team will be available by phone to provide help when needed. In case the training sessions need to take place at at one of the study location sites using the exergame training system Dividat Senso, the absolute amount of supervision will be kept the same, since participants will be instructed to train at least 3x/week but it is still recommended to train at the suggested optimal frequency (5x/week) and volume (105 min/week).

2 Structure of each Exergame Session

Throughout the training intervention period, all sessions will be prescribed following the same basic structure: Each session consists of three blocks with 3 phases per block.

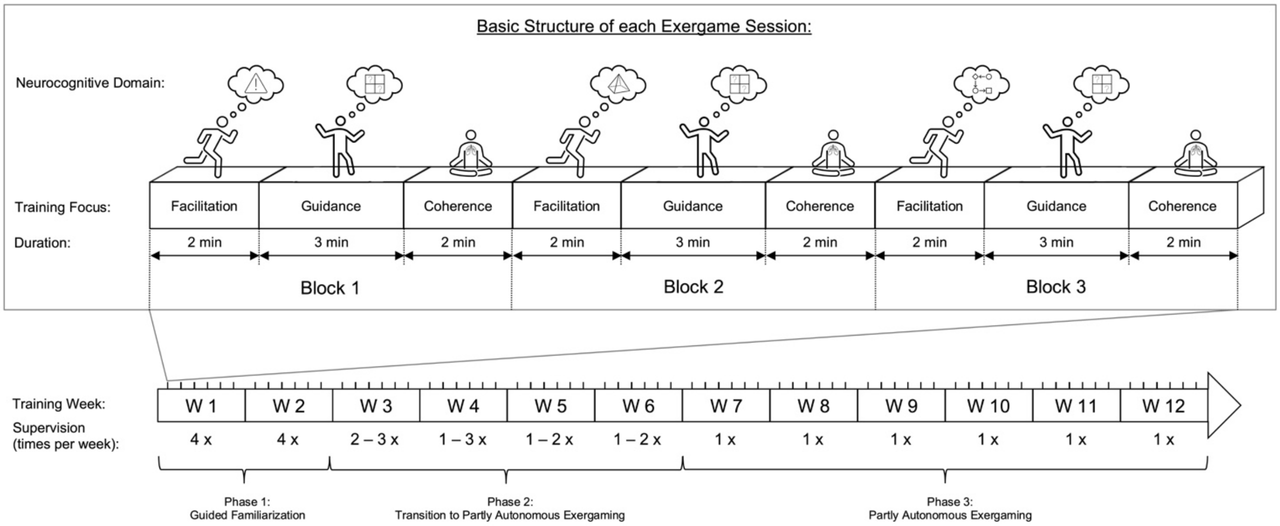

Figure 1: Overview of the exergame-based intervention concept and the basic structure of each exergame session (here as an example for a patient with amnestic-single domain mNCD with a training focus on learning and memory in week 1)

Phase 1 – Facilitation aims to apply a moderate physical intensity in the context of challenging but feasible neurocognitive and motoric demands mainly intending to "trigger neurophysiological mechanisms, which promote neuroplasticity" [1, 2] while additionally using "cognitive stimulation […] to "guide" these neuroplastic processes" [1-3]. This phase will include games focusing on neurocognitive domains that are least impaired. The external task demands will be individually adapted to ensure an appropriate internal training load. More specifically, the internal training load will be subdivided into a fixed component (i.e. physical intensity) and a variable component (i.e. neurocognitive (game-) demand). An additional stepping task will be used to set the level of physical intensity. It includes walking on the spot at a predefined stepping frequency that is needed to reach a moderate level of physical intensity (i.e. ranging between 40 and 59 % heart rate reserve (HRR) [4])). The stepping frequency will be individually determined for each participant (see section 5). A battery figure add-on is visible in the center of the screen that provides real-time visual feedback whether the predefined stepping frequency is reached. More specifically, if the predefined minimal required stepping frequency is reached or exceeded, the battery stays at equilibrium or fills. As long as the battery level is above 80 % (indicated by a line), the battery stays green. If the participants' stepping frequency falls below the predefined minimal required stepping frequency, the battery level decreases, and the battery turns orange (40 – 80 %) or red (below 40 %) indicating that the stepping frequency should be increased. On top of this fixed physical intensity, a variable amount of neurocognitive (game-) demands (e.g. game type, task complexity, predictability of required tasks) will be applied. Since the physical intensity is kept constant, changes in the overall internal training load can mainly be attributed to these neurocognitive and motoric (game-) demands and, accordingly, the internal training load can be adjusted on basis of these game characteristics. Therefore, the neurocognitive demands of the exergame will be individually adapted in order to ensure an appropriate total internal training load. The monitoring and adaption of the internal training load will be based on predefined progression rules for adapting characteristics of external training load (section 3).

Phase 2 – Guidance aims to make use of the triggered neurophysiological mechanisms from phase 1 to specifically guide neuroplastic processes of the mainly impaired neurocognitive domain. Therefore, games focusing on the mainly impaired neurocognitive domain for the individual participant (e.g. amnestic single domain => learning and memory) will be used. These games will solely focus on cognitive and motoric demands, but not on physical intensity. The cognitive-motoric demands of the exergame (also called 'external load') will be individually adapted in order to ensure an appropriate internal training load. The monitoring and adaption of the internal training load will be based on predefined progression rules for adapting characteristics of external training load (section 3).

Phase 3 – Coherence aims to implement a structured approach as a surrogate for the breaks between games. Patients with mNCD often exhibit depressive symptoms and anxiety, which are in turn important indicators for progression to dementia [198, 199]. To account for these psychological factors, resonance breathing guided by heart rate variability biofeedback (HRVB) will be used. HRVB training is a behavioral intervention aiming to increase cardiac autonomic control, to enhance homeostatic regulation, and to regulate emotional state [200, 201]. It consists of a regular breathing practice at a specific frequency that is individually determined that produces high amplitude of heart rate variability (HRV). Usually, this resonance breathing frequency is around 6 breaths/min [202]. An increased HRV is predicted to increase vagal afferent transmission to the forebrain, activate the prefrontal cortex, and improve executive function [200]. In fact, multiple systematic reviews and meta-analyses have indicated that HRVBT or paced breathing (at resonance frequency) is effective in decreasing depressive symptoms and anxiety in healthy adults and also clinical populations. Additionally, improved sleep quality, quality of life, HRV and brain activity in regions relevant for cognitive adaptations have been reported [203-205]. The evidence for older adults (i.e. ≥ 60 years) or patients with cognitive impairments is sparse, but decreases in depression, anxiety, and increases in attentional performance (no sign. difference in executive functioning) have already been reported, suggesting that older adults may benefit from HRVBT much like the younger populations [206]. Additionally, *"after initial training some people still achieve better results by following a heart monitor, while others do just as well doing paced breathing at their resonance frequency, once this frequency has been determined by biofeedback, following the second hand on a clock or counting seconds silently"* [4]. Therefore, for the sake of simplicity, we will make use of this transfer to resonance breathing. Before starting the training intervention, the resonance frequency is determined according to the protocol of Lehrer et al. 2013 (i.e. visit 1 of their protocol) [207]. During the training intervention, coherence breathing includes paced breathing for two minutes following the rhythm of the individually predetermined resonance frequency visualized on the screen of the exergame device (i.e. a sun is displayed within a landscape. When the sun gets bigger, the patients breath in. When the sun gets smaller, the patients breath out).

3 Overview of Exergames and Trained Neurocognitive Domains

In this section, an overview of the currently available exergames on the Dividat Senso (Flex) for the training focus on the neurocognitive domains of complex attention, learning and memory, executive function, and visuo-spatial skills that are used in this training concept is provided. Depending on the complexity of the games as such, an earliest start and latest end were predefined that will be considered in the progression rules (section 5).

Table 1: Overview of the Currently Available Exergames on the Dividat Senso (Flex) for the Training Focus on the Neurocognitive Domains of Complex Attention, Learning and Memory, Executive Function, and visuo-spatial skills
* = secondary classification of a game that focuses on more than one neurocognitive (sub)domain

Training Focus	Neurocognitive Domain	Neurocognitive Subdomain	Exergames	Timeframe	
				earliest start	latest end
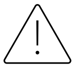	Complex Attention	Sustained Attention	'Simple'	W 1	W 8
		Divided Attention	'Divided'	W 2	W 10
		Selective Attention	'Birds'	W 3	W 12
			'Habitats'*	W 5	W 12
		Processing Speed	'Simple'*	W 1	W 8
			'Flexi'	W 4	W 12
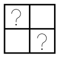	Learning & Memory AND Working Memory	Free Recall	'Shopping Tour'	W 1	W 12
		Serial Recall	'Simon'	W 3	W 12
			'Simon_numbered'	W 2	W 12
		Cued Recall	'Steps'	W 4	W 12
		Recognition Memory	'Shopping Tour'	W 1	W 12
		Semantic Memory	N/A	N/A	N/A
		Implicit Learning	N/A	N/A	N/A
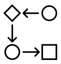	Executive Function	Working Memory	'Nomis_numbered'	W 5	W 12
			'Nomis'	W 6	W 12
		Planning	'Targets'*	W 1	W 12
			'Tetris'*	W 6	W 12
		Decision Making	N/A	N/A	N/A
		Inhibition	'Habitats'*	W 5	W 12
		Flexibility	'Flexi'	W 4	W 12
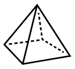	Visuo-spatial Skills	Visual Perception	'Targets'*	W 1	W 12
			'Tetris'*	W 6	W 12
		Visuoconstructional Reasoning	'Tetris'*	W 6	W 12
			N/A	N/A	N/A
		Perceptual-Motor Coordination	N/A	N/A	N/A

4 Description of Specific Exergames

Table 2: Description of the Currently Available Exergames on the Dividat Senso (Flex) for the Training Focus on the Neurocognitive Domains of Complex Attention, Learning and Memory, Executive Function, and Visuo-spatial Skills

colour codimng: black = existing games, green = new games or game elements

Neurocognitive Domain	Exergames				
	Name	Main Neurocognitive Subdomain(s)	Description	Parameters to adapt Task Complexity	Feedback Mechanisms (provided after each response)
Complex Attention 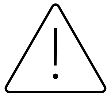	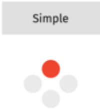	Sustained Attention, Processing Speed	In the game “Simple”, four circles are displayed in grey. As soon as one of the circles turns red, a step needs to be taken in the corresponding direction as fast as possible.	• game speed (interstimulus-interval)<br>• response window<br>• predictability (predefined vs. random sequences)<br>• stepping direction(s)<br>• stepping frequency	Positive feedback:<br>‘Positive’ fibration (i.e. single short pulse), visual feedback (i.e. wiggling of target, and sound effect (i.e. “ringing bell”)<br><br>Negative feedback:<br>‘Negative fibration’ (i.e. multiple strong pulses).
	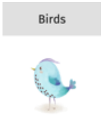	Selective Attention	In the game “Birds” a feather is displayed in the middle of the screen. The participants’ task is to match the feather with a bird and to return the feather to its birds by making a step into the corresponding direction.	• game speed (interstimulus-interval)<br>• response window<br>• stepping direction(s)<br>• predictability (predefined vs. random sequences)<br>• stepping frequency	Positive feedback:<br>‘Positive’ fibration (i.e. single short pulse), and sound effect (i.e. bird chirping).<br><br>Negative feedback:<br>‘Negative fibration’ (i.e. multiple strong pulses) and sound effect (i.e. muffled sound effect).
	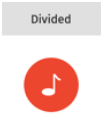	Divided Attention	In the game “Divided”, four circles are displayed in grey. As soon as one of the circles turns red, or an auditory cue is played (high tone = step forwards, low tone = step backwards), a step needs to be taken in the corresponding direction as fast as possible.	• game speed (interstimulus-interval)<br>• response window<br>• predictability (predefined vs. random sequences)<br>• stepping direction(s)<br>• stepping frequency	Positive feedback:<br>‘Positive’ fibration (i.e. single short pulse), visual feedback (i.e. wiggling of target, and sound effect (i.e. “ringing bell”).<br><br>Negative feedback:<br>‘Negative fibration’ (i.e. multiple strong pulses).

Learning and Memory & Working Memory (EF)<br>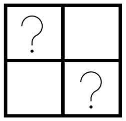	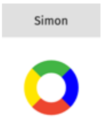	Serial Recall	In the game 'Simon', a stepping sequence (i.e. indicated by a concurrent lighting up of a sequence of sections with different colors of a circle and a corresponding sound) has to be memorized and repeated by stepping into the corresponding direction.	• sequence length<br>• stepping direction(s)<br>• stepping frequency	Positive feedback:<br>'Positive' fibration (i.e. single short pulse), visual feedback (i.e. lighting up of target, and sound effect (i.e. single tone corresponding to colour).<br><br>Negative feedback:<br>'Negative fibration' (i.e. multiple strong pulses) and sound effect (i.e. muffled sound effect).
	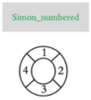	Serial Recall	In the game 'Simon_numbered', a stepping sequence (i.e. indicated by a concurrent lighting up of a sequence of sections with different numbers of a circle and a corresponding sound) has to be memorized and repeated by stepping into the corresponding direction.	• sequence length<br>• stepping direction(s)<br>• stepping frequency	Positive feedback:<br>'Positive' fibration (i.e. single short pulse), visual feedback (i.e. lighting up of target, and sound effect (i.e. single tone corresponding to colour).<br><br>Negative feedback:<br>'Negative fibration' (i.e. multiple strong pulses) and sound effect (i.e. muffled sound effect).
	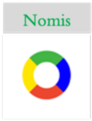	Working Memory	In the game 'Nomis' (Simon backwards), a stepping sequence (i.e. indicated by a concurrent lighting up of a sequence of sections with different colors of a circle and a corresponding sound) has to be memorized and repeated backwards by stepping into the corresponding direction.	• sequence length<br>• stepping direction(s)<br>• stepping frequency	Positive feedback:<br>'Positive' fibration (i.e. single short pulse), visual feedback (i.e. lighting up of target, and sound effect (i.e. single tone corresponding to colour).<br><br>Negative feedback:<br>'Negative fibration' (i.e. multiple strong pulses) and sound effect (i.e. muffled sound effect).
	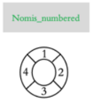	Working Memory	In the game 'Nomis_numbered', a stepping sequence (i.e. indicated by a concurrent lighting up of a sequence of sections with different numbers of a circle and a corresponding sound) has to be memorized and repeated backwards by stepping into the corresponding direction.	• sequence length<br>• stepping direction(s)<br>• stepping frequency	Positive feedback:<br>'Positive' fibration (i.e. single short pulse), visual feedback (i.e. lighting up of target, and sound effect (i.e. single tone corresponding to colour).<br><br>Negative feedback:<br>'Negative fibration' (i.e. multiple strong pulses) and sound effect (i.e. muffled sound effect).

	Steps 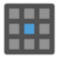	Cued Recall	In the Game ‘Steps’, a stepping sequence (i.e. indicated by a concurrent lighting up of a sequence of sections with different numbers of a square with 9 fields) has to be memorized and repeated by stepping into the corresponding direction at the rhytm of the beat (i.e. indicated by a metronome).	• number of steps per level<br>• maximal number of trials per stepping sequence<br>• start level	Positive feedback:<br>‘Positive’ fibration (i.e. single short pulse), visual feedback (i.e. lighting up of target (in green), and sound effect (i.e. single tone corresponding to a number and ascending tone sequence).<br><br>Negative feedback:<br>visual feedback (i.e. lighting up of target (in orange/red), and sound effect (i.e. descending tone sequence).
	Shopping Tour 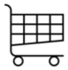	Free Recall, Recognition Memory	At first, a shopping list is displayed in the center of the screen for the duration of encodiung phase. Second, the shopping list will disappear and one item after another will appear on the screen. The users’ task is to gather all items (type and quantity) of the shopping list by stepping to the right (i.e. to put the object into the shopping cart) or to the left (i.e. not to buy the product). After each response, a feedback is provided.	• number of items on the list<br>• number of items to be purchased<br>• probability of presented items to be purchased or not (in percent)	Positive feedback:<br>Arrow lights up in green light, ‘positive’ (i.e. single short pulse) fibration and sound effect (i.e. “ringing bell”)<br><br>Negative feedback:<br>Arrow lights up in red light, ‘negative fibration’ (i.e. multiple strong pulses), and sound effect (i.e. muffled sound effect).
Executive Function 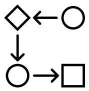	Flexi 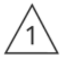	Flexibility, Processing Speed	Part A: The game “Flexi” requires participants to make a step in the direction of the next higher number, starting from the number displayer in the center.<br>Part B: In addition, a figure appears around the number. It is necessary to make a step in the direction of the next higher number with the opposite pattern.	• task complexity (Part A/B)	Positive feedback:<br>‘Positive’ fibration (i.e. single short pulse), and sound effect (i.e. “ringing bell”).<br><br>Negative feedback:<br>‘Negative fibration’ (i.e. multiple strong pulses).
	Habitats 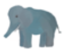	Inhibition, Selective Attention	In the game “Habitats”, animals move across the four landscapes in the picture. If an animal does not appear in its familiar surroundings, a step needs to be taken in this direction. However, animals shouldn’t be disturbed in their natural habitat.	• game speed (interstimulus-interval)<br>• task complexity (including inhibition tasks or not)<br>• stepping direction(s)<br>• stepping frequency	Positive feedback:<br>‘Positive’ fibration (i.e. single short pulse), visual feedback (i.e. wiggling of target, and sound effect (i.e. animal sounds).<br><br>Negative feedback:<br>‘Negative fibration’ (i.e. multiple strong pulses), sound effect (i.e. muffled sound effect), and visual animation (i.e. animal with speech bubble displaying “Hey!”)

Visuo-Spatial Skills 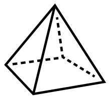	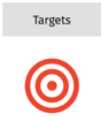	Planning, Visual Perception	'Targets' requires the participant to hit incoming red balls in the middle of the target by stepping into the corresponding direction.	• game speed (speed multiplier)<br>• stepping direction(s)<br>• stepping frequency	<u>Positive feedback:</u> 'Positive' fibration (i.e. single short pulse), visual feedback (i.e. visual impulse of selected target, and sound effect (i.e. "ringing bell").<br><br><u>Negative feedback:</u> 'Negative fibration' (i.e. multiple strong pulses) and sound effect (i.e. muffled sound effect).
	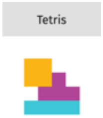	Visuoconstructional Reasoning, Planning	'Tetris' requires participants to rotate and move two-dimensional polygons (with varying shapes and colors) dropping one-by-one from top to the bottom. The aim of the game is to arrange complete rows of blocks to form solid horizontal lines, in order to let these lines disappear.	• game speed (speed multiplier)<br>• stepping frequency	<u>Positive feedback:</u> 'Positive' fibration (i.e. multiple short pulses) for a full row, visual feedback (i.e. full row disappears), and sound effect (i.e. "ringing bell").<br><br><u>Negative feedback:</u> none

5 Progression Rules for Monitoring Internal Load and Adapting External Loads

5.1 Phase 1 – Facilitation

As described above, the internal training load will be subdivided into the physical exercise intensity of the stepping task and the neurocognitive and motoric (game-) demands of the games in phase 1. Additionally, the level of stability support (holding on to a handrail or similar with both hands, one hand, only two fingers or no stability support) will be in dividually determined to reach a challenging but safe condition. The stepping frequency of the stepping tasks will be predetermined for each participant with the aim to reach a moderate level of physical intensity (i.e. ranging between 40 and 59 % heart rate reserve (HRR) [4]). To avoid overload, the participants will be introduced stepwise; first, the stepping frequency will be determined while the level of neurocognitive demand is held low. Afterwards, the total level of internal training load will be monitored and continuously adapted.

Phase 1a – Determination of minimal stepping frequency:

All participants will start with a stepping frequency of 100 steps/min and at Level 1 of game demands in the first training session (see section 7). The target physical exercise intensity is determined based on the target heart rate (HR) that is calculated using the Karvonen method with a target intensity of 40 % HRR: $HR_{target} = (HR_{max} - HR_{rest}) \cdot 0.40 + HR_{rest}$ [15, 16]. For this calculation the age-predicted maximal heart rate: $HR_{max} = 208 - 0.7 \cdot \text{age}$ and HR_{rest} measured at the pre-measurements will be used. The stepping frequency will then be increased by 5 steps/min at each training session until the minimal level of physical exercise intensity is reached. The evaluated stepping frequency will then be considered as a fixed component of the overall external load. In all subsequent training session, this fixed physical exercise intensity will be kept constant and the focus shifts on monitoring and adapting the total internal training load.

Phase 1b – Monitoring and adaptation of total internal training load:

Since the physical intensity in phase 2 is held constant, changes in the overall internal training load can mainly be attributed to the variable level of neurocognitive demand. The level of neurocognitive demand will be standardized according to predefined game levels (see section 7). Phase 2 will be continued with game level 1, until a plateau in performance is reached. A plateau in performance will be read out visually guided by the following predefined criteria: (1) a performance increase of less than or equal to 5 % compared to the previous exergame session while (2) there was an increase in performance from session so session over at least the previous three training sessions. The specific performance outcomes for each exergame to take into consideration are unterlined in table 3. In case a precision outcome is available (i.e. for the game 'Targets'; precision = number of hits / (number of hits + number of missed targets)), the criterion to progress to the next higher level is to achieve a precision of at least 95 %. Each time a plateau in performance is reached, the game level will be increase by one level. Additionally, the level of stability support (holding on to a handrail or similar with both hands, one hand, only two fingers or no stability support) will be individually determined to reach a challenging but safe condition. Depending on the complexity of the games as such, the earliest start and latest end that were predefined in section 2 that will additionally have to be considered when planning the training sessions.

5.2 Phase 2 – Guidance

In phase 2, the mainly impaired neurocognitive domain will be trained. Therefore, the focus of monitoring and adapting the task demands will solely focus on neurocognitive demands (i.e. motor- and cognitive demands that are linked because both change as a function of game complexity). The level of neurocognitive demand will be standardized according to predefined game levels (see section 8) for game levels one to nine. The final game level (i.e. Level 10+) will be based on an adaptive mode (i.e. performance adaptation loop provided by the Dividat Senso) that aims to adapt the game demands in order to provide an optimal challenge. All participants will start with level 1. Each time a pletau in performance is reached, the game level will be increase by one level. A plateau in performance will be read out visually guided by the following predefined criteria: (1) a performance increase of less than or equal to 5 % compared to the previous exergame session while (2) there was an increase in performance from session so session over at least the previous three training sessions. The specific performance outcomes for each exergame to take into consideration are unterlined in table 4. In case a precision outcome is available (i.e. for the games 'Shopping Tour' and 'Targets' (precision = number of hits / (number of hits + number of missed targets))), the criterion to progress to the next higher level is to achieve a precision of at least 95 %. Additionally, the level of stability support (holding on to a handrail or similar with both hands, one hand, only two fingers or no stability support) will be individually determined to reach a challenging but safe condition. Depending on the complexity of the games as such, the earliest start and latest end that were predefined in section 2 that will additionally have to be considered when planning the training sessions.

6 The concept of MYCHOICE to ensure sufficient variability

The concept of MYCHOICE describes a self-determined choice of exergames within groups of games for cognitive domains so that the preferences of each participant can be taken into account while the time spent at training each neurocognitive domain is still standardized within participants with the same training focus (i.e. predetermined according to the deficit-oriented focus on the neurocognitive domains as described in section 2). The advantage of this concept is that it promotes self-efficacy, which might have a positive influence on training motivation [17]. According to the Optimizing Performance through Intrinsic Motivation and Attention for Learning (OPTIMAL) theory of motor learning, this is expected to enhance performance expectancies which – accompanied with these autonomy-supportive conditions – "contribute to efficient goal-action coupling by preparing the motor system for task execution" [18]. This is further proposed "to facilitate the development of functional connectivity across brain regions, and structural neural connections more locally, that support effective and efficient motor performance and learning" [18, 19]. With this regard, the exergames were grouped into mainly trained neurocognitive domains oflearning and memory, executive function, complex attention, visuo-spatial skills (see table S1) and each participant gets to choose which game within these groups he prefers to play. Optimally, the participant would get the option to choose between different games on the screen before starting each training session. Since this is not (yet) implemented into the Dividat user interface, alternatively, the research team will consecutively plan the training session for each participant based on his/her preferences.

Depending on the complexity of the games as such, the earliest start and latest end that were predefined in section 2 that will have to be considered when planning the training sessions. Therefore, the range for self-determined choices is limited at the start of the training intervention with the aim to provide a certain routine until the participants have familiarized themselves with the game scenarios and are prepared to learn new games step-by-step. Over the course of the training intervention, the number of

options to choose from will steadily increase, giving the participants and the research team the opportunity to plan the training sessions according to the individuals' preferences.

7 Game Levels for Phase 1 – Facilitation

Table 3: Game Levels for each Game for Phase 1 - Facilitation

Game	Task Demands												Performance Measures
	Parameter(s)		Conditions & Settings										
			Level 1	Level 2	Level 3	Level 4	Level 5	Level 6	Level 7	Level 8	Level 9	Level 10	
Simple 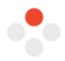	game speed (interstimulus-interval)		= ½ RW	= ½ RW	= ½ RW	= ½ RW	= ½ RW	= ½ RW	= ½ RW	= ½ RW	= ½ RW	= ½ RW	• mean reaction time<br>• game score<br>• point rate
	response window (RW)		10,000 ms	8,000 ms	6,000 ms	5,000 ms	4,000 ms	3,000 ms	2,500 ms	2,000 ms	1,500 ms	1,000 ms	
	predictability (order/time interval)		random	random	random	random	random	random	random	random	random	random	
	stepping direction(s)	↑	90 %	80 %	70 %	60 %	50 %	45 %	40 %	35 %	30 %	25 %	
		→	5 %	10 %	15 %	20 %	22.5 %	22.5 %	25 %	25 %	25 %	25 %	
		←	5 %	10 %	15 %	20 %	22.5 %	22.5 %	25 %	25 %	25 %	25 %	
		↓	0 %	0 %	0 %	0 %	5 %	10 %	10 %	15 %	20 %	25 %	
Divided 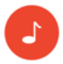	game speed (interstimulus-interval)		= ½ RW	= ½ RW	= ½ RW	= ½ RW	= ½ RW	= ½ RW	= ½ RW	= ½ RW	= ½ RW	= ½ RW	• mean reaction time<br>• game score<br>• point rate
	response window (RW)		8,000 ms	6,000 ms	4,000 ms	3,500 ms	3,000 ms	2,500 ms	2,000 ms	1,500 ms	1,000 ms	750 ms	
	predictability (order/time interval)		random	random	random	random	random	random	random	random	random	random	
	stepping direction(s)	↑	80 %	70 %	60 %	55 %	50 %	45 %	40 %	35 %	30 %	25 %	
		→	10 %	15 %	20 %	20 %	22.5 %	22.5 %	25 %	25 %	25 %	25 %	
		←	10 %	15 %	20 %	20 %	22.5 %	22.5 %	25 %	25 %	25 %	25 %	
		↓	0 %	0 %	0 %	5 %	5 %	10 %	10 %	15 %	20 %	25 %	
Simon 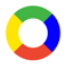	sequence lenght		2	3	3	4	4	5	5	6	6	7	• mean reaction time<br>• game score<br>• point rate
	stepping direction(s)	↑	50 %	50 %	35 %	35 %	25 %	35 %	25 %	35 %	25 %	25 %	
		→	25 %	25 %	25 %	25 %	25 %	25 %	25 %	25 %	25 %	25 %	
		←	25 %	25 %	25 %	25 %	25 %	25 %	25 %	25 %	25 %	25 %	
		↓	0 %	0 %	15 %	15 %	25 %	15 %	25 %	15 %	25 %	25 %	
Simon_numbered 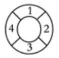	sequence lenght		2	3	3	4	4	5	5	6	6	7	• mean reaction time<br>• game score<br>• point rate
	stepping direction(s)	↑	50 %	50 %	35 %	35 %	25 %	35 %	25 %	35 %	25 %	25 %	
		→	25 %	25 %	25 %	25 %	25 %	25 %	25 %	25 %	25 %	25 %	
		←	25 %	25 %	25 %	25 %	25 %	25 %	25 %	25 %	25 %	25 %	
		↓	0 %	0 %	15 %	15 %	25 %	15 %	25 %	15 %	25 %	25 %	
Nomis 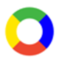	sequence lenght		2	3	3	3	4	4	5	5	6	6	• mean reaction time<br>• game score<br>• point rate
	stepping direction(s)	↑	50 %	50 %	35 %	25 %	35 %	25 %	35 %	25 %	35 %	25 %	
		→	25 %	25 %	25 %	25 %	25 %	25 %	25 %	25 %	25 %	25 %	
		←	25 %	25 %	25 %	25 %	25 %	25 %	25 %	25 %	25 %	25 %	
		↓	0 %	0 %	15 %	25 %	15 %	25 %	15 %	25 %	15 %	25 %	

Nomis_numbered 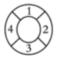	sequence lenght		2	3	3	3	4	4	5	5	6	6	• mean reaction time<br>• game score<br>• point rate
	stepping direction(s)	↑	50 %	50 %	35 %	25 %	35 %	25 %	35 %	25 %	35 %	25 %	
		→	25 %	25 %	25 %	25 %	25 %	25 %	25 %	25 %	25 %	25 %	
		←	25 %	25 %	25 %	25 %	25 %	25 %	25 %	25 %	25 %	25 %	
		↓	0 %	0 %	15 %	25 %	15 %	25 %	15 %	25 %	15 %	25 %	
Habitats 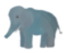	game speed (interstimulus-interval)		10,000 ms	8,000 ms	6,000 ms	5,000 ms	4,000 ms	3,500 ms	3,000 ms	2,500 ms	2,000 ms	1,500 ms	• mean reaction time<br>• game score<br>• point rate
	response window (RW)		constant	constant	constant	constant	constant	constant	constant	constant	constant	constant	
	task complexity (including inhibition task = yes/no)		no	no	no	yes	yes	yes	yes	yes	yes	yes	
	stepping direction(s)	↑	80 %	70 %	60 %	55 %	50 %	45 %	40 %	35 %	30 %	25 %	
		→	10 %	15 %	20 %	20 %	22.5 %	22.5 %	25 %	25 %	25 %	25 %	
		←	10 %	15 %	20 %	20 %	22.5 %	22.5 %	25 %	25 %	25 %	25 %	
		↓	0 %	0 %	0 %	5 %	5 %	10 %	10 %	15 %	20 %	25 %	
Targets 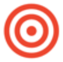	game speed (speed multiplier)		0.2	0.25	0.3	0.35	0.4	0.45	0.5	0.55	0.6	0.65	• game score<br>• point rate<br>• number of hits<br>• number of missed tar-gets
	stepping direction(s)	↑	90 %	80 %	70 %	60 %	50 %	45 %	40 %	35 %	30 %	25 %	
		→	5 %	10 %	15 %	20 %	22.5 %	22.5 %	25 %	25 %	25 %	25 %	
		←	5 %	10 %	15 %	20 %	22.5 %	22.5 %	25 %	25 %	25 %	25 %	
		↓	0 %	0 %	0 %	0 %	5 %	10 %	10 %	15 %	20 %	25 %	
Tetris 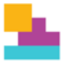	game speed (speed multiplier)		0.6	0.8	1	1.2	1.3	1.4	1.5	1.6	1.7	1.8	• game score

8 Game Levels for Phase 2 – Guidance

Table 4: Game Levels for each Game for Phase 2 - Guidance

Game	Task Demands												Performance Measures
	Parameter(s)		Conditions & Settings										
			Level 1	Level 2	Level 3	Level 4	Level 5	Level 6	Level 7	Level 8	Level 9	Level 10(+)	
Simple	game speed (interstimulus-interval)		= ½ RW	= ½ RW	= ½ RW	= ½ RW	= ½ RW	= ½ RW	= ½ RW	= ½ RW	= ½ RW	= ½ RW	• mean reaction time<br>• game score<br>• point rate
	response window (RW)		5,000 ms	4,000 ms	3,000 ms	2,500 ms	2,000 ms	1,750 ms	1,500 ms	1,250 ms	1,000 ms	adaptive; start level: 1,000 ms	
	predictability (order/time interval)		random	random	random	random	random	random	random	random	random	random	
	stepping direction(s)	↑	90 %	80 %	70 %	60 %	50 %	45 %	40 %	35 %	30 %	25 %	
		→	5 %	10 %	15 %	20 %	22.5 %	22.5 %	25 %	25 %	25 %	25 %	
		←	5 %	10 %	15 %	20 %	22.5 %	22.5 %	25 %	25 %	25 %	25 %	
		↓	0 %	0 %	0 %	0 %	5 %	10 %	10 %	15 %	20 %	25 %	
Birds	game speed (interstimulus-interval)		= ½ RW	= ½ RW	= ½ RW	= ½ RW	= ½ RW	= ½ RW	= ½ RW	= ½ RW	= ½ RW	= ½ RW	• mean reaction time<br>• game score<br>• point rate
	response window (RW)		8,000 ms	6,000 ms	4,000 ms	3,500 ms	3,000 ms	2,500 ms	2,000 ms	1,500 ms	1,000 ms	adaptive; start level: 1,000 ms	
	predictability (order/time interval)		random	random	random	random	random	random	random	random	random	random	
	stepping direction(s)	↑	80 %	70 %	60 %	55 %	50 %	45 %	40 %	35 %	30 %	25 %	
		→	10 %	15 %	20 %	20 %	22.5 %	22.5 %	25 %	25 %	25 %	25 %	
		←	10 %	15 %	20 %	20 %	22.5 %	22.5 %	25 %	25 %	25 %	25 %	
		↓	0 %	0 %	0 %	5 %	5 %	10 %	10 %	15 %	20 %	25 %	
Divided	game speed (interstimulus-interval)		= ½ RW	= ½ RW	= ½ RW	= ½ RW	= ½ RW	= ½ RW	= ½ RW	= ½ RW	= ½ RW	= ½ RW	• mean reaction time<br>• game score<br>• point rate
	response window (RW)		3,000 ms	2,750 ms	2,500 ms	2,250 ms	2,000 ms	1,750 ms	1,500 ms	1,250 ms	1,000 ms	adaptive; start level: 1,000 ms	
	predictability (order/time interval)		random	random	random	random	random	random	random	random	random	random	
	stepping direction(s)	↑	80 %	70 %	60 %	55 %	50 %	45 %	40 %	35 %	30 %	25 %	
		→	10 %	15 %	20 %	20 %	22.5 %	22.5 %	25 %	25 %	25 %	25 %	
		←	10 %	15 %	20 %	20 %	22.5 %	22.5 %	25 %	25 %	25 %	25 %	
		↓	0 %	0 %	0 %	5 %	5 %	10 %	10 %	15 %	20 %	25 %	
Simon	sequence lenght		2	3	4	5	5	6	6	7	7	8	• mean reaction time<br>• game score<br>• point rate
	stepping direction(s)	↑	50 %	50 %	35 %	35 %	25 %	35 %	25 %	35 %	25 %	25 %	
		→	25 %	25 %	25 %	25 %	25 %	25 %	25 %	25 %	25 %	25 %	
		←	25 %	25 %	25 %	25 %	25 %	25 %	25 %	25 %	25 %	25 %	
		↓	0 %	0 %	15 %	15 %	25 %	15 %	25 %	15 %	25 %	25 %	

Simon_numbered	sequence lenght		2	3	4	5	5	6	6	7	7	8	• mean reaction time<br>• game score<br>• point rate
	stepping direction(s)	↑	50 %	50 %	35 %	35 %	25 %	35 %	25 %	35 %	25 %	25 %	
		→	25 %	25 %	25 %	25 %	25 %	25 %	25 %	25 %	25 %	25 %	
		←	25 %	25 %	25 %	25 %	25 %	25 %	25 %	25 %	25 %	25 %	
		↓	0 %	0 %	15 %	15 %	25 %	15 %	25 %	15 %	25 %	25 %	
Nomis	sequence lenght		2	3	3	3	4	4	5	5	6	6	• mean reaction time<br>• game score<br>• point rate
	stepping direction(s)	↑	50 %	50 %	35 %	25 %	35 %	25 %	35 %	25 %	35 %	25 %	
		→	25 %	25 %	25 %	25 %	25 %	25 %	25 %	25 %	25 %	25 %	
		←	25 %	25 %	25 %	25 %	25 %	25 %	25 %	25 %	25 %	25 %	
		↓	0 %	0 %	15 %	25 %	15 %	25 %	15 %	25 %	15 %	25 %	
Nomis_numbered	sequence lenght		2	3	3	3	4	4	5	5	6	6	• mean reaction time<br>• game score<br>• point rate
	stepping direction(s)	↑	50 %	50 %	35 %	25 %	35 %	25 %	35 %	25 %	35 %	25 %	
		→	25 %	25 %	25 %	25 %	25 %	25 %	25 %	25 %	25 %	25 %	
		←	25 %	25 %	25 %	25 %	25 %	25 %	25 %	25 %	25 %	25 %	
		↓	0 %	0 %	15 %	25 %	15 %	25 %	15 %	25 %	15 %	25 %	
Steps	progression rule defined by the game itself		This game was designed to include over 100 game levels considering a progression in motor load (i.e. execution speed (i.e. stepping frequency (beats per minute) and pattern complexity), and cognitive load (i.e. pattern length) that are described by Giannouli et al. 2020 [20]. All participants will start at level 1 and each training session will start at the final level of the previous training session.										• game score<br>• point rate<br>• number of hits<br>• number of missed targets<br>• accuracy
Shopping Tour	number of items on the list		2	3	3	4	4	5	5	6	6	7	• mean reaction time<br>• number of items collected<br>• number of mistakes<br>• precision
	duration of encoding phase		10 s	8 s	6 s	8 s	6 s	7.5 s	5 s	9 s	6 s	7 s	
	bulking probability		0 %	80 %	60 %	80 %	60 %	60 %	40 %	50 %	40 %	50 %	
	probability of presented items to be purchased or not (in percent)		80 %	70 %	60 %	55 %	50 %	45 %	40 %	35 %	30 %	25 %	
Flexi	task complexity		Part A	Part A & B									• mean reaction time<br>• game score
Habitats	game speed (interstimulus-interval)		8,000 ms	6,000 ms	4,000 ms	3,500 ms	3,000 ms	2,500 ms	2,000 ms	1,500 ms	1,000 ms	adaptive; start level: 1,000 ms	• mean reaction time<br>• game score<br>• point rate
	response window (RW)		constant	constant	constant	constant	constant	constant	constant	constant	constant	constant	
	task complexity (including inhibitio task = yes/no)		no	no	no	yes	yes	yes	yes	yes	yes	yes	
	stepping direction(s)	↑	80 %	70 %	60 %	55 %	50 %	45 %	40 %	35 %	30 %	25 %	
		→	10 %	15 %	20 %	20 %	22.5 %	22.5 %	25 %	25 %	25 %	25 %	
		←	10 %	15 %	20 %	20 %	22.5 %	22.5 %	25 %	25 %	25 %	25 %	
		↓	0 %	0 %	0 %	5 %	5 %	10 %	10 %	15 %	20 %	25 %	

Targets	game speed (speed multiplier)		0.4	0.5	0.6	0.7	0.8	0.85	0.9	0.95	1.00	adaptive; start level: 1.00	• game score<br>• point rate<br>• number of hits<br>• number of missed targets
	stepping direction(s)	↑	90 %	80 %	70 %	60 %	50 %	45 %	40 %	35 %	30 %	25 %	
		→	5 %	10 %	15 %	20 %	22.5 %	22.5 %	25 %	25 %	25 %	25 %	
		←	5 %	10 %	15 %	20 %	22.5 %	22.5 %	25 %	25 %	25 %	25 %	
		↓	0 %	0 %	0 %	5 %	5 %	10 %	10 %	15 %	20 %	25 %	
Tetris	game speed (speed multiplier)		1	1.2	1.3	1.4	1.5	1.6	1.7	1.8	1.9	adaptive; start level: 2.00	• game score

9 References

1. Herold, F., et al., *Thinking While Moving or Moving While Thinking – Concepts of Motor-Cognitive Training for Cognitive Performance Enhancement.* Frontiers in Aging Neuroscience, 2018. **10**(228).
2. Fissler, P., et al., *Novelty interventions to enhance broad cognitive abilities and prevent dementia: synergistic approaches for the facilitation of positive plastic change.* Progress in brain research, 2013. **207**: p. 403-434.
3. Bamidis, P., et al., *A review of physical and cognitive interventions in aging.* Neuroscience & Biobehavioral Reviews, 2014. **44**: p. 206-220.
4. Garber, C.E., et al., *American College of Sports Medicine position stand. Quantity and quality of exercise for developing and maintaining cardiorespiratory, musculoskeletal, and neuromotor fitness in apparently healthy adults: guidance for prescribing exercise.* Med Sci Sports Exerc, 2011. **43**(7): p. 1334-59.
5. Ismail, Z., et al., *Prevalence of Depression in Patients With Mild Cognitive Impairment: A Systematic Review and Meta-analysis.* JAMA Psychiatry, 2017. **74**(1): p. 58-67.
6. Ma, L., *Depression, Anxiety, and Apathy in Mild Cognitive Impairment: Current Perspectives.* Frontiers in aging neuroscience, 2020. **12**: p. 9-9.
7. Shaffer, F., R. McCraty, and C.L. Zerr, *A healthy heart is not a metronome: an integrative review of the heart's anatomy and heart rate variability.* Front Psychol, 2014. **5**: p. 1040.
8. Lehrer, P.M. and R. Gevirtz, *Heart rate variability biofeedback: how and why does it work?* Frontiers in psychology, 2014. **5**: p. 756-756.
9. Schwerdtfeger, A.R., et al., *Heart rate variability (HRV): From brain death to resonance breathing at 6 breaths per minute.* Clin Neurophysiol, 2020. **131**(3): p. 676-693.
10. Lehrer, P., et al., *Heart Rate Variability Biofeedback Improves Emotional and Physical Health and Performance: A Systematic Review and Meta Analysis.* Appl Psychophysiol Biofeedback, 2020. **45**(3): p. 109-129.
11. Zaccaro, A., et al., *How Breath-Control Can Change Your Life: A Systematic Review on Psycho-Physiological Correlates of Slow Breathing.* Frontiers in human neuroscience, 2018. **12**: p. 353-353.
12. Goessl, V.C., J.E. Curtiss, and S.G. Hofmann, *The effect of heart rate variability biofeedback training on stress and anxiety: a meta-analysis.* Psychol Med, 2017. **47**(15): p. 2578-2586.
13. Jester, D.J., E.K. Rozek, and R.A. McKelley, *Heart rate variability biofeedback: implications for cognitive and psychiatric effects in older adults.* Aging & Mental Health, 2019. **23**(5): p. 574-580.
14. Lehrer, P., et al., *Protocol for Heart Rate Variability Biofeedback Training.* Biofeedback, 2013. **41**: p. 98-109.
15. Karvonen, M.J., E. Kentala, and O. Mustala, *The effects of training on heart rate - a longitudinal study.* Annales medicinae experimentalis et biologiae Fenniae, 1957. **35**(3): p. 307.

16. Karvonen, J. and T. Vuorimaa, *Heart rate and exercise intensity during sports activities.* Sports medicine, 1988. **5**(5): p. 303-311.

17. Di Lorito, C., et al., *A scoping review of behaviour change theories in adults without dementia to adapt and develop the 'PHYT in dementia', a model promoting physical activity in people with dementia.* Maturitas, 2019. **121**: p. 101-113.

18. Wulf, G. and R. Lewthwaite, *Optimizing performance through intrinsic motivation and attention for learning: The OPTIMAL theory of motor learning.* Psychonomic Bulletin & Review, 2016. **23**(5): p. 1382-1414.

19. Lemos, A., et al., *Autonomy support enhances performance expectancies, positive affect, and motor learning.* Psychology of Sport and Exercise, 2017. **31**: p. 28-34.

20. Giannouli, E., T. Morat, and W. Zijlstra, *A Novel Square-Stepping Exercise Program for Older Adults (StepIt): Rationale and Implications for Falls Prevention.* Frontiers of Medicine, 2020. **6**: p. 318.
